# Terminology spectrum analysis of natural-language chemical documents: term-like phrases retrieval routine

**DOI:** 10.1186/s13321-016-0136-4

**Published:** 2016-04-29

**Authors:** Boris L. Alperin, Andrey O. Kuzmin, Ludmila Yu. Ilina, Vladimir D. Gusev, Natalia V. Salomatina, Valentin N. Parmon

**Affiliations:** Boreskov Institute of Catalysis SB RAS, Pr. Lavrentieva 5, Novosibirsk, Russia 630090; Novosibirsk State University, Pirogova 2, Novosibirsk, Russia 630090; Sobolev Institute of Mathematics SB RAS, Acad. Koptyug Avenue 4, Novosibirsk, Russia 630090

**Keywords:** Terminology spectrum, Natural language text analysis, n-Gram analysis, Term-like phrases retrieval, Text information retrieval

## Abstract

**Background:**

This study seeks to develop, test and assess a methodology for automatic extraction of a complete set of ‘term-like phrases’ and to create a terminology spectrum from a collection of natural language PDF documents in the field of chemistry. The definition of ‘term-like phrases’ is one or more consecutive words and/or alphanumeric string combinations with unchanged spelling which convey specific scientific meanings. A terminology spectrum for a natural language document is an indexed list of tagged entities including: recognized general scientific concepts, terms linked to existing thesauri, names of chemical substances/reactions and term-like phrases. The retrieval routine is based on n-gram textual analysis with a sequential execution of various ‘accept and reject’ rules with taking into account the morphological and structural information.

**Results:**

The assessment of the retrieval process, expressed quantitatively with a precision (P), recall (R) and F_1_-measure, which are calculated manually from a limited set of documents (the full set of text abstracts belonging to 5 EuropaCat events were processed) by professional chemical scientists, has proved the effectiveness of the developed approach. The term-like phrase parsing efficiency is quantified with precision (P = 0.53), recall (R = 0.71) and F_1_-measure (F_1_ = 0.61) values.

**Conclusion:**

The paper suggests using such terminology spectra to perform various types of textual analysis across document collections. This sort of the terminology spectrum may be successfully employed for text information retrieval, for reference database development, to analyze research trends in subject fields of research and to look for the similarity between documents.

**Graphical abstract:**
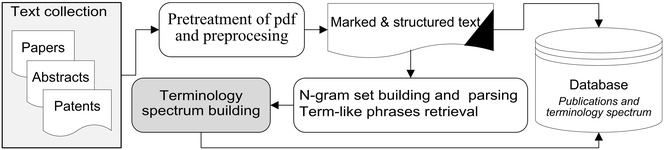
Terminology spectrum building process with term-like phrases retrieval

**Electronic supplementary material:**

The online version of this article (doi:10.1186/s13321-016-0136-4) contains supplementary material, which is available to authorized users.

## Background

The current situation in chemistry, as in any other field of natural science, can be characterized by a substantial growth of texts in natural languages (research papers, conference proceedings, patents, etc.), still being the most important sources of scientific knowledge and experimental data, information about modern research trends and terminology used in the subject areas of science. It greatly increases the value of such powerful information systems as Scopus^®^, SciFinder^®^, Reaxys^®^ which are capable of handling large text document databases and especially those fitted with advanced text information retrieval capabilities. In fact, both efficiency and productivity of modern scientific research in chemistry depend rigorously on quality and completeness of its information support, which is oriented firstly on advanced and flexible reference search, discovering and analysing of text information to afford the most relevant answers to user questions (substances, reactions, relevant patents or journal articles). The main ideas and developments in the information retrieval methods coupled with techniques of full text analysis are now well described and examined [1].

In conventional information systems, the majority of text information retrieval and discovery methods are based on using specific sets of pre-defined document metadata, e.g. keywords or indexes of terms characterizing the texts content. User queries are converted using index into information requests expressed by Boolean terms combination with bringing into play the vector space and terms weight. Probabilistic approaches may also be employed to take into account such features as terms distribution, co-occurrence information and their relationships derived from information retrieval thesauri (IRT) to include them into analytic process. Any kind of such indexes have to be produced and updated mainly manually by trained experts, but now the possibilities of automated indexes development attract closer attention.

It is assumed that the structural foundation of any scientific text is its terminology basis, which may be represented, in principle, by advanced IRT. However, it leads to difficulties in applying conventional IRTs in practical information text analysis procedures because of limitations inherent in them. Typically, such thesauri are made manually in a very labor-intensive process and often are constructed to reflect the general terminology only. Terms from thesauri originally represent a formally written description of scientific conceptions and definitions which may not exactly match the real usage and spelling used in scientific texts. Moreover, a thesaurus developed for one type of text may be less efficient or not applicable when used with another. A good example is the IUPAC “Gold Book” [2] compendium of chemical nomenclature, terminology, units and definition recommendations. Terminology drafted by experts of IUPAC spans a wide range of chemistry but does not describe any field in detail and represents only a well-established upper level of scientific terminology. Summarizing, IRT based text analysis alone is unable to solve the problem of the variability of scientific texts written in natural languages because the accuracy of matching thesaurus terms with real text phrases leaves much to be desired.

It should also be noted that the language of science is evolving faster than that of natural language, especially in chemistry and molecular biology. Thus, the analysis of terminology basis of subject text collection should be done automatically using both primitive extraction and sophisticated knowledge-based parsing. Only automated data analysis can process and reveal the variety of term-like word combinations in constantly changing world of scientific publications. Automated parsing and analysis of document collections or isolated documents for term-like phrases can also help to discover various contexts in which the same scientific terminology is used in different publications or even parts of the same publication.

There is nothing new in the idea of automated terms retrieval. Typically, the terminology analysis of text content is focused on recognition of chemical entities and automatic keyphrase extraction aimed to provide a limited set of keywords which might characterize and classify the document as a whole. Two main strategies are usually applied: machine self-learning and usage of various dictionaries with automated selection rules (heuristics) coupled with calculated features [3], such as TF-IDF [4, 5]. Therefore, keyphrase retrieval procedures typically involve the following stages: initial text preprocessing; selecting a candidate to a keyphrase; applying rules to each candidate; compiling a list of keyphrases [6]. A few existing systems had been analyzed in terms of precision (P), recall (R) and F_1_-score attainable for existing keyphrase extraction datasets. For such well-known systems as Wingnus, Sztergak, KP-Mminer these values are reported as P = 0.34÷0.40, R = 0.11÷0.14, F_1_ = 0.17÷0.20 [6]. Open-Source Chemistry Analysis Routines (OSCAR4) [7] and ChemicalTagger [8] NLP may also be mentioned as tools for the recognition of named chemical entities and for parsing and tagging the language of text publications in chemistry.

However, there are some inherent shortcomings in the above mentioned keyphrase extraction approaches due to the presence of a significant amount of cases where a limited set of automatically selected top ranked keyphrases does not properly describe the document in details (e.g., a paper may contain the description of a specific procedure of catalyst preparation while not being the main subject of the paper). It may also be seen from the aforementioned values of P, R and F that in many cases the extracted keyphrases do not match the keyphrases selected by experts to an adequate degree. Exact matching of keyphrases is a rather rare event, partially due to the difficulties of taking into account nearly similar phrases, for instance, semantically similar phrases. On the other hand, even though the widely used n-gram analysis can bild a full spectrum of token sequences present in the text, it may also produce a great level of noise, making it difficult to use them. Some attempts have been made to take into account the semantic similarity of n-grams and to differentiate between rubbish and candidates to plausible keyphrases [9, 10].

The problem of automatic recognition of scientific terms in natural language texts has been explored during last decades [11]. It is shown that taking into account the linguistic information may improve the terms extraction efficiency. The information about grammatical structure of multiword scientific terms, their text variants, context of their usage may be represented as a set of lexico-syntactic patterns. For instance, the values of P, R and F-measure equal to 73.1, 53.6 and 61.8 % respectively for term extraction from scientific texts (only in Russian) on computer science and physics were obtained [12].

A ‘terminology spectrum’ of a natural language publication may be defined as an indexed list of tagged token sequences with calculated weights, such as recognized general scientific notions, terms linked to existing thesauri, names of chemical entities and ‘term-like phrases’. The term-like phrases are not exactly the keyphrases or terms in the usual sense (like published in thesauri). Such term-like phrases are defined here as one or more consecutive tokens (represented by words and/or alphanumeric strings combinations), which convey specific scientific meaning with unchanged spelling and context as in a real text document. For instance, a term-like phrase may look similar to a specific generally used term but with different spelling or word order reflecting the usage of the term in a different context in natural language environment. Consequently, they may describe real text content and the essence of real processes that the scientific research handles, which makes the analysis of such phrases extremely useful. That sort of terminology spectrum of a natural language publication may be considered as some kind of knowledge representation of a text and may be successfully employed in various information retrieval strategies, text analysis and reference systems [13].

The present work is aimed to develop and test the methodology of automated retrieval of full terminology spectrum from any natural language chemical text collections in pdf format, with term-like phrases selection being the central part of the procedure. The retrieval routine is based on n-gram text analysis with sequential execution of a complex of ‘accept’ and ‘reject’ rules while taking into account the morphological and structural information. The term ‘n-gram’ denotes here a text string or a sequence of *n* consecutive words or tokens presented in a text. Numerical assessment of automated term-like phrases retrieval process efficiency done in the paper is calculated by comparing automatically extracted term-like phrases and those manually selected by experts.

## Methods

### Text collection used for experiments

Chemical catalysis is a foundation of chemical industry and represents a very complex field of scientific and technological researches. It includes chemistry, various subject fields of physics, chemical engineering, material science and a lot of more. One of the most representative research conferences in catalysis is «European Congress on Catalysis—EuropaCat», which has been chosen as a source of scientific texts covering the wide range of themes of researches. A set of abstracts of EuropaCat conferences of 2013, 2011, 2009, 2007, 2005 (about 6000 documents in all five Congress events) has been used for textual analysis in the present study. All abstracts are in pdf format.

### General description of terminology spectrum retrieval process

**Fig. 1:**
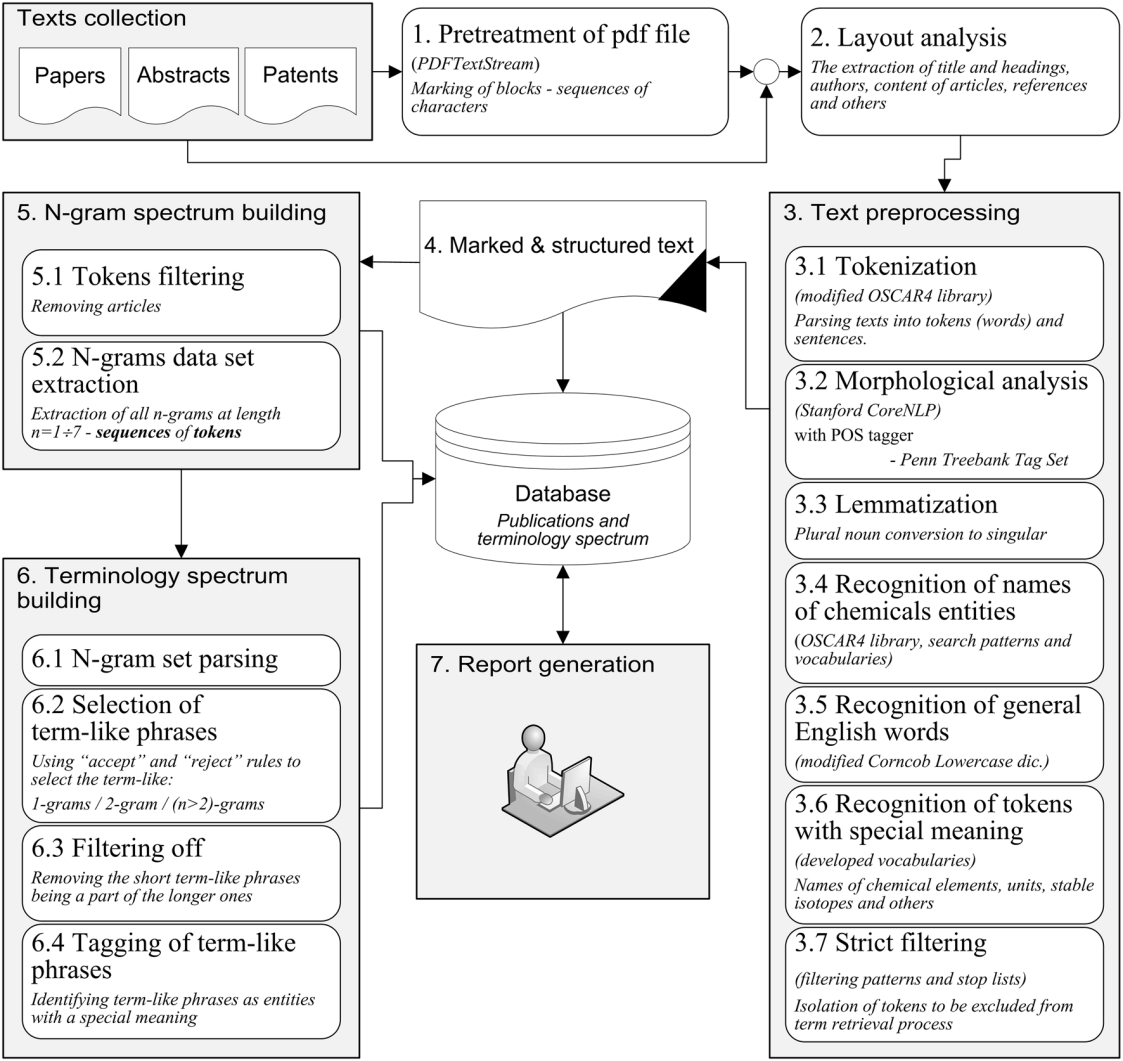
General scheme of the terminology spectrum building process with term-like phrases retrieval

The developed system of terminology spectrum analysis consists of the following sequentially running procedures or steps, as depicted in Fig. 1.

The server side of the terminology spectrum analysis system runs on Java SE 6 platform and the client is a PHP web-application to view texts and the results of terminology analysis. To store all data collected in the terminology retrieval process the cross-platform document-oriented database MongoDB is used [14]. The choice in favor of MongoDB was conditioned by the need to process nested n-gram structures up to level 7.

The main stages and analytic methods involved in the process are discussed in the following sections.

### Text materials conversion with PdfTextStream library [15]

**Fig. 2:**
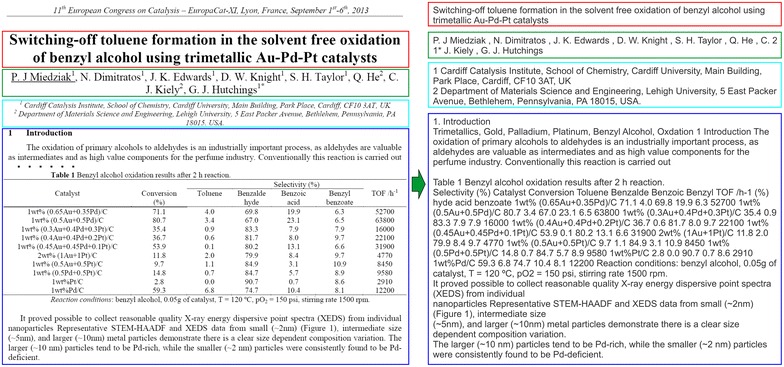
An example of pdf-to-text transformation

The scientific texts are mainly published in pdf format which does not typically contain any information about document structure and therefore is not suitable for immediate text analysis. Thus, at first, a document has to be preprocessed by converting a pdf file into the text format and analyzing its structure (highlighting titles, authors, headings, references, etc.) with the aim to make the text suitable for further content information retrieval (see Fig. 2). The following steps are used (stages 1–2 on Fig. 1) to make such kind of pdf transformation (for a detailed example see Additional File 1):

1. Isolation of text blocks which have the same formatting (e.g. bold, underline and etc.);
2. Removing empty blocks and merging blocks located on the same text row;
3. Analyzing the document structure by classifying each block as containing information about the publication title, the headings, the authors, the organizations, the e-mails, the references and the content. To perform such analysis a set of special taggers has been developed which are executed sequentially to analyze and tag each text block. Taggers utilize such features as the position of first and last rows of text block, text formatting, a position of a block of text on a page, etc. All developed taggers have been adjusted to handle each conference event individually.
4. Text block filtration to remove unclassified text blocks, for instance, situated before the publication title, because such blocks typically contain useless and already known information about a conference or journal.
5. Unification of special symbols (such as variants of dash, hyphen, and quote characters), removal of space characters placed before brackets in writings of crystal indexes, etc. Regular expressions are used.

### Text preprocessing

The text preprocessing stage #3 in Fig. 1 is to transform a text document obtained from stages 1–2 into a unified structured format with markup. During this stage the text is split into individual words and sentences (tokenization) followed by a morphological analysis that includes: highlighting objects such as formulas and chemical entities, removing unnecessary words and meaningless combinations of symbols, recognizing general English words and tokens with special meaning (units, stable isotopes, acronyms, etc.). The result of this stage is a fully marked structured text to be stored in the database. The following steps are involved in the text preprocessing stage.

#### Tokenization

A tokenizer from the OSCAR4 library is used for splitting a text into words, phrases and other meaningful elements. The tokenizer has been adapted for better handling of chemical texts.

**Fig. 3:**
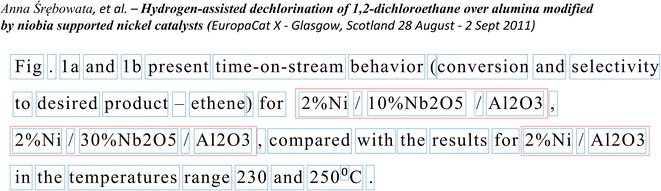
An example of the tokenization process. Frames outline the results of modified OSCAR4 tokenizer, additional outer frames isolate tokens describing a chemical composition (possessing the tag “COMP”)

The present study established that the original OSCAR4 tokenizer, in view of our needs, has some shortcomings. First one is a separation of tokens with a hyphen “-”, which often leads to mistakes in recognizing compound terms. To overcome this issue, the parts of the source code which are responsible for splitting tokens with hyphens were commented out (see Additional File 2). The next resolved problem is that some complex tokens, representing various chemical compositions, are considered by the tokenizer as a sequence of tokens (see Fig. 3). In such cases it is necessary to combine these isolated tokens into an integral one. The modified tokenizing procedure makes merging of tandem tokens separated with either “/” or “:” characters, provided that they are marked by OSCAR4 tag «CM» or incorporate a chemical element symbol sign. In addition, tokens looking as “number %” and situated at the beginning of a such phrase describing chemical compositions are merged into the integral token too (see Fig. 3).

An example of the work of the modified tokenizer is shown on Fig. 3. Blue frames hold the tokens identified by modified OSCAR4 tokenizer. Additional red frames outline tokens which are combined into integral ones. Such tokens are marked with the isolated tag «COMP». This tag is used by accept rule «ChemUnigramRule» to identify one-word n-grams describing chemical compositions.

Then the position of a token in the text is determined. Splitting series of tokens into sentences finalizes the tokenization process, which is realized with the help of WordToSentenceAnnotator routine of Stanford CoreNLP library [16, 17].

#### Morphological analysis and labeling tokens with their POS tags

Morphological analysis (Stanford CoreNLP library [18] is used) maps each word with a set of part-of-speech tags (Penn Treebank Tag Set [19] by Stanford CoreNLP is used). Typical tags used in the research are: «NN» (« NNS »)—nouns; «VB»—verb; «JJ»—adjective; «CD»—ordinal numeral, etc. For the full information about the POS tags used by terminology spectrum building procedure see Table 4.

#### Lemmatization

Lemmatization is the process of grouping together different inflected word forms so they can be treated as a single item. But, in the present work, lemmatization is only used to replace nouns in the plural form with their lemmas. Preliminary experiments demonstrate that additional lemmatization is not helpful and leads to a significant loss of meaningful information (for example, «*reforming process*» leads to «*reform*» and «*process*» lemmas with the loss of the name of a very important modern industrial chemical process in refinery).

#### Recognition of names of chemical entities

Meta-information about names of chemical entities is very important in various term-like phrases retrieval strategies. The open source OSCAR4 (Open Source Chemistry Analysis Routines) [7, 20] software package is applied for selection and semantic annotation of chemical entities across a text. Among a variety of tags and attributes utilized by OSCAR4 routine only the following ones are used in the present study:

1. CM—chemical term (chemical name, formula or acronym);
2. RN—reaction (for example, «epoxidation», «dehydrogenation», «hydrolysis», etc.);
3. ONT—ontology term (for example, «glass», «adsorption», «cation», etc.).

When a token is a part of some recognized chemical entity the token gets the same OSCAR4 tag as a whole entity.

#### Recognition of tokens with special meaning

The significant part of text preprocessing stage is selection of individual tokens being the words of general English and recognition of various meaningful text strings which are: the general scientific terms (actually performed at the final «terminology spectrum building stage» but described here for convenience); tokens denoting chemical elements, stable isotopes and measurement units; tokens which cannot be a part of any terms in any way. This part of work is performed using specially developed dictionaries described in details in Table 1.

**Table 1:** Developed/modified dictionaries used for recognition of general English words, general chemical science terms and tokens with special meaning

Dictionary/*Usage for*	Description	Reference	Examples
General chemical science terms *Selection of general terms (chemical and from related fields of physics, mathematics …)*	~7500 General scientific terms in chemistry, physics and mathematics IUPAC Compendium is used	http://goldbook.iupac.org/ IUPAC Compendium of Chemical Terminology (Gold Book)	Naphthenes, solvation energy, osmotic pressure, reaction dynamics …
General English words dictionary *Selection of general English words*	~58,000 general English words. It is based on Corncob Lowercase Dictionary modified by us for stated goals. 566 words were excluded, which are often used in scientific terminology	The modified by us the Corncob- Lowercase list of more than 57,000 English words http://ru.scribd.com/doc/147594864/ Corncob-Lowercase (see Additional file 3 for excluded words)	Abbreviate, academic, accelerate … ***Excluded*** *Abrasion, absorption, aerosol* …
Stop list *Filtering tokens which are not part of terms* *in any way*	~2060 tokens. List contains the words, abbreviations and so on, which cannot be incorporated into any term-like phrases	Proprietary design (see Additional file 4)	e.g., de, ca., fig., al., co-exist, et, etc., i.e., ltd …
Stable isotopes *Filtering n*-*grams containing digits*	~250 isotopes. It is based on The Berkeley Laboratory Isotopes Project’s isotopes database	Proprietary design, based on The Berkeley Laboratory Isotopes Project’s DB. http://ie.lbl.gov/education/isotopes.htm (see Additional file 5)	1H, 2H, 3He, 4He, 6Li, 7Li …
Chemical elements signs *Filtering n*-*grams containing digits*	~126 chemical elements. It is based on Periodic table	Proprietary design, based on periodic table (see Additional file 6)	H, He, Li, Be, B, C, N, O, F …
Measurement units *Filtering n*-*grams containing units of measure*	~100 records now. It is partially based on IUPAC GoldBook	Proprietary design, partially based on http://goldbook.iupac.org/ (see Additional file 7)	(a.u.), (ev), a.u, °C, ppm, kV, mol, g^−1^, ml^−1^, gcat, gcat h …

Some extra explanation needs to be given on the general English dictionary, the stop list dictionary and the procedure of recognition of general scientific terms.

More than 560 words either found in scientific terminology (for instance: “acid”, “alcohol”, “aldehyde”, “alloy”, “aniline”, etc.) or occurring in composite terms (for example, “abundant” may be part of the term “most abundant reactive intermediates”) were excluded from the original version of Corncob Lowercase Dictionary.

The IUPAC GoldBook Compendium on chemical terminology (the only well-known and time-proven dictionary) is used as a source of general chemistry terms. To find the best way to match an n-gram to a scientific term from the Compendium a number of experiments have been performed which resulted in the following criteria:

1. N-gram is considered a general scientific term if all n-gram tokens are the words of a certain IUPAC Goldbook term, regardless of their order;
2. If (n − 1) of n-gram tokens coincide with the (n − 1) words of an IUPAC Goldbook term and the remaining word is among other terms in the dictionary, then the n-gram is considered a general scientific term too.

Some examples may be given. The n-gram “RADIAL CONCENTRATION GRADIENT” is a general scientific term because the phrase “concentration gradient” is in the Compendium and the word “radial” is part of the term “radial development”. The n-gram “CONTENT CATALYTIC ACTIVITY” is a general term because the term “catalytic activity content” is present in the Compendium and differs from the n-gram only by word order. The n-gram “TOLUENE ADSORPTION CAPACITY” is not considered a general term, despite the fact that two words coincide with the term “absorption capacity”, because the remaining word “TOLUENE” is special and is not found in the Compendium. The n-gram “COBALT ACETATE DECOMPOSITION” is not considered a general term either as only the term “decomposition” may be found.

The final comment is about the stop list dictionary that, at first glance, may look like a set of arbitrary words. But, actually, it is based on a series of observations performed with the set of wrongly identified term-like phrases by the earlier version of the terminology analysis system.

#### Strict filtering

**Fig. 4:**
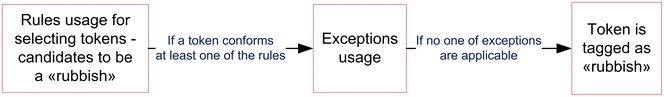
General scheme of strict filtering tagging

The last but not least step in the text preprocessing stage is strict filtering developed to remove unnecessary words and meaningless combinations of symbols. If at least one of n-gram tokens is labeled by the strict filtering tag (“rubbish”: “true”) then such n-gram is not considered a term-like phrase. At this stage, certain character sequences as described by the filtering rules (Table 2) and not exempt by the list of exceptions (Table 3) are looked for. They are successive digits, special symbols, measurement units, symbols of chemical elements, brackets and so on. Custom regular expressions and standard dictionaries described in Table 1 are used for this procedure. A general scheme of strict filtering parsing is illustrated in Fig. 4.

**Table 2:** Rules for strict filtering procedure

No.	Rule	Examples
1	***SpecialSymbolsRule*** True, if a token contains at least one of the special symbols different from: . -,/: () [] + = @ ^®^	SIZE(**), SELECTIVITY%, NIMG_650, H2S↔35SCAT, 1AUDAE_AM, ΔGADS, H0 ≦−8.2
2	***StopListRule*** True, if a token is in the stop list (Table 1)	LITERATURE, VIEWPOINT, PERCENT, PRESENT, IMPORTANCE, FUNDAMENTAL, CONCLUSION, TYPICALLY, EXAMPLE, INTRODUCTION
Rules of regular expressions: True, if a token satisfies at least one of the regular expressions from the following list		
3	***4DigitRule*** True, if a token contains four or more digits in succession	FQM-3994, RYC-2008-03387, 20000H-1, MAT2010-21147, CO(0001)-CARBIDE, CO(111)/CO(0001), RU(0001) ELECTRODE
4	***3DigitRule*** True, if a token contains three digits in succession	215KMTA, 220ML, 148H-1, CU2O(111), AU{111}-CEO2{100}, MGO/AG(100)
	***2DigitRule*** True, if a token begins with one or two digits	12C16O-13C16O, 31P{1H}, 2-PROPANOL, 2-METHYL-1-BUTENE, 3-METHYL-1,3-BUTADIENE, 15 %H3PW12O40/TIO2
5	***UnitsRule*** True, if a token ends with a string from the dictionary of measurement units (Table 1)	KJMOL-1, MMOL.MIN-1, KJ.MOL-1, G.GZEOLITE-1.H-1, CM3.MIN-1.G-1

**Table 3:** Exceptions for strict filtering procedure

No.	Exception	Examples
1	***Facet_Index_4digits*** Token denotes the substance containing a 4-digits facet index. The list of chemical element signs is used (Table 1)	*terms*: RU(0001); CO(0001)-CARBIDE; α-FE2O3(0001) *rubbish*: HPG1800B; RYC-2008-03387; 20000H-1
2	***Miller_Index_3digits*** Token denotes the substance containing a 3-digits crystallographic Miller index. The list of chemical element signs is used	*terms*: CEO2(111); PT(111); AU{111}-CEO2{100}; (NI,AL)(111); AL2O3/NIAL(110) *rubbish*: R873; 50WX8-100; 270-470OC
3	***Substances_3digits*** Token denotes chemical containing 3 digits in succession. Chemical elements signs list and regular expressions as «EL/\{\d{3}\}» are used	*terms*: 15N218O; H235S; H218O-SSITKA; H216O/H218O *rubbish*: FA100; TSVET-500; CE-440
4	***Isotopes*** Token denotes an isotope. Stable isotopes and chemical elements signs lists are used (Table 1)	*terms*: 13C CP-MAS NMR; 12C16O-13C16O MIXTURE; 31P MAS NMR SPECTROSCOPY *rubbish*: 04,21H; 11H; 11HV; 1 %18O2; -1H-1; 57CO
5	***Substances_2digits*** Token denotes substance, which begins with one or two digits	*terms*: 5-PENTANEDIOL; 2-AMINOBENZENE-1,4-DICARBOXYLATE; 5-BROMO-3-(N,N-DIETHYLAMINO-ETHOXY)-2-METHYLINDOLE *rubbish*: 2R,3S; 2LFH; 5NICZPOL; 1KPM; 4-CP
6	***Catalysts*** Token denotes a catalytic system which is a chemical composition with «.» character	*terms*: 1.5AU/C; 1.0CUCOK/ZRO2; CE0.9PR0.1O2; CU0.2CO0.8FE2O4; MG3ZN3.-XFE0.5AL0.5; LAFE0.7NI0.3O3-Δ; CE0.8GD0.2O2-Δ; MN0.8ZR0.2 *rubbish*: VOL. %; (B)2.5 %; DISP.[%]
7	***Comp*** Token denotes the chemical or catalyst composition. Tag «COMP» is used	*terms*: 20 %CU/ZNAL; 0.4 %PD/AL2O3; 4 %PT-4 %RE/TIO2; (5 %)PB(10 %)-SBA15 *rubbish*: 50 %AIR; 1.5 %WT; 0-2.5MOL %; CA.23 %
8	***Cryst_hydrates*** Tokens denote crystalline hydrates. Regular expressions as «*[A-Za-z].*H_2_O$» are used	*terms*: AL(NO_3_)3*6H_2_O; FE2(SO4)3.9H_2_O; AUCL4(NH4)7[TI2(O2)2(CIT)(HCIT)]2.12H_2_O; *rubbish*: 0.6 %H_2_O; 0.03 %C3H6; 0.06286*T;
9	***SpatialDimension*** Token denotes the 1-, 2 - or 3-dimensional method or pattern	*terms*: 2D-SAXS; 2D-GC; 1D-3D COPPER – OXIDE; 1D-STRUCTURE; 1D COPPER – OXIDE *rubbish*: 12-MR; 1LATTICE; 16ACR; 60HPW
10	***Names*** Token denotes a proper name. A set of regular expressions is used for recognition	*terms*: BRØNSTED ACID; BRӦNSTED BASIC SITE; MӦSSBAUER SPECTROSCOPY; *rubbish*: L’ARGENTIЀRE; PROCESS’S
11	***OscarTags*** True, if a token has any Oscar tag and matches the following regular expressions: «\-[A-Za-z]{2}»; «\{«, «\[*[A-Za-z]» and etc	*terms*: STEM-HAADF; L-CYSTINE; DI-TERT-BUTYLPEROXIDE;[AU(EN)2]2[CU(OX)2]3 *rubbish*: 128°- Y-ROTATED; π- BACKDONATION; CONVERSION(%);CU(1)MN; M1(2); ACTIVITY [2]

*EL* designation of any chemical element, *IS* designation of any stable isotope

**Fig. 5:**
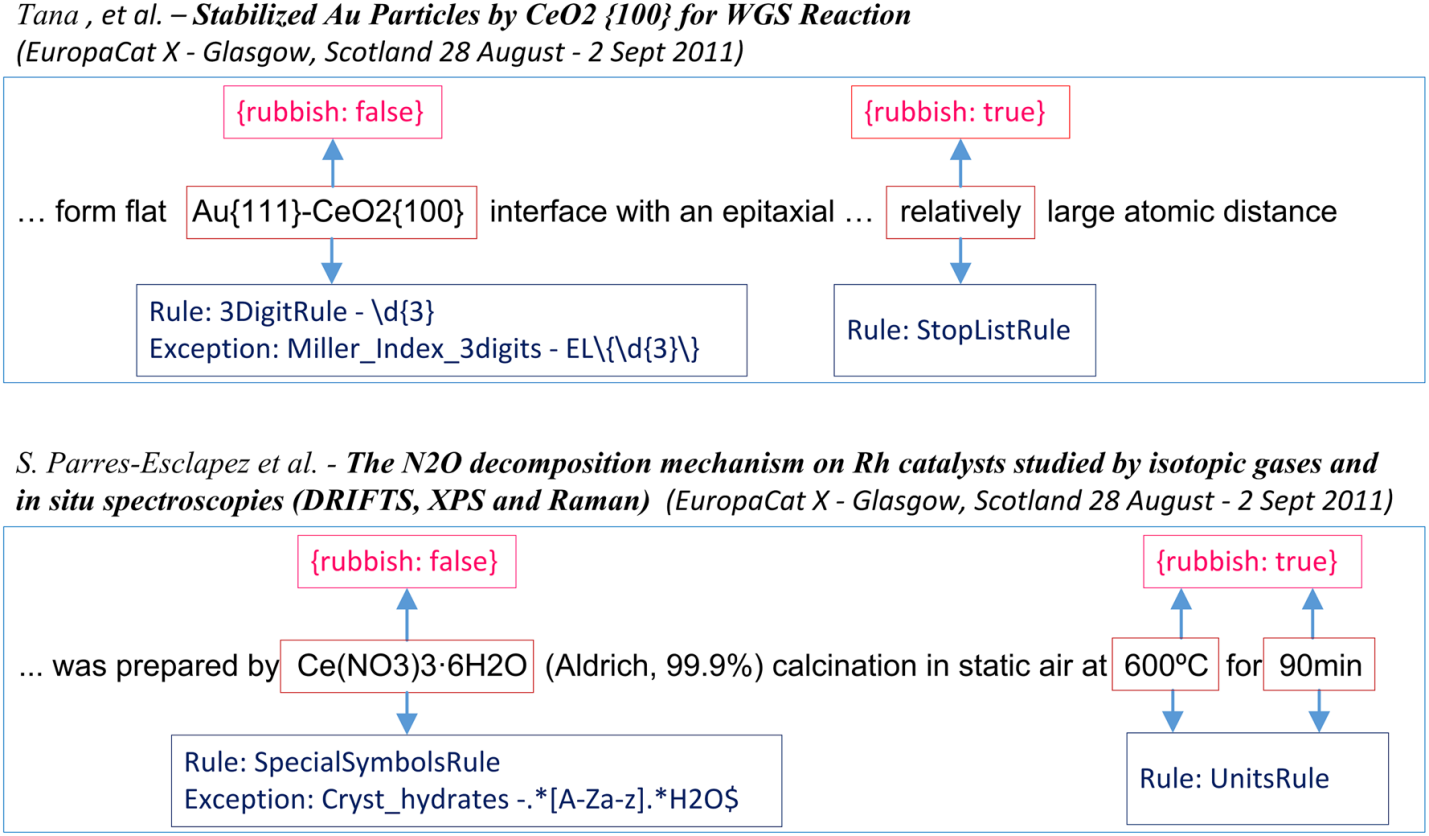
Examples of strict filtering tagging

The following examples may be given to illustrate the decision making process of defining a token as “valid” or “rubbish” (Fig. 5).

#### Summary of preprocessing stage

The final result of the text preprocessing stage is the marked and structured text with tagged tokens. These tags are used then by various rules for term-like phrase selection. As there is no need for all the tags from OSCAR4 and Penn Treebank Tag Set, only a few of them are used in term-like phrases retrieval procedure. The consolidated list of all tags is used, which may be assigned to tokens at different steps of the text preprocessing stage, as specified in the Table 4.

**Table 4:** The consolidated list of all tags assigned to tokens at different steps of the text preprocessing stage

Group of tags	Tag	Explanation	Strict filtering	Morphological pattern
*POS*				
	JJ	Adjective		Yes (n-grams n > 1)
	JJR	Adjective, comparative		Yes (n-grams n > 1)
	VBG	Verb, gerund or present participle		Yes (n-grams n ≥ 1)
	VBD	Verb, past tense includes the conditional form of the verb to be		Yes (n-grams n > 1)
	VBN	Verb, past participle		Yes (n-grams n > 1)
	NNP	Proper Noun, singular		Yes (n-grams n > 1)
	NN	Noun, singular or mass		Yes (n-grams n ≥ 1)
	NNPS	Proper Noun, plural		Yes (n-grams n ≥ 1)
	NNS	Noun, plural		Yes (n-grams n ≥ 1)
	IN	Preposition or subordinating conjunction		Yes (n-grams n > 1)
	DT	Determiner		Yes (n-grams n > 1)
	RB	Adverb		Yes (n-grams n > 2)
	RBS	Adverb, superlative		Yes (n-grams n > 2)
	FW	Foreign word		Yes (n-grams n > 1)
*OSCAR*				
	CM	Chemical matter	Yes	Yes (all n-grams)
	ONT	Ontological term	Yes	Yes (all n-grams)
*Own tags*				
	COMP	Chemical composition		Yes (all n-grams)
	rubbish	Token for which strict filtering to be applied	Yes	Yes (all n-grams)
	GCST	General Chemistry Scientific Term		Yes (all n-grams)

It is also indicated whether a tag is used in strict filtering or in term-like phrases retrieval procedure with help of POS-based rules

**Fig. 6:**
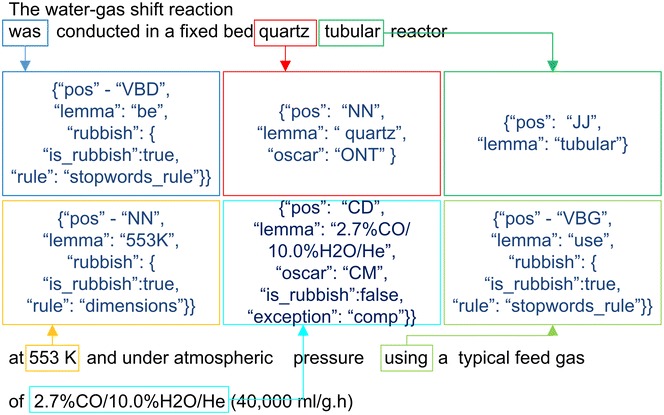
An illustration of tags assignment to different tokens

As an illustration of tag assignment the following example may be given. Figure 6 shows an example sentence where a few tokens have been tagged. For instance, there are the following different tags used in the example for token «*2.7* *%CO/10.0* *%H2O/He*» – (**pos** = “CD”; **lemma** = “2.7 %CO/10.0 %H_2_O/He”; **oscar** = “CM”; **rubbish** = “false”, **exception** = “comp”). Every token has at least two tags—«**pos**» (it holds the part-of-speech information) and «**lemma**» (it corresponds to the lemma of a token). In addition some tokens related to chemistry (indicating chemical substances, formulas, reactions and etc.) have a tag «**oscar**» taking the values of “CM” or “ONT”. Last but not least is the tag «**rubbish**» (“true” or “false”) marking tokens for which strict filtering is to be applied.

### N-grams spectrum retrieval procedure

As it is defined earlier within our study, the term «n-gram at length *n*» connotes a sequence or string of *n* consecutive tokens situated within the same sentence with omission of useless tokens (at the moment only definite/indefinite articles). N-gram set is obtained by moving a window of *n* tokens length through an entire sentence. This moving is performed token by token. This process is to be repeated for all sentences for a set of all texts: $T = \left\{ {T_{1} , T_{2} , \ldots , T_{m} } \right\}$.

For a set of texts, each n-gram may be characterized by textual frequency of n-gram occurrence $f_{T} \left( {T_{i} } \right)$—total number of n-gram occurrences within a text $T_{i}$ and by absolute frequency of occurrence $f_{A} = \mathop \sum \limits_{i} f_{A} \left( {T_{i} } \right)$—total number of n-gram occurrences. As a result each n-gram may be described by a vector ${\mathbf{F}}\left( T \right) = \left\{ {f_{T} \left( {T_{1} } \right), f_{T} \left( {T_{2} } \right), \ldots , f_{T} \left( {T_{m} } \right)} \right\}$ within a set of texts enabling us to develop the additional procedures for n-gram filtering and text information analysis.

The full n-gram data set is redundant and it creates difficulties for analysis. For specific purposes different filtration procedures are to be applied. For instance, threshold filtering based on the values of ${ \hbox{max} } f_{A} = { \hbox{max} } \mathop \sum \nolimits_{i} f_{T} \left( {T_{i} } \right)$ and ${ \hbox{max} } f_{T} \left( {T_{i} } \right)$ may be used.

### Module of terminology spectrum building

The final stage of the analysis is to distinguish among the scores of n-grams such as the term-like phrases, general chemistry scientific terms, names of chemical entities and useless n-grams. The calculation of textual and absolute frequencies of terms occurrence finishes the terminology spectrum building.

To select term-like n-grams the sets of accept and reject rules are applied. They are all based on using token tags assigned at previous steps and developed dictionaries (Table 1). The intention of each set of rules is to determine whether an n-gram of defined length is a term-like phrase or not by analyzing its structure. All rules are applied in a consecutive manner. If an n-gram conforms to an accept or reject rule in the rule sequence, the procedure will be stopped with declaring the n-gram as either a non-term-like or a term-like phrase, probably having a special meaning (e.g. general chemistry scientific term or chemical entity). If no rule is applicable, the n-gram will be considered a term-like phrase too. There are a few general rules that can be used for analysis of n-grams of any length. There are also tailored sets of rules for 1-grams (Table 5), 2-grams (Table 6) and for long (n > 2)-grams (Table 7).

**Table 5:** Accept and reject rules succession for unigrams (1-grams)

Description	Examples
***GeneralChemTermRule (accept rule)*** True if a 1-gram is a general chemistry scientific term	
***StrictFilteringTagRule (reject rule)*** True if a 1-gram consists of a token with the strict filtering tag «rubbish:true»	
***ShortTokensRule (reject rule)*** True if a 1-gram consists of a short token of length less than three characters *This rule is to exclude noise existing in documents such as axes labels and so on*	
***UnitsRule (reject rule)*** True if a 1-gram contains a string being a measurement unit from the dictionary (Table 1)	
***ChemUnigramRule (accept rule)*** True if a 1-gram is tagged by any OSCAR tag and by one of the following POS tags: FW, NNP, or tagged by tag COMP. *Selected unigrams are assumed and marked to have a chemical sense.* **Term-like:** barium, phenanthrene, pentanol, xanes	
***GeneralEnglishDictRule (reject rule)*** True, if a 1-gram is in the General English Dictionary (Table 1)	*Filtered:* topography, paint, plateau, pool, searching, file, addenda, improvement, theme … Term-like: hydrocalcite, acetylacetone, cracking, ageing
***UnigramPOSRule (reject rule)*** True, if a 1-gram is not a noun or a gerund. Term-like 1-gram must be tagged with the following POS tags: VBG, NN, NNPS, NNS	*Filtered:* schematized, suddenly, skeletal, behind Term-like: ethylene, hydrocalcite, leaching, 12n-decylhexadecanamide, sulfamethoxazole, anchoring
***UnigramAddRules (reject rules)*** Set of regular expressions to filter unigrams denoting various ions, signs, captions and etc.	*Filtered:* M(O2), GA15.6, PW91, V2.1, G(D), TI(V), PD(I), PT0, P(X), BA2+, CE(3+), cm3, CH3, AA, Cu2+, Mo6+, Et-CP, GC–MS, Zn-Al

**Table 6:** Reject and accept rules consecution for bigrams (2-grams)

Description	Examples
***GeneralChemTermRule (accept rule) *** *(the same rule as for 1-grams)*	
***StrictFilteringTagRule (reject rule)*** *(the same rule as for 1*-*grams)*	
***ShortTokensRule (reject rule)*** True if a 2-gram consists of only short tokens <3 characters	
***IdenticalTokensRule (reject*** ***rule)*** True if a 2-gram contains at least two identical tokens	
***UnitsRule (reject rule)*** True if any token in a 2-gram ends with measurement unit string from the dictionary (Table 1) *It should be noted that measurement unit may be consisted of several tokens, for example, the “g/h” consists of three tokens [“g”, “/”, “h”]*	PPM C7H14, 70ML MIN-1, CM3MIN-1 H2, MIN-1 FLOW, H-1 GAS, PPM N2O/AR, ML G-1MIN-1, MOL-1 HYDROLYSIS, PPM NOX/5%O2/N2
***BiGramPOSRule (accept rule with exception)*** True, if the fist token is tagged with one of the following POS tags: JJ, JJR, FW, VBG, VBD, VBN, NN, NNP, NNPS, NNS and the second token is tagged with one of: FW, VBG, NN, NNP, NNPS, NNS Exception—the following combinations are not allowed: «VBG,VBG» , «VBG,FW» , «NNP, FW»	*Term-like:* Andronov bifurcation, Na2CO3 impregnation, nickel catalyst; supported MgO, anchored lysine, stirred glass; carbonaceous particle, temperature-programmed adsorption, Fischer–Tropsch catalyst; in situ EXAF, UV–VIS spectroscopy, Raman spectroscopy *Filtered due to exception:* involving reforming, reforming minimizing, using in, Shimada etc

**Table 7:** Reject and accept rules consecution for n-grams (n ≥ 3)

Description	Examples
***GeneralChemTermRule (accept rule) *** *(the same rule as for 2-grams)*	
***StrictFilteringTagRule (reject trule)*** *(the same rule as for 2*-*grams)*	
***ShortTokensRule (reject rule)*** *(the same rule as for 2*-*grams)*	
***IdenticalTokensRule (reject rule)*** *(the same rule as for 2*-*grams)*	
***UnitsRule (reject rule)*** *(the same rule as for 2*-*grams)*	
***ManyGramPOSRule (accept rule with exception)*** True, if the **fist token** must be tagged with one of the following POS tags (noun, gerund, adjective, adverb or participle): NN, NNP, VBG, VBD, VBN, JJ, JJR, RB, RBS, FW **and** the **middle** in any position token (+ preposition or determiner): NN, NNP, VBG, VBD, VBN, JJ, JJR, RB, RBS, FW + **IN**, **DT** **and** the **last** token: VBG,NN,NNP,NNPS,NNS (gerund or noun) Exception—the following combinations are not allowed (describing phrases which looks like to be torn from their context): «first token:VBG ->second token NN or IN» , «first token:VBN ->second token NN or:JJ»	*Term-like:* X-ray fluorescence spectrometer; Brønsted basic site; Pd(110) surface oscillation; doping CsPW with platinum; catalyzed N2O decomposition; crystalline phase transition; catalyzed oxidation of NO; complete photoreduction of Pd(II); propagating thermosynthesis; reforming of the biomass; drying inside the microscope column *Filtered due to exception:*used during steam reforming; catalyzed by metalloporphyrin; investigated by XRD; using atomic absorption

**Fig. 7:**
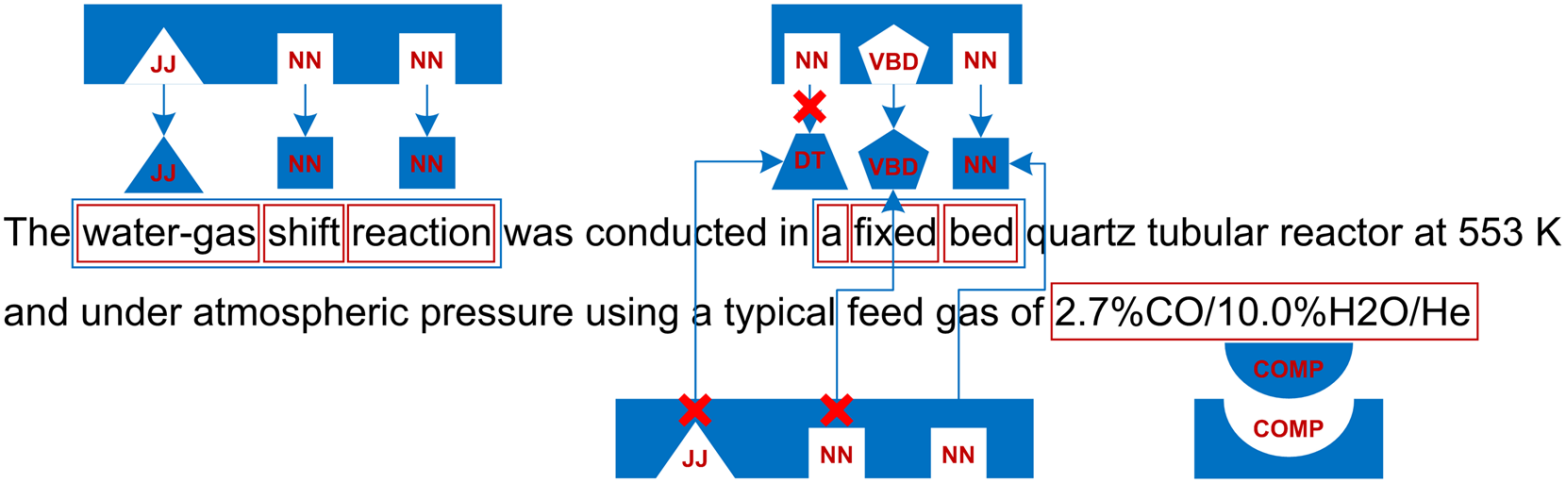
An illustration of term-like phrases retrieval procedure with POS based accept rules

The following examples may be given to illustrate the decision making process whether an n-gram may be considered a term-like phrases or not (Fig. 7).

The next step in the terminology analysis stage is the tagging of term-like phrases to describe their roles as entities having a special meaning. There are the following tags at the moment: «term-like phrase», «general chemistry term», and «chemical entity». The final step is the additional filtration procedure aimed to reduce the number of term-like phrases performed by removing short term-like phrases which are parts of n-grams with more length. The criterion of filter application is equality of the absolute frequencies of occurrence for short and long n-grams.

## Results and discussion

**Fig. 8:**
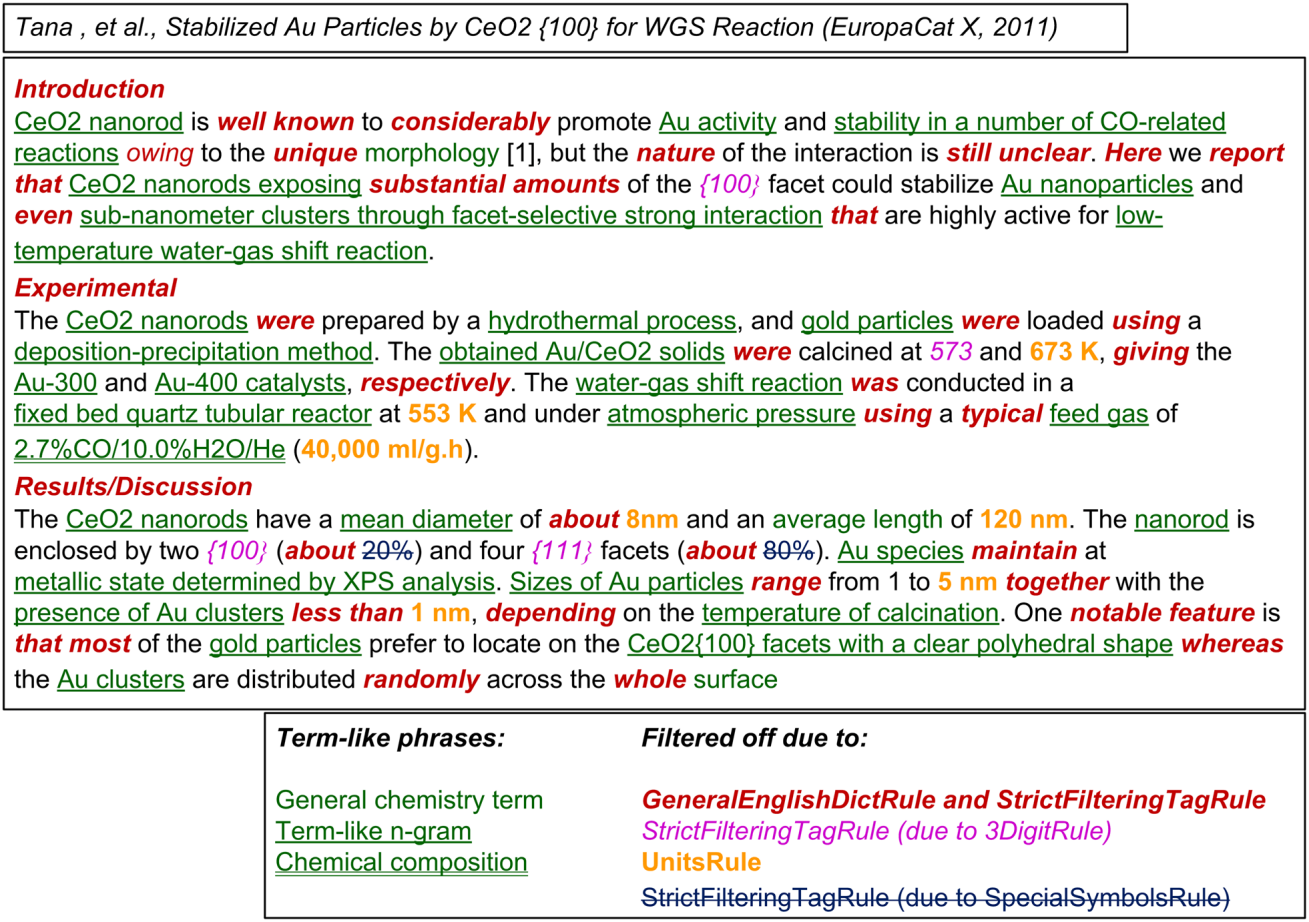
An example of terminology analysis results (with some term-like and filtered-off n-grams *highlighted*)

An example of automatic term-like phrases retrieval is shown in Fig. 8 with some term-like and filtered-off n-grams highlighted. For the filtered-off n-grams the reject rules used are given as well. For the detailed results of terminology analysis for one preselected Congress abstract see the Additional file 1.

To understand the overall performance of term-like phrases retrieval routine the full set of text abstracts belonging to 5 EuropaCat events were processed. Obtained data were statistically analyzed (see Table 8). It may be seen that term-like phrases retrieval procedure reduces the total number of all available n-grams to a range of 1÷3 %, which depends on the n-gram length n.

**Table 8:** Consolidated table of experimental results on terminology analysis of EuropaCat abstracts set

n	N—total number of n-grams	N_TL_—total number of term-like phrases (% of N)	N_GS_—total number of general scientific terms (% of N_TL_)	N_COMP_—total number of phrases with tag «COMP» (% of N_TL_)	N_CM_—total number of phrases with OSCAR tag «CM» (% of N_TL_)
1	~5.15 × 10^6^	68,811 (~1.3 %)	574 (0.8 %)	8776 (12.7 %)	40,354 (58.6 %)
2	~4.94 × 10^6^	135,002 (~2.7 %)	11,263 (8.3 %)	5199 (3.9 %)	52,641 (38.9 %)
3	~4.74 × 10^6^	130,706 (~2.8 %)	1031 (0.8 %)	5194 (4 %)	64,101 (49.0 %)
4	~4.54 × 10^6^	118,893 (~2.6 %)	41 (0.03 %)	4064 (3.4 %)	56,047 (47.1 %)
5	~4.35 × 10^6^	94,546 (~2.2 %)	5 (0.005 %)	3390 (3.6 %)	43,550 (46.0 %)
6	~4.16 × 10^6^	58,775 (~1.4 %)	–	2469 (4.2 %)	29,992 (51.0 %)
7	~3.97 × 10^6^	46,224 (~1.2 %)	–	2403 (5.2 %)	26,030 (56.3 %)

Number of texts: 6387; total amount of tokens: 5,148,124 (EuropaCat 2013, 2011, 2009, 2007, 2005)

Table 8 demonstrates that the maximum absolute amount of term-like n-grams corresponds to the value of n = 2 (bigrams), which is in good accordance with the well-known fact of the average term length in scientific texts. On the other hand, term indexes are often limited to the n-grams lengths n = 1, 2, 3. The limit n = 3 looks good enough for general science vocabulary (see N_GS_ value from Table 8—a number of general scientific terms found), but it is not sufficient for a specialized thesaurus (e.g. for catalysis). The numbers of term-like n-grams with COMP tag are also large for different n including n > 3. Summarizing, it should be said that long-length terms retrieval is the distinctive feature of the suggested approach.

It is also seen from Table 8 that near half of total amount of 1-grams have an OSCAR tag “CM”. It should be noted also that if a plausible term-like phrase has just one token with OSCAR tag, it will be considered also as having the same tag by the system. It may explain the close values (in percentages) for phrases with different length.

**Fig. 9:**
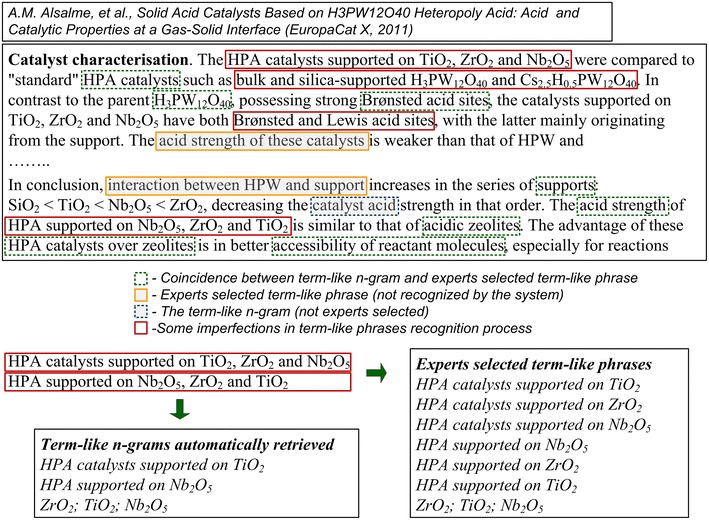
An example of terminology analysis results (with some automatically retrieved and expert selected term-like phrases)

To assess the overall effectiveness of the term-like phrases retrieval procedure it seems necessary to quantitatively answer the questions about what precision and recall values are possible to be achieved. To do that a preliminary study on comparison between automatically and manually selected term-like phrases was performed with the help of two professional chemical scientists who picked out the term-like phrases from a limited set of a few arbitrarily selected documents. To include a phrase in the list of term-like phrases a consent among both experts was required. It should be noted here that experts were not required to follow the same procedure of moving a window of *n* tokens length on an entire sentence used by n-grams isolation. Moreover, experts took into account and analyzed the information put into some simple grammatical structures, which are typical for scientific texts, such as structures with enumeration and so on. It leads to additional differences between the sets of expert and automatically selected term-like phrases (for an example see Fig. 9).

The data obtained through expert terminological analysis were compared with the automatically retrieved terms. The precision (P), recall (R) and F-measure values were calculated. In the paper, the precision [21] indicates a fraction of automatically retrieved term-like phrases which coincide with expert selected ones. Recall is a fraction of an expert’s selected term-like phrases that are retrieved by the system.

$$\begin{aligned} P = \frac{{{\text{Number}}\,{\text{of}}\,{\text{coincidences}}}}{{{\text{Number}}\,{\text{of}}\,{\text{term-like}}\,{\text{phrases}}}};\quad R = \frac{{{\text{Number}}\,{\text{of}}\,{\text{coincidences}}}}{{{\text{Number}}\,{\text{of}}\,{\text{terms}}\,{\text{retrived}}\,{\text{by}}\,{\text{experts}}}} \hfill \\ \left( {{\text{Number}}\,{\text{of}}\,{\text{coincidences}}} \right) = {\text{Number}}\,{\text{of}}\,\left\{ {\left( \begin{aligned} {\text{Term-like}}\,{\text{phrases}} \hfill \\ {\text{retrived}}\,{\text{by}}\,{\text{experts}} \hfill \\ \end{aligned} \right) \cap \left( \begin{aligned} {\text{Term-like}}\,{\text{phrases}} \hfill \\ {\text{retrived}}\,{\text{by}}\,{\text{the}}\,{\text{system}} \hfill \\ \end{aligned} \right)} \right\} \hfill \\ \end{aligned}$$

Both precision and recall therefore may be used as a measure of term-like phrases retrieval process relevance and efficiency. In simple terms, high precision values mean that substantially more term-like phrases are selected than the number of erroneous phrases, while high recall values mean that the most term-like phrases are selected from the text.

Very often these two measures (P and R) are used together to calculate a single value named as F_1_-measure [22] to provide an overall performance system characteristic. F_1_-measure is a harmonic mean of P and R, where F_1_ can reach 1 as its best and 0 as its worst values:

$$F_{1} = 2PR/\left( {P + R} \right)$$

The results on the number of expert selected and automatically retrieved term-like phrases, number of coincidences and calculated P, R and F_1_ values are represented in Table 9. For the detailed results of terminology analysis for one preselected text, see the Additional file 1.

**Table 9:** Precision, Recall and F-measure estimated from the data obtained for 5 arbitrarily selected texts

Text no.	Number of terms retrieved by 2 experts	Number of term-like phrases retrieved by the system	Number of coincidences	Precision	Recall	F_1_-measure
No. 1	164	221	135	0.61	0.82	0.70
No. 2	155	174	96	0.55	0.62	0.58
No. 3	170	172	113	0.66	0.66	0.66
No. 4	68	119	40	0.34	0.59	0.43
No. 5	125	215	106	0.50	0.85	0.63
***P, R and F values calculated for the entire 5 texts set:*** *Unique expert term*-*like phrases*—*655* *Term*-*like n*-*grams*—*872* *Coincidences*—*466*				***0.53***	***0.71***	***0.61***

No. 1—Design, synthesis and catalysis of recoverable catalysts assembled in emulsion and…, C. Li et al. (2005)

No. 2—Understanding reaction pathways on model catalyst surfaces, F. Gao et al. (2007)

No. 3—Solid acid catalysts Based on H_3_PW_12_O_40_ Heteropoly Acid: Acid and Catalytic Pr…, A.M. Alsalme et al. (2011)

No. 4—Advantages of using TOF–SIMS method in surface studies of heterogeneous…, M.I Szynkowska et al. (2005)

No. 5—ECS-Materials: synthesis and characterization of a new class of crystalline…, G. Bellussi et al. (2007)

It may be concluded therefore that further improvements can be made with term-like phrase retrieval efficiency by bringing into consideration the knowledge of typical grammatical structures used in scientific texts [12, 23] as well as numeric values of both textual and absolute frequencies of n-gram occurrences.

It is also seen that the first version of the terminology analysis system delivers sufficiently high values for precision and recall achievable in term-like phrases retrieval process. Some comparison can be made with P = 0.34÷0.40, R = 0.11÷0.14, F_1_ = 0.17÷0.20 values reported [6] by such well-known keyphrases retrieval systems as Wingnus, Sztergak, KP-Mminer, although such disparity does not look consistent enough to be credible due to different goals of the systems (term-like phrases vs. keyphrases retrieval) being brought into comparison.

## Conclusions

As mentioned in the introduction, scientific publications are still the most important sources of scientific knowledge and new methods aimed to retrieve meaningful information from natural language documents are particularly welcome today. The structural foundation of any such publication is widely accepted terms and term-like phrases conveying useful facts and shades of meaning of a document content.

The present study is aimed to develop, test and assess the methodology of automated extraction of full terminology spectrum from natural language chemical pdf documents, with retrieving as much term-like phrases as is possible. Term-like phrases are defined as one or more consecutive words and/or alphanumeric string combinations, which convey specific scientific meaning with unchanged spelling and context as in a real text. Terminology spectrum of a natural language publication is defined as an indexed list of tagged entities: recognized general science notions, terms linked to existing thesauri, names of chemical substances/reactions and term-like phrases. The retrieval routine is based on n-gram text analysis with sequential application of complex accept and reject rules. The main distinctive feature of the suggested approach is in picking out all parsable term-like phrases, not just selecting a limited set of keyphrases meeting any predefined criteria. The next step is to build an extensive term index of a text collection. The developed approach neither takes into account semantic similarity nor differentiates between similar term-like phrases (distinct evaluation metrics may be employed to do it at the later stages). The approach which includes a number of sequentially running procedures appears to show good results in terminology spectrum retrieval as compared with well-known keyphrases retrieval systems [6]. The term-like phrase parsing efficiency is quantified with precision (P = 0.53), recall (R = 0.71) and F_1_-measure (F_1_ = 0.61) values calculated from a limited set of documents manually processed by professional chemical scientists.

Terminology spectrum retrieval may be used to perform various types of text analysis across document collections. We believe that this sort of the terminology spectrum may be successfully employed for text information retrieval and for reference database development. For example, it may be used to develop thesauri, to analyze research trends in subject fields of research by registering changes in terminology, to derive inference rules in order to understand particular text content, to look for the similarity between documents by comparing their terminology spectrum within an appropriate vector space, to develop methods to automatically map document to a reference database field.

For instance, if a set $T = \left\{ {T_{1} , T_{2} , \ldots , T_{m} } \right\}$. contains a collection of texts from different time periods (in our research, several different events from the EuropaCat research conference were used), the analysis of textual and absolute frequencies of occurrence will allow to follow up the “life cycle” of each term-like phrase on the quantitative level (term usage increasing, decreasing and so on). That gives a unique capability to find out research trends and new concepts in the subject field by registering changes in terminology usage in the most rapidly developing areas of research. Moreover, similar dynamics of change over time for different terms often indicates the existence of an associative linkage between them (e.g. between a new process and developed catalyst or methodology). Indicator words or phrases such as “for the first time”, “unique”, “distinctive feature” and so on may also be used in order to detect things like new recipes or catalyst composition for the explored process.

Usage of terminology spectrum for information retrieval will be the subject of our subsequent publications.

## Additional files

10.1186/s13321-016-0136-4 The detailed example of PDF transformation with terminology analysis performed by experts and by automatic analysis.
10.1186/s13321-016-0136-4 OSCAR4 tokenizer modification.
10.1186/s13321-016-0136-4 List of excluded words from general English Corncob- Lowercase list.
10.1186/s13321-016-0136-4 List of stop words used.
10.1186/s13321-016-0136-4 List of stable isotopes.
10.1186/s13321-016-0136-4. List of chemical element symbols.
10.1186/s13321-016-0136-4 List of measurement units.
