# Supplementary material for: Terminology spectrum analysis of natural-language chemical documents: term-like phrases retrieval routine
Source: J Cheminform. 2016 Apr 29;8:22. doi: 10.1186/s13321-016-0136-4 (PMC4850643; doi:10.1186/s13321-016-0136-4)
Supplement: Supplementary file 1 — 10.1186/s13321-016-0136-4 The detailed example of PDF transformation with terminology analysis performed by experts and by automatic analysis. [file 13321_2016_136_MOESM1_ESM.pdf]

**Additional file 1**
**The detailed example of PDF tramsformation with terminoly analysis performed by experts and by automatical analysis**

## Content


Original pdf file ........ 2
Converted pdf file ........ 4
The list of experts selected term-like phrases ........ 5
The list of term-like n-grams ........ 6
The list of term-liike n-grams recognized as the general chemistry terms ........ 7
The list of term-like n-grams with tag «COMP» ........ 8
The list of term-like n-grams with OSCAR tags ........ 8
The list of filtered off n-grams ........ 9
Comparison between automatically selected term-like n-grams and expert selected terms ........ 35

## Original pdf file

# Solid Acid Catalysts Based on $H_3PW_{12}O_{40}$ Heteropoly Acid: Acid and Catalytic Properties at a Gas-Solid Interface

A.M. Alsalme, P.V. Wiper, Y.Z. Khimyak, E.F. Kozhevnikova, I.V. Kozhevnikov*
*Department of Chemistry, University of Liverpool, Liverpool L69 7ZD, UK*
*kozhev@liverpool.ac.uk

### *Introduction*

Heterogeneous acid catalysis by heteropoly acids (HPAs) has attracted much interest because of its potential to generate economic rewards and green benefits [1]. In recent years, there has been considerable research activity, focusing on acidic composites comprising tungsten HPAs and $Nb_2O_5$, $ZrO_2$ and $TiO_2$ as oxide matrixes. The aim of this work is to study the acidity and catalytic activity of such composites at a gas-solid interface. The catalysts under study comprise the strongest Keggin HPA, $H_3PW_{12}O_{40}$ (HPW) supported at sub-monolayer coverage on $Nb_2O_5$, $ZrO_2$ and $TiO_2$.

### *Experimental*

The catalysts comprising HPW (15 wt%) supported onto $SiO_2$, $Nb_2O_5$, $ZrO_2$ and $TiO_2$ were prepared by wet impregnation of these oxides with HPW from aqueous solution and characterised by BET, TGA, XRD, FTIR and $^{31}P\{^1H\}$ MAS NMR [2]. The acidity of catalysts was assessed by DRIFTS of adsorbed pyridine and $NH_3$ adsorption calorimetry [2]. The catalysts were tested in isopropanol dehydration [2] and $\alpha$-pinene isomerisation [3] in the gas phase in a fixed-bed reactor with on-line GC analysis.

### *Results/Discussion*

**Catalyst characterisation.** The HPA catalysts supported on $TiO_2$, $ZrO_2$ and $Nb_2O_5$ were compared to "standard" HPA catalysts such as bulk and silica-supported $H_3PW_{12}O_{40}$ and $Cs_{2.5}H_{0.5}PW_{12}O_{40}$. In contrast to the parent $H_3PW_{12}O_{40}$, possessing strong Brønsted acid sites, the catalysts supported on $TiO_2$, $ZrO_2$ and $Nb_2O_5$ have both Brønsted and Lewis acid sites, with the latter mainly originating from the support. The acid strength of these catalysts is weaker than that of HPW and $Cs_{2.5}H_{0.5}PW_{12}O_{40}$. Ammonia adsorption calorimetry, $^{31}P$ MAS NMR and FTIR indicate increasing interaction between support and HPA in the following order of supports: $SiO_2 < TiO_2 < Nb_2O_5 < ZrO_2$, causing partial decomposition of HPA and decreasing the catalyst acid strength in that order [2].

**Isopropanol dehydration.** The catalytic activity (turnover frequency, TOF) in the gas-phase isopropanol dehydration was found to decrease in line with the catalyst acid strength as determined by $NH_3$ adsorption calorimetry. There is a good linear relationship between log

(TOF) and the initial enthalpy of ammonia adsorption for these catalysts, $\Delta H_i$ (Figure 1). This shows that isopropanol dehydration and $NH_3$ adsorption calorimetry give consistent results regarding the acidity of HPA catalysts.

**α-Pinene isomerisation.** The isomerisation of α-pinene was studied in the gas phase at 200°C and ambient pressure [3]. The reaction yielded camphene as the main product in a mixture with monoterpene by-products such as limonene, terpinolenes, terpinenes, β-pinene, p-cymene and others. The HPA catalysts with strong Brønsted acid sites exhibited high initial activities, but suffered from catalyst deactivation, resulting in low camphene yields. Conversely, the HPA catalysts supported on $Nb_2O_5$, $ZrO_2$ and $TiO_2$, although weaker acids, showed more stable performance in α-pinene isomerisation. The 15% HPW/$TiO_2$ catalyst gave a camphene yield of 51% and a total camphene and limonene yield of 58%, which is similar to the commercial liquid-phase batch process, with the advantage of continuous operation.

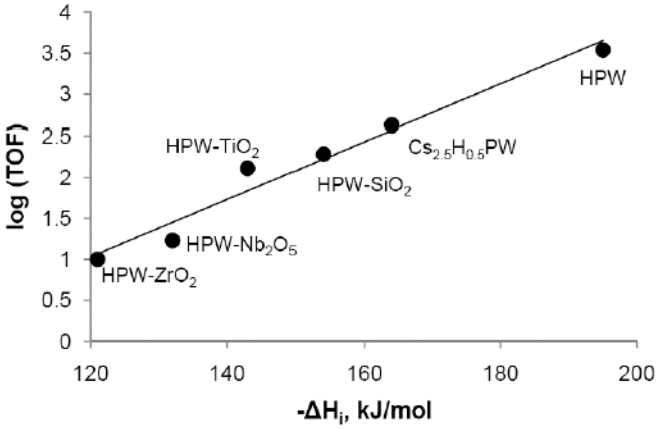


Figure 1. Relationship between log (TOF) and $\Delta H_i$ for bulk and supported HPW catalysts.

In conclusion, interaction between HPW and support increases in the series of supports: $SiO_2 < TiO_2 < Nb_2O_5 < ZrO_2$, decreasing the catalyst acid strength in that order. The acid strength of HPA supported on $Nb_2O_5$, $ZrO_2$ and $TiO_2$ is similar to that of acidic zeolites. The advantage of these HPA catalysts over zeolites is in better accessibility of reactant molecules, especially for reactions involving large organic molecules such as terpenes. Development of thermally stable HPA composites possessing stronger acid sites remains a challenge for further research.
Support from the EPSRC (research grants EP/E039847 and EP/F014686/1) is acknowledged.

***References***

1. I.V. Kozhevnikov, *Catalysis by Polyoxometalates,* John Wiley & Sons, Chichester, 2002.
2. A.M. Alsalme, P.V. Wiper, Y.Z. Khimyak, E.F. Kozhevnikova, I.V. Kozhevnikov, *J. Catal.* **276,** 181 (2010).
3. A.M. Alsalme, E.F. Kozhevnikova, I.V. Kozhevnikov, *Appl. Catal. A* **390,** 219 (2010).

# Converted pdf file

{
"_id" : ObjectId("55a8971460b28d8ed1c1d3de"),
"year" : 2011,
"filePath" : "Euro/2011/Orals/Catalyst Characterisation/CO34 - 1690988.pdf",
"collectionId" : "EuropaCat_2011",
"authors" : "A.M. Alsalme, P.V. Wiper, Y.Z. Khimyak, E.F. Kozhevnikova, I.V. Kozhevnikov",
"affilations" : "Department of Chemistry, University of Liverpool, Liverpool L69 7ZD, UK",
"title" : "Solid Acid Catalysts Based on H3PW12O40 Heteropoly Acid: Acid  and Catalytic Properties at a Gas-Solid Interface",
"content" :
"Introduction

Heterogeneous acid catalysis by heteropoly acids (HPAs) has attracted much interest because of its potential to generate economic rewards and green benefits [1]. In recent years, there has been considerable research activity, focusing on acidic composites comprising tungsten HPAs and Nb2O5, ZrO2 and TiO2 as oxide matrixes. The aim of this work is to study the acidity and catalytic activity of such composites at a gas-solid interface. The catalysts under study comprise the strongest Keggin HPA, H3PW12O40 (HPW) supported at sub-monolayer coverage on Nb2O5, ZrO2 and TiO2.

Experimental

The catalysts comprising HPW (15 wt%) supported onto SiO2, Nb2O5, ZrO2 and TiO2 were prepared by wet impregnation of these oxides with HPW from aqueous solution and characterised by BET, TGA, XRD, FTIR and 31P{1H} MAS NMR [2]. The acidity of catalysts was assessed by DRIFTS of adsorbed pyridine and NH3 adsorption calorimetry [2]. The catalysts were tested in isopropanol dehydration [2] and α-pinene isomerisation [3] in the gas phase in a fixed-bed reactor with on-line GC analysis.

Results/Discussion

Catalyst characterisation. The HPA catalysts supported on TiO2, ZrO2 and Nb2O5 were compared to \"standard” HPA catalysts such as bulk and silica-supported H3PW12O40 and Cs2.5H0.5PW12O40. In contrast to the parent H3PW12O40, possessing strong BrØnsted acid sites, the catalysts supported on TiO2, ZrO2 and Nb2O5 have both BrØnsted and Lewis acid sites, with the latter mainly originating from the support. The acid strength of these catalysts is weaker than that of HPW and Cs2.5H0.5PW12O40. Ammonia adsorption calorimetry, 31P MAS NMR and FTIR indicate increasing interaction between support and HPA in the following order of supports: SiO2 < TiO2 < Nb2O5 < ZrO2, causing partial decomposition of HPA and decreasing the catalyst acid strength in that order [2].

Isopropanol dehydration. The catalytic activity (turnover frequency, TOF) in the gas-phase isopropanol dehydration was found to decrease in line with the catalyst acid strength as determined by NH3 adsorption calorimetry. There is a good linear relationship between log (TOF) and the initial enthalpy of ammonia adsorption for these catalysts, ΔHi (Figure 1). This shows that isopropanol dehydration and NH3 adsorption calorimetry give consistent results regarding the acidity of HPA catalysts.

α-Pinene isomerisation. The isomerisation of α-pinene was studied in the gas phase at 200oC and ambient pressure [3]. The reaction yielded camphene as the main product in a mixture with monoterpene by-products such as limonene, terpinolenes, terpinenes, β-pinene, p-cymene and others. The HPA catalysts with strong BrØnsted acid sites exhibited high initial activities, but suffered from catalyst deactivation, resulting in low camphene yields. Conversely, the HPA catalysts supported on Nb2O5, ZrO2 and TiO2, although weaker acids, showed more stable performance in α-pinene isomerisation. The 15% HPW/TiO2 catalyst gave a camphene yield of 51% and a total camphene and limonene yield of 58%, which is similar to the commercial liquid-phase batch process, with the advantage of continuous operation.

Figure 1. Relationship between log (TOF) and ΔHi for bulk and supported HPW catalysts.

In conclusion, interaction between HPW and support increases in the series of supports: SiO2 < TiO2 < Nb2O5 < ZrO2, decreasing the catalyst acid strength in that order. The acid strength of HPA supported on Nb2O5, ZrO2 and TiO2 is similar to that of acidic zeolites. The advantage of these HPA catalysts over zeolites is in better accessibility of reactant molecules, especially for reactions involving large organic molecules such as terpenes. Development of thermally stable HPA composites possessing stronger acid sites remains a challenge for further research.

Support from the EPSRC (research grants EP/E039847 and EP/F014686/1) is acknowledged."

"references" : "References 1. I.V. Kozhevnikov, Catalysis by Polyoxometalates, John Wiley & Sons, Chichester, 2002. 2. A.M. Alsalme, P.V. Wiper, Y.Z. Khimyak, E.F. Kozhevnikova, I.V. Kozhevnikov, J.  Catal. 276, 181 (2010). 3. A.M. Alsalme, E.F. Kozhevnikova, I.V. Kozhevnikov, Appl. Catal. A 390, 219 (2010).",
}

# The list of experts selected term-like phrases

15%HPW/TIO2
15%HPW/TiO2 catalyst
31P MAS NMR
31P{1H} MAS NMR
accessibility of reactant molecule
acid
acid catalysis
acid property
acid site
acid strength
acid strength of acidic zeolite
acid strength of catalysts
acid strength of Cs2.5H0.5PW12O40
acid strength of HPA supported on Nb2O5
acid strength of HPA supported on TiO2
acid strength of HPA supported on ZrO2
acid strength of HPW
acidic composite
acidic composites comprising tungsten HPA
acidic zeolite
acidity
acidity of catalyst
acidity of composite
acidity of HPA catalyst
activity
adsorbed pyridine
adsorption
adsorption calorimetry
ambient pressure
ammonia
ammonia adsorption calorimetry
aqueous solution
BET
better accessibility of reactant molecules
brønsted acid
brønsted acid site
bulk Cs2.5H0.5PW12O40
bulk H3PW12O40
camphene
camphene as a main product
camphene yield
catalyst
catalyst acid strength
catalyst characterisation
catalyst comprising HPW supported onto Nb2O5
catalyst comprising HPW supported onto SiO2
catalyst comprising HPW supported onto ZrO2
catalyst comprising HPW supported ontoTiO2
catalyst deactivation
catalyst supported
catalyst supported on Nb2O5
catalyst supported on TiO2
catalyst supported on ZrO2
catalyst with strong brønsted acid site
catalytic activity
catalytic activity of composites
catalytic properties
catalytic properties at a gas-solid interface
commercial liquid-phase batch process
continuous operation
Cs2.5H0.5PW12O40
decomposition of HPA
decreasing the catalyst acid strength
development of thermally stable HPA composites
DRIFTS
DRIFTS of adsorbed pyridine
enthalpy of ammonia adsorption
fixed-bed reactor
FTIR
gas phase
gas-phase isopropanol dehydration
gas-solid interface
GC analysis
H3PW12O40
H3PW12O40 heteropoly acid
H3PW12O40 supported at sub-monolayer coverage
H3PW12O40 supported at sub-monolayer coverage on Nb2O5
H3PW12O40 supported at sub-monolayer coverage on TiO2
H3PW12O40 supported at sub-monolayer coverage on ZrO2
heterogeneous acid catalysis
heterogeneous acid catalysis by heteropoly acids
heteropoly acid
HPA
HPA catalyst
HPA catalyst over zeolite
HPA catalyst supported on Nb2O5
HPA catalyst supported on TiO2
HPA catalyst supported on ZrO2
HPA catalyst with strong brønsted acid site
HPA composite
HPA supported on Nb2O5
HPA supported on TiO2
HPA supported on ZrO2
HPW
HPW (15 wt%) supported onto Nb2O5
HPW (15 wt%) supported onto SiO2
HPW (15 wt%) supported onto TiO2
HPW (15 wt%) supported onto ZrO2
HPW catalyst
HPW supported at sub-monolayer coverage on Nb2O5
HPW supported at sub-monolayer coverage on TiO2
HPW supported at sub-monolayer coverage on ZrO2
initial activity
initial enthalpy
initial enthalpy of ammonia adsorption
interaction between HPW and support
isomerisation
isomerisation of α-pinene
isopropanol
isopropanol dehydration
Keggin HPA
large organic molecule
Lewis acid
Lewis acid site
limonene
limonene yield

linear relationship
liquid-phase
liquid-phase batch process
MAS NMR
monoterpene
monoterpene by-products
Nb2O5
Nb2O5 as oxide matrix
NH3
NH3 adsorption calorimetry
on-line GC analysis
organic molecule
oxide matrix
partial decomposition
partial decomposition of HPA
p-cymene
pyridine
reactant molecule
reaction involving large organic molecule
silica-supported Cs2.5H0.5PW12O40
silica-supported H3PW12O40
SiO2
solid acid
solid acid catalysts
solid acid catalysts based on H3PW12O40
solid acid catalysts based on H3PW12O40 heteropoly acid
stable HPA composite
standart HPA catalyst
strong brønsted acid site
stronger acid site
strongest Keggin HPA
sub-monolayer
sub-monolayer coverage
sub-monolayer coverage on Nb2O5
support
terpene
terpinene
terpinolene
TGA
thermally stable HPA composite
TiO2
TIO2 as oxide matrix
TOF
tungsten
tungsten HPA
turnover frequency
wet impregnation
XRD
zeolite
ZrO2
ZrO2 as oxide matrix
α-pinene
α-pinene isomerisation
β-pinene

## The list of term-like n-grams

15%HPW/TIO2
15%HPW/TIO2 CATALYST
31P
31P MAS NMR
31P{1H}
31P{1H} MAS NMR
ACCESSIBILITY
ACCESSIBILITY OF REACTANT MOLECULE
ACID
ACID CATALYSIS
ACID SITE
ACID STRENGTH
ACID STRENGTH AS DETERMINED BY NH3 ADSORPTION
ACID STRENGTH OF HPA SUPPORTED ON NB2O5
ACIDIC COMPOSITE
ACIDIC ZEOLITE
ACIDITY
ACIDITY OF CATALYST
ACIDITY OF HPA CATALYST
ACTIVITY
ADSORBED PYRIDINE
ADSORPTION
ADSORPTION CALORIMETRY
AMMONIA
AMMONIA ADSORPTION CALORIMETRY
AQUEOUS SOLUTION
BATCH PROCESS
BET
BRØNSTED ACID
BRØNSTED ACID SITE
BY-PRODUCT
CAMPHENE
CAMPHENE YIELD
CATALYSIS
CATALYSIS BY HETEROPOLY ACID
CATALYST ACID
CATALYST ACID STRENGTH
CATALYST ACID STRENGTH AS DETERMINED BY NH3
CATALYST CHARACTERISATION
CATALYST DEACTIVATION
CATALYST OVER ZEOLITE
CATALYST SUPPORTED ON NB2O5
CATALYST SUPPORTED ON TIO2
CATALYST WITH STRONG BRØNSTED ACID SITE
CATALYTIC ACTIVITY
COMMERCIAL LIQUID-PHASE BATCH PROCESS
COMPOSITE
COMPOSITE AT A GAS-SOLID INTERFACE
CONTINUOUS OPERATION
COVERAGE
COVERAGE ON NB2O5
CS2.5H0.5PW12O40
DEACTIVATION
DECOMPOSITION
DECOMPOSITION OF HPA
DRIFTS OF ADSORBED PYRIDINE
ECONOMIC REWARD
ENTHALPY OF AMMONIA ADSORPTION
EP
EPSRC
FIXED-BED REACTOR
FIXED-BED REACTOR WITH ON-LINE GC ANALYSIS
FTIR

GAS PHASE
GAS PHASE IN A FIXED-BED REACTOR
GAS-PHASE
GAS-PHASE ISOPROPANOL DEHYDRATION
GAS-SOLID INTERFACE
GC ANALYSIS
H3PW12O40
HETEROGENEOUS ACID
HETEROGENEOUS ACID CATALYSIS
HETEROPOLY ACID
HPA
HPA CATALYST
HPA CATALYST OVER ZEOLITE
HPA CATALYST SUPPORTED ON NB2O5
HPA CATALYST SUPPORTED ON TIO2
HPA CATALYST WITH STRONG BRØNSTED ACID SITE
HPA COMPOSITE
HPA SUPPORTED ON NB2O5
HPW
HPW CATALYST
HPW FROM AQUEOUS SOLUTION
IMPREGNATION
INITIAL ACTIVITY
INITIAL ENTHALPY
INITIAL ENTHALPY OF AMMONIA ADSORPTION
INITIAL HIGH ACTIVITY
INTERACTION BETWEEN HPW
INTERACTION BETWEEN THE SUPPORT
ISOMERISATION
ISOMERISATION OF α-pinene
ISOPROPANOL
ISOPROPANOL DEHYDRATION
KEGGIN HPA
LARGE ORGANIC MOLECULE
LEWIS
LEWIS ACID
LEWIS ACID SITE
LIMONENE
LIMONENE YIELD
LINE WITH THE CATALYST ACID STRENGTH
LINEAR RELATIONSHIP
LINEAR RELATIONSHIP BETWEEN LOG
LIQUID-PHASE
LIQUID-PHASE BATCH
LIQUID-PHASE BATCH PROCESS
LOW CAMPHENE YIELD
MAS NMR
MIXTURE WITH MONOTERPENE BY-PRODUCT
MONOTERPENE
MONOTERPENE BY-PRODUCT
NB2O5
NB2O5 HAVE
NH3
NH3 ADSORPTION CALORIMETRY
NMR
ON-LINE GC ANALYSIS
ORDER OF SUPPORT
ORGANIC MOLECULE
ORIGINATING FROM THE SUPPORT
OXIDE
OXIDE MATRIX
OXIDE WITH HPW
OXIDE WITH HPW FROM AQUEOUS SOLUTION
PARTIAL DECOMPOSITION
PARTIAL DECOMPOSITION OF HPA
P-CYMENE
PHASE IN A FIXED-BED REACTOR
PYRIDINE
REACTANT MOLECULE
REACTION INVOLVING
REACTION INVOLVING LARGE ORGANIC MOLECULE
REACTOR
REACTOR WITH ON-LINE GC ANALYSIS
RELATIONSHIP BETWEEN LOG
SILICA-SUPPORTED H3PW12O40
SIO2
STABLE HPA COMPOSITE
STRENGTH AS DETERMINED BY NH3 ADSORPTION CALORIMETRY
STRENGTH OF HPA SUPPORTED ON NB2O5
STRONG BRØNSTED ACID
STRONG BRØNSTED ACID SITE
STRONGER ACID SITE
SUB-MONOLAYER COVERAGE
SUB-MONOLAYER COVERAGE ON NB2O5
SUFFERED FROM CATALYST DEACTIVATION
SUPPORT
SUPPORT FROM THE EPSRC
SUPPORTED AT SUB-MONOLAYER COVERAGE
SUPPORTED AT SUB-MONOLAYER COVERAGE ON NB2O5
SUPPORTED HPW CATALYST
SUPPORTED ONTO SIO2
TERPENE
TERPINENE
TERPINOLENE
TGA
THERMALLY STABLE HPA COMPOSITE
TIO2
TIO2 AS OXIDE MATRIX
TOF
TOTAL CAMPHENE
TUNGSTEN
TUNGSTEN HPA
TURNOVER FREQUENCY
WEAKER ACID
WET IMPREGNATION
ZRO2
α-PINENE
α-PINENE ISOMERISATION
β-PINENE

## Term-like n-grams recognized as the general chemistry terms

ACID
ACID CATALYSIS
ACID SITE
ACID STRENGTH
ACIDITY
ACTIVITY

ADSORPTION
ADSORPTION CALORIMETRY
BATCH PROCESS
BRØNSTED ACID
BRØNSTED ACID SITE
CATALYSIS
CATALYST ACID
CATALYST DEACTIVATION
CATALYTIC ACTIVITY
COMPOSITE
CONTINUOUS OPERATION
DEACTIVATION
DECOMPOSITION
HETEROGENEOUS ACID
IMPREGNATION
INITIAL ACTIVITY
INITIAL ENTHALPY
LEWIS ACID
LEWIS ACID SITE
LIQUID-PHASE BATCH
ORGANIC MOLECULE
OXIDE MATRIX
PARTIAL DECOMPOSITION
REACTANT MOLECULE
STRONG BRØNSTED ACID
SUPPORT
TURNOVER FREQUENCY
WET IMPREGNATION

## Term-like n-grams with tag «COMP»

15%HPW/TIO2
15%HPW/TIO2 CATALYST

## Term-like n-grams with OSCAR tags

31P
31P MAS NMR
31P{1H}
31P{1H} MAS NMR
ACCESSIBILITY OF REACTANT MOLECULE
ACID
ACID CATALYSIS
ACID SITE
ACID STRENGTH
ACID STRENGTH AS DETERMINED BY NH3 ADSORPTION
ACID STRENGTH OF HPA SUPPORTED ON NB2O5
ACIDIC ZEOLITE
ACIDITY OF CATALYST
ACIDITY OF HPA CATALYST
ACTIVITY
ADSORBED PYRIDINE
ADSORPTION
ADSORPTION CALORIMETRY
AMMONIA
AMMONIA ADSORPTION CALORIMETRY
AQUEOUS SOLUTION
BATCH PROCESS
BET
BRØNSTED ACID SITE
CAMPHENE
CAMPHENE YIELD
CATALYSIS
CATALYSIS BY HETEROPOLY ACID
CATALYST ACID STRENGTH
CATALYST ACID STRENGTH AS DETERMINED BY NH3
CATALYST CHARACTERISATION
CATALYST DEACTIVATION
CATALYST OVER ZEOLITE
CATALYST SUPPORTED ON NB2O5
CATALYST SUPPORTED ON TIO2
CATALYST WITH STRONG BRØNSTED ACID SITE
COMMERCIAL LIQUID-PHASE BATCH PROCESS
COVERAGE ON NB2O5
CS2.5H0.5PW12O40
DEACTIVATION
DECOMPOSITION OF HPA
DRIFTS OF ADSORBED PYRIDINE
ENTHALPY OF AMMONIA ADSORPTION
EP
EPSRC
GAS-PHASE ISOPROPANOL DEHYDRATION
GC ANALYSIS
H3PW12O40
HETEROGENEOUS ACID CATALYSIS
HETEROPOLY ACID
HPA
HPA CATALYST
HPA CATALYST OVER ZEOLITE
HPA CATALYST SUPPORTED ON NB2O5
HPA CATALYST SUPPORTED ON TIO2
HPA CATALYST WITH STRONG BRØNSTED ACID SITE
HPA COMPOSITE
HPA SUPPORTED ON NB2O5
HPW
HPW CATALYST
HPW FROM AQUEOUS SOLUTION
HPW/TIO2
HPW/TIO2 CATALYST
IMPREGNATION
INITIAL ENTHALPY OF AMMONIA ADSORPTION
INTERACTION BETWEEN HPW
ISOMERISATION OF α-pinene
ISOPROPANOL
ISOPROPANOL DEHYDRATION
KEGGIN HPA
LARGE ORGANIC MOLECULE
LEWIS
LEWIS ACID SITE
LIMONENE
LIMONENE YIELD
LINE WITH THE CATALYST ACID STRENGTH
LIQUID-PHASE BATCH PROCESS
LOW CAMPHENE YIELD
MAS NMR
MIXTURE WITH MONOTERPENE BY-PRODUCT
MONOTERPENE
MONOTERPENE BY-PRODUCT
NB2O5

NH3
NH3 ADSORPTION CALORIMETRY
NMR
ORGANIC MOLECULE
OXIDE
OXIDE MATRIX
OXIDE WITH HPW
OXIDE WITH HPW FROM AQUEOUS SOLUTION
PARTIAL DECOMPOSITION OF HPA
P-CYMENE
PYRIDINE
REACTANT MOLECULE
REACTION INVOLVING
REACTION INVOLVING LARGE ORGANIC MOLECULE
SILICA-SUPPORTED H3PW12O40
SIO2
STABLE HPA COMPOSITE
STRENGTH AS DETERMINED BY NH3 ADSORPTION CALORIMETRY
STRENGTH OF HPA SUPPORTED ON NB2O5
STRONG BRØNSTED ACID SITE
STRONGER ACID SITE
SUB-MONOLAYER COVERAGE ON NB2O5
SUFFERED FROM CATALYST DEACTIVATION
SUPPORT FROM THE EPSRC
SUPPORTED AT SUB-MONOLAYER COVERAGE ON NB2O5
SUPPORTED HPW CATALYST
SUPPORTED ONTO SIO2
TERPENE
TERPINENE
TERPINOLENE
TGA
THERMALLY STABLE HPA COMPOSITE
TIO2
TIO2 AS OXIDE MATRIX
TOF
TOTAL CAMPHENE
TUNGSTEN
TUNGSTEN HPA
WEAKER ACID
ZRO2
α-pinene
α-pinene ISOMERISATION
β-pinene

## The list of filtered off n-grams

0.15
0.51
0.58
"
" standard
" standard ”
" standard ” HPA
" standard ” HPA catalyst
" standard ” HPA catalyst such
" standard ” HPA catalyst such as
,
, ΔHi
, ΔHi -LRB-
, ΔHi -LRB- Figure
, ΔHi -LRB- Figure 1
, ΔHi -LRB- Figure 1 -RRB-
, 31P
, 31P MAS
, 31P MAS NMR
, 31P MAS NMR and
, 31P MAS NMR and FTIR
, 31P MAS NMR and FTIR indicate
, although
, although weaker
, although weaker acid
, although weaker acid ,
, although weaker acid , showed
, although weaker acid , showed more
, but
, but suffered
, but suffered from
, but suffered from catalyst
, but suffered from catalyst deactivation
, but suffered from catalyst deactivation ,
, causing
, causing partial
, causing partial decomposition
, causing partial decomposition of
, causing partial decomposition of HPA
, causing partial decomposition of HPA and
, decreasing
, decreasing the
, decreasing the catalyst
, decreasing the catalyst acid
, decreasing the catalyst acid strength
, decreasing the catalyst acid strength in
, especially
, especially for
, especially for reaction
, especially for reaction involving
, especially for reaction involving large
, especially for reaction involving large organic
, focusing
, focusing on
, focusing on acidic
, focusing on acidic composite
, focusing on acidic composite comprising
, focusing on acidic composite comprising tungsten
, FTIR
, FTIR and
, FTIR and 31P{1H}
, FTIR and 31P{1H} MAS
, FTIR and 31P{1H} MAS NMR
, FTIR and 31P{1H} MAS NMR -LRB-
, H3PW12O40
, H3PW12O40 -LRB-
, H3PW12O40 -LRB- HPW
, H3PW12O40 -LRB- HPW -RRB-
, H3PW12O40 -LRB- HPW -RRB- supported
, H3PW12O40 -LRB- HPW -RRB- supported at
, interaction
, interaction between
, interaction between HPW
, interaction between HPW and
, interaction between HPW and support

, interaction between HPW and support increase
, Nb2O5
, Nb2O5 ,
, Nb2O5 , ZrO2
, Nb2O5 , ZrO2 and
, Nb2O5 , ZrO2 and TiO2
, Nb2O5 , ZrO2 and TiO2 were
, p-cymene
, p-cymene and
, p-cymene and other
, possessing
, possessing strong
, possessing strong BrØnsted
, possessing strong BrØnsted acid
, possessing strong BrØnsted acid site
, possessing strong BrØnsted acid site ,
, resulting
, resulting in
, resulting in low
, resulting in low camphene
, resulting in low camphene yield
, showed
, showed more
, showed more stable
, showed more stable performance
, showed more stable performance in
, showed more stable performance in α-pinene
, terpinene
, terpinene ,
, terpinene , β-pinene
, terpinene , β-pinene ,
, terpinene , β-pinene , p-cymene
, terpinene , β-pinene , p-cymene and
, terpinolene
, terpinolene ,
, terpinolene , terpinene
, terpinolene , terpinene ,
, terpinolene , terpinene , β-pinene
, terpinolene , terpinene , β-pinene ,
, TGA
, TGA ,
, TGA , XRD
, TGA , XRD ,
, TGA , XRD , FTIR
, TGA , XRD , FTIR and
, the
, the catalyst
, the catalyst supported
, the catalyst supported on
, the catalyst supported on TiO2
, the catalyst supported on TiO2 ,
, the HPA
, the HPA catalyst
, the HPA catalyst supported
, the HPA catalyst supported on
, the HPA catalyst supported on Nb2O5
, there
, there has
, there has been
, there has been considerable
, there has been considerable research
, there has been considerable research activity
, TOF
, TOF -RRB-
, TOF -RRB- in
, TOF -RRB- in the
, TOF -RRB- in the gas-phase
, TOF -RRB- in the gas-phase isopropanol
, which
, which is
, which is similar
, which is similar to
, which is similar to the
, which is similar to the commercial
, with
, with the
, with the advantage
, with the advantage of
, with the advantage of continuous
, with the advantage of continuous operation
, with the latter
, with the latter mainly
, with the latter mainly originating
, with the latter mainly originating from
, XRD
, XRD ,
, XRD , FTIR
, XRD , FTIR and
, XRD , FTIR and 31P{1H}
, XRD , FTIR and 31P{1H} MAS
, ZrO2
, ZrO2 and
, ZrO2 and Nb2O5
, ZrO2 and Nb2O5 were
, ZrO2 and Nb2O5 were compared
, ZrO2 and Nb2O5 were compared to
, ZrO2 and Nb2O5have
, ZrO2 and Nb2O5have both
, ZrO2 and Nb2O5have both BrØnsted
, ZrO2 and Nb2O5have both BrØnsted and
, ZrO2 and TiO2
, ZrO2 and TiO2 ,
, ZrO2 and TiO2 , although
, ZrO2 and TiO2 , although weaker
, ZrO2 and TiO2 as
, ZrO2 and TiO2 as oxide
, ZrO2 and TiO2 as oxide matrix
, ZrO2 and TiO2 is
, ZrO2 and TiO2 is similar
, ZrO2 and TiO2 is similar to
, ZrO2 and TiO2 were
, ZrO2 and TiO2 were prepared
, ZrO2 and TiO2 were prepared by
, β-pinene
, β-pinene ,
, β-pinene , p-cymene
, β-pinene , p-cymene and
, β-pinene , p-cymene and other
/
/ 1
/ 1 -RRB-
/ 1 -RRB- is
/ 1 -RRB- is acknowledged
/ Discussion

/ Discussion Catalyst
/ Discussion Catalyst characterisation
/ E039847
/ E039847 and
/ E039847 and EP
/ E039847 and EP /
/ E039847 and EP / F014686
/ E039847 and EP / F014686 /
/ F014686
/ F014686 /
/ F014686 / 1
/ F014686 / 1 -RRB-
/ F014686 / 1 -RRB- is
/ F014686 / 1 -RRB- is acknowledged
:
: SiO2
: SiO2 <
: SiO2 < TiO2
: SiO2 < TiO2 <
: SiO2 < TiO2 < Nb2O5
: SiO2 < TiO2 < Nb2O5 <
”
” HPA
” HPA catalyst
” HPA catalyst such
” HPA catalyst such as
” HPA catalyst such as bulk
” HPA catalyst such as bulk and
< Nb2O5
< Nb2O5 <
< Nb2O5 < ZrO2
< Nb2O5 < ZrO2 ,
< Nb2O5 < ZrO2 , causing
< Nb2O5 < ZrO2 , causing partial
< Nb2O5 < ZrO2 , decreasing
< Nb2O5 < ZrO2 , decreasing the
< TiO2
< TiO2 <
< TiO2 < Nb2O5
< TiO2 < Nb2O5 <
< TiO2 < Nb2O5 < ZrO2
< TiO2 < Nb2O5 < ZrO2 ,
< ZrO2
< ZrO2 ,
< ZrO2 , causing
< ZrO2 , causing partial
< ZrO2 , causing partial decomposition
< ZrO2 , causing partial decomposition of
< ZrO2 , decreasing
< ZrO2 , decreasing the
< ZrO2 , decreasing the catalyst
< ZrO2 , decreasing the catalyst acid
<
ΔHi
ΔHi for
ΔHi for bulk
ΔHi for bulk and
ΔHi for bulk and supported
ΔHi for bulk and supported HPW
ΔHi for bulk and supported HPW catalyst
ΔHi -LRB-
ΔHi -LRB- Figure
ΔHi -LRB- Figure 1
ΔHi -LRB- Figure 1 -RRB-
1
1 -RRB-
1 -RRB- is
1 -RRB- is acknowledged
15
15 wt%
15 wt% -RRB-
15 wt% -RRB- supported
15 wt% -RRB- supported onto
15 wt% -RRB- supported onto SiO2
15 wt% -RRB- supported onto SiO2 ,
15% HPW/TiO2
15% HPW/TiO2 catalyst
15% HPW/TiO2 catalyst gave
15% HPW/TiO2 catalyst gave a
15% HPW/TiO2 catalyst gave a camphene
15% HPW/TiO2 catalyst gave a camphene yield
2
2 -RRB-
2 -RRB- and
2 -RRB- and α-pinene
2 -RRB- and α-pinene isomerisation
2 -RRB- and α-pinene isomerisation -LRB-
2 -RRB- and α-pinene isomerisation -LRB- 3
200oC
200oC and
200oC and ambient
200oC and ambient pressure
200oC and ambient pressure -LRB-
200oC and ambient pressure -LRB- 3
200oC and ambient pressure -LRB- 3 -RRB-
3
3 -RRB-
3 -RRB- in
3 -RRB- in the
3 -RRB- in the gas
3 -RRB- in the gas phase
3 -RRB- in the gas phase in
31P MAS
31P MAS NMR and
31P MAS NMR and FTIR
31P MAS NMR and FTIR indicate
31P MAS NMR and FTIR indicate increasing
31P{1H} MAS
31P{1H} MAS NMR -LRB-
31P{1H} MAS NMR -LRB- 2
31P{1H} MAS NMR -LRB- 2 -RRB-
51% and
51% and a
51% and a total
51% and a total camphene
51% and a total camphene and
51% and a total camphene and limonene
58% ,
58% , which
58% , which is
58% , which is similar
58% , which is similar to
58% , which is similar to the
a

a camphene
a camphene yield
a camphene yield of
a camphene yield of 51%
a camphene yield of 51% and
a camphene yield of 51% and a
a challenge
a challenge for
a challenge for further
a challenge for further research
a fixed-bed
a fixed-bed reactor
a fixed-bed reactor with
a fixed-bed reactor with on-line
a fixed-bed reactor with on-line GC
a fixed-bed reactor with on-line GC analysis
a gas-solid
a gas-solid interface
a good
a good linear
a good linear relationship
a good linear relationship between
a good linear relationship between log
a good linear relationship between log -LRB-
a mixture
a mixture with
a mixture with monoterpene
a mixture with monoterpene by-product
a mixture with monoterpene by-product such
a mixture with monoterpene by-product such as
a total
a total camphene
a total camphene and
a total camphene and limonene
a total camphene and limonene yield
a total camphene and limonene yield of
accessibility of
accessibility of reactant
accessibility of reactant molecule ,
accessibility of reactant molecule , especially
accessibility of reactant molecule , especially for
acid ,
acid , showed
acid , showed more
acid , showed more stable
acid , showed more stable performance
acid , showed more stable performance in
acid catalysis by
acid catalysis by heteropoly
acid catalysis by heteropoly acid
acid catalysis by heteropoly acid -LRB-
acid catalysis by heteropoly acid -LRB- hpa
acid -LRB-
acid -LRB- hpa
acid -LRB- hpa -RRB-
acid -LRB- hpa -RRB- has
acid -LRB- hpa -RRB- has attracted
acid -LRB- hpa -RRB- has attracted much
acid site ,
acid site , the
acid site , the catalyst
acid site , the catalyst supported
acid site , the catalyst supported on
acid site , with
acid site , with the
acid site , with the latter
acid site , with the latter mainly
acid site exhibited
acid site exhibited high
acid site exhibited high initial
acid site exhibited high initial activity
acid site exhibited high initial activity ,
acid site remains
acid site remains a
acid site remains a challenge
acid site remains a challenge for
acid site remains a challenge for further
acid strength as
acid strength as determined
acid strength as determined by
acid strength as determined by NH3
acid strength in
acid strength in that
acid strength in that order
acid strength in that order -LRB-
acid strength in that order -LRB- 2
acid strength of
acid strength of HPA
acid strength of HPA supported
acid strength of HPA supported on
acid strength of these
acid strength of these catalyst
acid strength of these catalyst is
acid strength of these catalyst is weaker
acidic
acidic composite comprising
acidic composite comprising tungsten
acidic composite comprising tungsten hpa
acidic composite comprising tungsten hpa and
acidic composite comprising tungsten hpa and Nb2O5
acidity and
acidity and catalytic
acidity and catalytic activity
acidity and catalytic activity of
acidity and catalytic activity of such
acidity and catalytic activity of such composite
acidity of
acidity of catalyst was
acidity of catalyst was assessed
acidity of catalyst was assessed by
acidity of catalyst was assessed by DRIFTS
acidity of HPA
acknowledged
activity ,
activity , but
activity , but suffered
activity , but suffered from
activity , but suffered from catalyst
activity , but suffered from catalyst deactivation
activity , focusing
activity , focusing on
activity , focusing on acidic
activity , focusing on acidic composite
activity , focusing on acidic composite comprising

activity -LRB-
activity -LRB- turnover
activity -LRB- turnover frequency
activity -LRB- turnover frequency ,
activity -LRB- turnover frequency , TOF
activity -LRB- turnover frequency , TOF -RRB-
activity of
activity of such
activity of such composite
activity of such composite at
activity of such composite at a
activity of such composite at a gas-solid
adsorbed
adsorbed pyridine and
adsorbed pyridine and NH3
adsorbed pyridine and NH3 adsorption
adsorbed pyridine and NH3 adsorption calorimetry
adsorbed pyridine and NH3 adsorption calorimetry -LRB-
adsorption calorimetry ,
adsorption calorimetry , 31P
adsorption calorimetry , 31P MAS
adsorption calorimetry , 31P MAS NMR
adsorption calorimetry , 31P MAS NMR and
adsorption calorimetry give
adsorption calorimetry give consistent
adsorption calorimetry give consistent result
adsorption calorimetry give consistent result regarding
adsorption calorimetry give consistent result regarding the
adsorption calorimetry -LRB-
adsorption calorimetry -LRB- 2
adsorption calorimetry -LRB- 2 -RRB-
adsorption for
adsorption for these
adsorption for these catalyst
adsorption for these catalyst ,
adsorption for these catalyst , ΔHi
adsorption for these catalyst , ΔHi -LRB-
advantage
advantage of
advantage of continuous
advantage of continuous operation
advantage of these
advantage of these HPA
advantage of these HPA catalyst
advantage of these HPA catalyst over
advantage of these HPA catalyst over zeolite
aim
aim of
aim of this
aim of this work
aim of this work is
aim of this work is to
aim of this work is to study
although
although weaker
although weaker acid
although weaker acid ,
although weaker acid , showed
although weaker acid , showed more
although weaker acid , showed more stable
ambient
ambient pressure
ambient pressure -LRB-
ambient pressure -LRB- 3
ambient pressure -LRB- 3 -RRB-
Ammonia adsorption
Ammonia adsorption calorimetry ,
Ammonia adsorption calorimetry , 31P
Ammonia adsorption calorimetry , 31P MAS
Ammonia adsorption calorimetry , 31P MAS NMR
ammonia adsorption for
ammonia adsorption for these
ammonia adsorption for these catalyst
ammonia adsorption for these catalyst ,
ammonia adsorption for these catalyst , ΔHi
analysis
and
and ΔHi
and ΔHi for
and ΔHi for bulk
and ΔHi for bulk and
and ΔHi for bulk and supported
and ΔHi for bulk and supported HPW
and 31P{1H}
and 31P{1H} MAS
and 31P{1H} MAS NMR
and 31P{1H} MAS NMR -LRB-
and 31P{1H} MAS NMR -LRB- 2
and 31P{1H} MAS NMR -LRB- 2 -RRB-
and a
and a total
and a total camphene
and a total camphene and
and a total camphene and limonene
and a total camphene and limonene yield
and ambient
and ambient pressure
and ambient pressure -LRB-
and ambient pressure -LRB- 3
and ambient pressure -LRB- 3 -RRB-
and catalytic
and catalytic activity
and catalytic activity of
and catalytic activity of such
and catalytic activity of such composite
and catalytic activity of such composite at
and characterised
and characterised by
and characterised by BET
and characterised by BET ,
and characterised by BET , TGA
and characterised by BET , TGA ,
and Cs2.5H0.5PW12O40
and decreasing
and decreasing the
and decreasing the catalyst
and decreasing the catalyst acid
and decreasing the catalyst acid strength
and decreasing the catalyst acid strength in
and EP
and EP /
and EP / F014686

and EP / F014686 /
and EP / F014686 / 1
and EP / F014686 / 1 -RRB-
and FTIR
and FTIR indicate
and FTIR indicate increasing
and FTIR indicate increasing interaction
and FTIR indicate increasing interaction between
and FTIR indicate increasing interaction between support
and green
and green benefit
and green benefit -LRB-
and green benefit -LRB- 1
and green benefit -LRB- 1 -RRB-
and HPA
and HPA in
and HPA in the
and HPA in the following
and HPA in the following order
and HPA in the following order of
and Lewis
and Lewis acid
and Lewis acid site
and Lewis acid site ,
and Lewis acid site , with
and Lewis acid site , with the
and limonene
and limonene yield
and limonene yield of
and limonene yield of 58%
and limonene yield of 58% ,
and limonene yield of 58% , which
and Nb2O5
and Nb2O5 ,
and Nb2O5 , ZrO2
and Nb2O5 , ZrO2 and
and Nb2O5 , ZrO2 and TiO2
and Nb2O5 , ZrO2 and TiO2 as
and Nb2O5 were
and Nb2O5 were compared
and Nb2O5 were compared to
and Nb2O5 were compared to "
and Nb2O5 were compared to " standard
and Nb2O5have
and Nb2O5have both
and Nb2O5have both BrØnsted
and Nb2O5have both BrØnsted and
and Nb2O5have both BrØnsted and Lewis
and Nb2O5have both BrØnsted and Lewis acid
and NH3
and NH3 adsorption
and NH3 adsorption calorimetry
and NH3 adsorption calorimetry give
and NH3 adsorption calorimetry give consistent
and NH3 adsorption calorimetry give consistent result
and NH3 adsorption calorimetry -LRB-
and NH3 adsorption calorimetry -LRB- 2
and NH3 adsorption calorimetry -LRB- 2 -RRB-
and other
and silica-supported
and silica-supported H3PW12O40
and silica-supported H3PW12O40 and
and silica-supported H3PW12O40 and
Cs2.5H0.5PW12O40
and support
and support increase
and support increase in
and support increase in the
and support increase in the series
and support increase in the series of
and supported
and supported HPW
and supported HPW catalyst
and the
and the initial
and the initial enthalpy
and the initial enthalpy of
and the initial enthalpy of ammonia
and the initial enthalpy of ammonia adsorption
and TiO2
and TiO2 ,
and TiO2 , although
and TiO2 , although weaker
and TiO2 , although weaker acid
and TiO2 , although weaker acid ,
and TiO2 as
and TiO2 as oxide
and TiO2 as oxide matrix
and TiO2 is
and TiO2 is similar
and TiO2 is similar to
and TiO2 is similar to that
and TiO2 is similar to that of
and TiO2 were
and TiO2 were prepared
and TiO2 were prepared by
and TiO2 were prepared by wet
and TiO2 were prepared by wet impregnation
and α-pinene
and α-pinene isomerisation
and α-pinene isomerisation -LRB-
and α-pinene isomerisation -LRB- 3
and α-pinene isomerisation -LRB- 3 -RRB-
and α-pinene isomerisation -LRB- 3 -RRB- in
aqueous
aqueous solution and
aqueous solution and characterised
aqueous solution and characterised by
aqueous solution and characterised by BET
aqueous solution and characterised by BET ,
as
as bulk
as bulk and
as bulk and silica-supported
as bulk and silica-supported H3PW12O40
as bulk and silica-supported H3PW12O40 and
as bulk and silica-supported H3PW12O40 and
Cs2.5H0.5PW12O40
as determined
as determined by
as determined by NH3
as determined by NH3 adsorption
as determined by NH3 adsorption calorimetry
as limonene

as limonene ,
as limonene , terpinolene
as limonene , terpinolene ,
as limonene , terpinolene , terpinene
as limonene , terpinolene , terpinene ,
as oxide
as oxide matrix
as terpene
as the
as the main
as the main product
as the main product in
as the main product in a
as the main product in a mixture
assessed
assessed by
assessed by DRIFTS
assessed by DRIFTS of
assessed by DRIFTS of adsorbed
assessed by DRIFTS of adsorbed pyridine
assessed by DRIFTS of adsorbed pyridine and
at
at 200oC
at 200oC and
at 200oC and ambient
at 200oC and ambient pressure
at 200oC and ambient pressure -LRB-
at 200oC and ambient pressure -LRB- 3
at a
at a gas-solid
at a gas-solid interface
at sub-monolayer
at sub-monolayer coverage
at sub-monolayer coverage on
at sub-monolayer coverage on Nb2O5
at sub-monolayer coverage on Nb2O5 ,
at sub-monolayer coverage on Nb2O5 , ZrO2
attracted
attracted much
attracted much interest
attracted much interest because
attracted much interest because of
attracted much interest because of its
attracted much interest because of its potential
batch
batch process ,
batch process , with
batch process , with the
batch process , with the advantage
batch process , with the advantage of
because
because of
because of its
because of its potential
because of its potential to
because of its potential to generate
because of its potential to generate economic
been
been considerable
been considerable research
been considerable research activity
been considerable research activity ,
been considerable research activity , focusing
been considerable research activity , focusing on
benefit
benefit -LRB-
benefit -LRB- 1
benefit -LRB- 1 -RRB-
BET ,
BET , TGA
BET , TGA ,
BET , TGA , XRD
BET , TGA , XRD ,
BET , TGA , XRD , FTIR
better
better accessibility
better accessibility of
better accessibility of reactant
better accessibility of reactant molecule
better accessibility of reactant molecule ,
better accessibility of reactant molecule , especially
between
between HPW
between HPW and
between HPW and support
between HPW and support increase
between HPW and support increase in
between HPW and support increase in the
between log
between log -LRB-
between log -LRB- TOF
between log -LRB- TOF -RRB-
between log -LRB- TOF -RRB- and
between log -LRB- TOF -RRB- and ΔHi
between log -LRB- TOF -RRB- and the
between support
between support and
between support and HPA
between support and HPA in
between support and HPA in the
between support and HPA in the following
both
both BrØnsted
both BrØnsted and
both BrØnsted and Lewis
both BrØnsted and Lewis acid
both BrØnsted and Lewis acid site
both BrØnsted and Lewis acid site ,
BrØnsted
BrØnsted acid
BrØnsted acid site ,
BrØnsted acid site , the
BrØnsted acid site , the catalyst
BrØnsted acid site , the catalyst supported
BrØnsted acid site exhibited
BrØnsted acid site exhibited high
BrØnsted acid site exhibited high initial
BrØnsted acid site exhibited high initial activity
BrØnsted and
BrØnsted and Lewis
BrØnsted and Lewis acid
BrØnsted and Lewis acid site
BrØnsted and Lewis acid site ,
BrØnsted and Lewis acid site , with

bulk
bulk and
bulk and silica-supported
bulk and silica-supported H3PW12O40
bulk and silica-supported H3PW12O40 and
bulk and silica-supported H3PW12O40 and Cs2.5H0.5PW12O40
bulk and supported
bulk and supported HPW
bulk and supported HPW catalyst
but
but suffered
but suffered from
but suffered from catalyst
but suffered from catalyst deactivation
but suffered from catalyst deactivation ,
but suffered from catalyst deactivation , resulting
by
by BET
by BET ,
by BET , TGA
by BET , TGA ,
by BET , TGA , XRD
by BET , TGA , XRD ,
by DRIFTS
by DRIFTS of
by DRIFTS of adsorbed
by DRIFTS of adsorbed pyridine
by DRIFTS of adsorbed pyridine and
by DRIFTS of adsorbed pyridine and NH3
by heteropoly
by heteropoly acid
by heteropoly acid -LRB-
by heteropoly acid -LRB- hpa
by heteropoly acid -LRB- hpa -RRB-
by heteropoly acid -LRB- hpa -RRB- has
by NH3
by NH3 adsorption
by NH3 adsorption calorimetry
by wet
by wet impregnation
by wet impregnation of
by wet impregnation of these
by wet impregnation of these oxide
by wet impregnation of these oxide with
by-product such
by-product such as
by-product such as limonene
by-product such as limonene ,
by-product such as limonene , terpinolene
by-product such as limonene , terpinolene ,
calorimetry
calorimetry ,
calorimetry , 31P
calorimetry , 31P MAS
calorimetry , 31P MAS NMR
calorimetry , 31P MAS NMR and
calorimetry , 31P MAS NMR and FTIR
calorimetry give
calorimetry give consistent
calorimetry give consistent result
calorimetry give consistent result regarding
calorimetry give consistent result regarding the
calorimetry give consistent result regarding the acidity
calorimetry -LRB-
calorimetry -LRB- 2
calorimetry -LRB- 2 -RRB-
camphene and
camphene and limonene
camphene and limonene yield
camphene and limonene yield of
camphene and limonene yield of 58%
camphene and limonene yield of 58% ,
camphene as
camphene as the
camphene as the main
camphene as the main product
camphene as the main product in
camphene as the main product in a
camphene yield of
camphene yield of 51%
camphene yield of 51% and
camphene yield of 51% and a
camphene yield of 51% and a total
catalysis by
catalysis by heteropoly
catalysis by heteropoly acid -LRB-
catalysis by heteropoly acid -LRB- hpa
catalysis by heteropoly acid -LRB- hpa -RRB-
catalyst
catalyst ,
catalyst , ΔHi
catalyst , ΔHi -LRB-
catalyst , ΔHi -LRB- Figure
catalyst , ΔHi -LRB- Figure 1
catalyst , ΔHi -LRB- Figure 1 -RRB-
catalyst acid
catalyst acid strength as
catalyst acid strength as determined
catalyst acid strength as determined by
catalyst acid strength in
catalyst acid strength in that
catalyst acid strength in that order
catalyst acid strength in that order -LRB-
catalyst comprising
catalyst comprising HPW
catalyst comprising HPW -LRB-
catalyst comprising HPW -LRB- 15
catalyst comprising HPW -LRB- 15 wt%
catalyst comprising HPW -LRB- 15 wt% -RRB-
catalyst deactivation ,
catalyst deactivation , resulting
catalyst deactivation , resulting in
catalyst deactivation , resulting in low
catalyst deactivation , resulting in low camphene
catalyst gave
catalyst gave a
catalyst gave a camphene
catalyst gave a camphene yield
catalyst gave a camphene yield of
catalyst gave a camphene yield of 51%
catalyst is
catalyst is weaker
catalyst is weaker than

catalyst is weaker than that
catalyst is weaker than that of
catalyst is weaker than that of HPW
catalyst over
catalyst over zeolite is
catalyst over zeolite is in
catalyst over zeolite is in better
catalyst over zeolite is in better accessibility
catalyst such
catalyst such as
catalyst such as bulk
catalyst such as bulk and
catalyst such as bulk and silica-supported
catalyst such as bulk and silica-supported $H_3PW_{12}O_{40}$
catalyst supported
catalyst supported on
catalyst supported on $Nb_2O_5$ ,
catalyst supported on $Nb_2O_5$ , $ZrO_2$
catalyst supported on $Nb_2O_5$ , $ZrO_2$ and
catalyst supported on $TiO_2$ ,
catalyst supported on $TiO_2$ , $ZrO_2$
catalyst supported on $TiO_2$ , $ZrO_2$ and
catalyst under
catalyst under study
catalyst under study comprise
catalyst under study comprise the
catalyst under study comprise the strongest
catalyst under study comprise the strongest Keggin
catalyst was
catalyst was assessed
catalyst was assessed by
catalyst was assessed by DRIFTS
catalyst was assessed by DRIFTS of
catalyst was assessed by DRIFTS of adsorbed
catalyst were
catalyst were tested
catalyst were tested in
catalyst were tested in isopropanol
catalyst were tested in isopropanol dehydration
catalyst were tested in isopropanol dehydration -LRB-
catalyst with
catalyst with strong
catalyst with strong BrØnsted
catalyst with strong BrØnsted acid
catalyst with strong BrØnsted acid site exhibited
catalytic
catalytic activity -LRB-
catalytic activity -LRB- turnover
catalytic activity -LRB- turnover frequency
catalytic activity -LRB- turnover frequency ,
catalytic activity -LRB- turnover frequency , TOF
catalytic activity of
catalytic activity of such
catalytic activity of such composite
catalytic activity of such composite at
catalytic activity of such composite at a
causing
causing partial
causing partial decomposition
causing partial decomposition of
causing partial decomposition of HPA
causing partial decomposition of HPA and
causing partial decomposition of HPA and decreasing
challenge
challenge for
challenge for further
challenge for further research
characterisation
characterised
characterised by
characterised by BET
characterised by BET ,
characterised by BET , TGA
characterised by BET , TGA ,
characterised by BET , TGA , XRD
commercial
commercial liquid-phase
commercial liquid-phase batch
commercial liquid-phase batch process ,
commercial liquid-phase batch process , with
commercial liquid-phase batch process , with the
compared
compared to
compared to "
compared to " standard
compared to " standard ”
compared to " standard ” HPA
compared to " standard ” HPA catalyst
composite at
composite at a
composite at a gas-solid
composite comprising
composite comprising tungsten
composite comprising tungsten hpa
composite comprising tungsten hpa and
composite comprising tungsten hpa and $Nb_2O_5$
composite comprising tungsten hpa and $Nb_2O_5$ ,
composite possessing
composite possessing stronger
composite possessing stronger acid
composite possessing stronger acid site
composite possessing stronger acid site remains
composite possessing stronger acid site remains a
comprise
comprise the
comprise the strongest
comprise the strongest Keggin
comprise the strongest Keggin HPA
comprise the strongest Keggin HPA ,
comprise the strongest Keggin HPA , $H_3PW_{12}O_{40}$
comprising
comprising HPW
comprising HPW -LRB-
comprising HPW -LRB- 15
comprising HPW -LRB- 15 wt%
comprising HPW -LRB- 15 wt% -RRB-
comprising HPW -LRB- 15 wt% -RRB- supported
comprising tungsten
comprising tungsten hpa
comprising tungsten hpa and
comprising tungsten hpa and $Nb_2O_5$
comprising tungsten hpa and $Nb_2O_5$ ,
comprising tungsten hpa and $Nb_2O_5$ , $ZrO_2$
conclusion

conclusion ,
conclusion , interaction
conclusion , interaction between
conclusion , interaction between HPW
conclusion , interaction between HPW and
conclusion , interaction between HPW and support
considerable
considerable research
considerable research activity
considerable research activity ,
considerable research activity , focusing
considerable research activity , focusing on
considerable research activity , focusing on acidic
consistent
consistent result
consistent result regarding
consistent result regarding the
consistent result regarding the acidity
consistent result regarding the acidity of
consistent result regarding the acidity of HPA
continuous
contrast
contrast to
contrast to the
contrast to the parent
contrast to the parent H3PW12O40
contrast to the parent H3PW12O40 ,
contrast to the parent H3PW12O40 , possessing
Conversely
Conversely ,
Conversely , the
Conversely , the HPA
Conversely , the HPA catalyst
Conversely , the HPA catalyst supported
Conversely , the HPA catalyst supported on
coverage on
coverage on Nb2O5 ,
coverage on Nb2O5 , ZrO2
coverage on Nb2O5 , ZrO2 and
coverage on Nb2O5 , ZrO2 and TiO2
deactivation ,
deactivation , resulting
deactivation , resulting in
deactivation , resulting in low
deactivation , resulting in low camphene
deactivation , resulting in low camphene yield
decomposition of
decomposition of HPA and
decomposition of HPA and decreasing
decomposition of HPA and decreasing the
decomposition of HPA and decreasing the catalyst
decrease
decrease in
decrease in line
decrease in line with
decrease in line with the
decrease in line with the catalyst
decrease in line with the catalyst acid
decreasing
decreasing the
decreasing the catalyst
decreasing the catalyst acid
decreasing the catalyst acid strength
decreasing the catalyst acid strength in
decreasing the catalyst acid strength in that
dehydration
dehydration and
dehydration and NH3
dehydration and NH3 adsorption
dehydration and NH3 adsorption calorimetry
dehydration and NH3 adsorption calorimetry give
dehydration and NH3 adsorption calorimetry give consistent
dehydration -LRB-
dehydration -LRB- 2
dehydration -LRB- 2 -RRB-
dehydration -LRB- 2 -RRB- and
dehydration -LRB- 2 -RRB- and α-pinene
dehydration -LRB- 2 -RRB- and α-pinene isomerisation
dehydration was
dehydration was found
dehydration was found to
dehydration was found to decrease
dehydration was found to decrease in
dehydration was found to decrease in line
determined
determined by
determined by NH3
determined by NH3 adsorption
determined by NH3 adsorption calorimetry
Development
Development of
Development of thermally
Development of thermally stable
Development of thermally stable HPA
Development of thermally stable HPA composite
Development of thermally stable HPA composite possessing
Discussion
Discussion Catalyst
Discussion Catalyst characterisation
DRIFTS
DRIFTS of
DRIFTS of adsorbed
DRIFTS of adsorbed pyridine and
DRIFTS of adsorbed pyridine and NH3
DRIFTS of adsorbed pyridine and NH3 adsorption
E039847
E039847 and
E039847 and EP
E039847 and EP /
E039847 and EP / F014686
E039847 and EP / F014686 /
E039847 and EP / F014686 / 1
economic
economic reward and
economic reward and green
economic reward and green benefit
economic reward and green benefit -LRB-
economic reward and green benefit -LRB- 1
enthalpy
enthalpy of
enthalpy of ammonia
enthalpy of ammonia adsorption for

enthalpy of ammonia adsorption for these
enthalpy of ammonia adsorption for these catalyst
EP /
EP / E039847
EP / E039847 and
EP / E039847 and EP
EP / E039847 and EP /
EP / E039847 and EP / F014686
EP / F014686
EP / F014686 /
EP / F014686 / 1
EP / F014686 / 1 -RRB-
EP / F014686 / 1 -RRB- is
EPSRC -LRB-
EPSRC -LRB- research
EPSRC -LRB- research grant
EPSRC -LRB- research grant EP
EPSRC -LRB- research grant EP /
EPSRC -LRB- research grant EP / E039847
especially
especially for
especially for reaction
especially for reaction involving
especially for reaction involving large
especially for reaction involving large organic
especially for reaction involving large organic molecule
exhibited
exhibited high
exhibited high initial
exhibited high initial activity
exhibited high initial activity ,
exhibited high initial activity , but
exhibited high initial activity , but suffered
Experimental
Experimental The
Experimental The catalyst
Experimental The catalyst comprising
Experimental The catalyst comprising HPW
Experimental The catalyst comprising HPW -LRB-
Experimental The catalyst comprising HPW -LRB- 15
F014686
F014686 /
F014686 / 1
F014686 / 1 -RRB-
F014686 / 1 -RRB- is
F014686 / 1 -RRB- is acknowledged
Figure
Figure 1
Figure 1 -RRB-
fixed-bed
fixed-bed reactor with
fixed-bed reactor with on-line
fixed-bed reactor with on-line GC
focusing
focusing on
focusing on acidic
focusing on acidic composite
focusing on acidic composite comprising
focusing on acidic composite comprising tungsten
focusing on acidic composite comprising tungsten hpa
following
following order
following order of
following order of support
following order of support :
following order of support : SiO2
following order of support : SiO2 <
for
for bulk
for bulk and
for bulk and supported
for bulk and supported HPW
for bulk and supported HPW catalyst
for further
for further research
for reaction
for reaction involving
for reaction involving large
for reaction involving large organic
for reaction involving large organic molecule
for reaction involving large organic molecule such
for these
for these catalyst
for these catalyst ,
for these catalyst , ΔHi
for these catalyst , ΔHi -LRB-
for these catalyst , ΔHi -LRB- Figure
found
found to
found to decrease
found to decrease in
found to decrease in line
found to decrease in line with
found to decrease in line with the
frequency
frequency ,
frequency , TOF
frequency , TOF -RRB-
frequency , TOF -RRB- in
frequency , TOF -RRB- in the
frequency , TOF -RRB- in the gas-phase
from
from aqueous
from aqueous solution
from aqueous solution and
from aqueous solution and characterised
from aqueous solution and characterised by
from aqueous solution and characterised by BET
from catalyst
from catalyst deactivation
from catalyst deactivation ,
from catalyst deactivation , resulting
from catalyst deactivation , resulting in
from catalyst deactivation , resulting in low
from the
from the EPSRC
from the EPSRC -LRB-
from the EPSRC -LRB- research
from the EPSRC -LRB- research grant
from the EPSRC -LRB- research grant EP
from the support
FTIR and
FTIR and 31P{1H}
FTIR and 31P{1H} MAS

FTIR and 31P{1H} MAS NMR
FTIR and 31P{1H} MAS NMR -LRB-
FTIR and 31P{1H} MAS NMR -LRB- 2
FTIR indicate
FTIR indicate increasing
FTIR indicate increasing interaction
FTIR indicate increasing interaction between
FTIR indicate increasing interaction between support
FTIR indicate increasing interaction between support and
further
further research
gas
gas phase at
gas phase at 200oC
gas phase at 200oC and
gas phase at 200oC and ambient
gas phase at 200oC and ambient pressure
gas phase in
gas phase in a
gas phase in a fixed-bed
gas phase in a fixed-bed reactor with
gas-phase isopropanol
gas-phase isopropanol dehydration was
gas-phase isopropanol dehydration was found
gas-phase isopropanol dehydration was found to
gas-phase isopropanol dehydration was found to decrease
gas-solid
gave
gave a
gave a camphene
gave a camphene yield
gave a camphene yield of
gave a camphene yield of 51%
gave a camphene yield of 51% and
GC
generate
generate economic
generate economic reward
generate economic reward and
generate economic reward and green
generate economic reward and green benefit
generate economic reward and green benefit -LRB-
give
give consistent
give consistent result
give consistent result regarding
give consistent result regarding the
give consistent result regarding the acidity
give consistent result regarding the acidity of
good
good linear
good linear relationship
good linear relationship between
good linear relationship between log
good linear relationship between log -LRB-
good linear relationship between log -LRB- TOF
grant
grant EP
grant EP /
grant EP / E039847
grant EP / E039847 and
grant EP / E039847 and EP
grant EP / E039847 and EP /
green
green benefit
green benefit -LRB-
green benefit -LRB- 1
green benefit -LRB- 1 -RRB-
H3PW12O40 ,
H3PW12O40 , possessing
H3PW12O40 , possessing strong
H3PW12O40 , possessing strong BrØnsted
H3PW12O40 , possessing strong BrØnsted acid
H3PW12O40 , possessing strong BrØnsted acid site
H3PW12O40 and
H3PW12O40 and Cs2.5H0.5PW12O40
H3PW12O40 -LRB-
H3PW12O40 -LRB- HPW
H3PW12O40 -LRB- HPW -RRB-
H3PW12O40 -LRB- HPW -RRB- supported
H3PW12O40 -LRB- HPW -RRB- supported at
H3PW12O40 -LRB- HPW -RRB- supported at sub-monolayer
has
has attracted
has attracted much
has attracted much interest
has attracted much interest because
has attracted much interest because of
has attracted much interest because of its
has been
has been considerable
has been considerable research
has been considerable research activity
has been considerable research activity ,
has been considerable research activity , focusing
Heterogeneous
Heterogeneous acid
Heterogeneous acid catalysis by
Heterogeneous acid catalysis by heteropoly
Heterogeneous acid catalysis by heteropoly acid
Heterogeneous acid catalysis by heteropoly acid -LRB-
heteropoly
heteropoly acid -LRB-
heteropoly acid -LRB- hpa
heteropoly acid -LRB- hpa -RRB-
heteropoly acid -LRB- hpa -RRB- has
heteropoly acid -LRB- hpa -RRB- has attracted
high
high initial
high initial activity
high initial activity ,
high initial activity , but
high initial activity , but suffered
high initial activity , but suffered from
HPA ,
HPA , H3PW12O40
HPA , H3PW12O40 -LRB-
HPA , H3PW12O40 -LRB- HPW
HPA , H3PW12O40 -LRB- HPW -RRB-
HPA , H3PW12O40 -LRB- HPW -RRB- supported
hpa and
HPA and decreasing
HPA and decreasing the

HPA and decreasing the catalyst
HPA and decreasing the catalyst acid
HPA and decreasing the catalyst acid strength
hpa and Nb2O5
hpa and Nb2O5 ,
hpa and Nb2O5 , ZrO2
hpa and Nb2O5 , ZrO2 and
hpa and Nb2O5 , ZrO2 and TiO2
HPA catalyst over
HPA catalyst over zeolite is
HPA catalyst over zeolite is in
HPA catalyst over zeolite is in better
HPA catalyst such
HPA catalyst such as
HPA catalyst such as bulk
HPA catalyst such as bulk and
HPA catalyst such as bulk and silica-supported
HPA catalyst supported
HPA catalyst supported on
HPA catalyst supported on Nb2O5 ,
HPA catalyst supported on Nb2O5 , ZrO2
HPA catalyst supported on TiO2 ,
HPA catalyst supported on TiO2 , ZrO2
HPA catalyst with
HPA catalyst with strong
HPA catalyst with strong BrØnsted
HPA catalyst with strong BrØnsted acid
HPA composite possessing stronger
HPA composite possessing stronger acid
HPA composite possessing stronger acid site
HPA composite possessing stronger acid site remains
HPA in
HPA in the
HPA in the following
HPA in the following order
HPA in the following order of
HPA in the following order of support
hpa -RRB-
hpa -RRB- has
hpa -RRB- has attracted
hpa -RRB- has attracted much
hpa -RRB- has attracted much interest
hpa -RRB- has attracted much interest because
HPA supported
HPA supported on
HPA supported on Nb2O5 ,
HPA supported on Nb2O5 , ZrO2
HPA supported on Nb2O5 , ZrO2 and
HPW and
HPW and Cs2.5H0.5PW12O40
HPW and support
HPW and support increase
HPW and support increase in
HPW and support increase in the
HPW and support increase in the series
HPW from
HPW from aqueous
HPW from aqueous solution and
HPW from aqueous solution and characterised
HPW from aqueous solution and characterised by
HPW -LRB-
HPW -LRB- 15
HPW -LRB- 15 wt%
HPW -LRB- 15 wt% -RRB-
HPW -LRB- 15 wt% -RRB- supported
HPW -LRB- 15 wt% -RRB- supported onto
HPW -RRB-
HPW -RRB- supported
HPW -RRB- supported at
HPW -RRB- supported at sub-monolayer
HPW -RRB- supported at sub-monolayer coverage
HPW -RRB- supported at sub-monolayer coverage on
HPW/TiO2 catalyst gave
HPW/TiO2 catalyst gave a
HPW/TiO2 catalyst gave a camphene
HPW/TiO2 catalyst gave a camphene yield
HPW/TiO2 catalyst gave a camphene yield of
impregnation of
impregnation of these
impregnation of these oxide
impregnation of these oxide with
impregnation of these oxide with HPW
impregnation of these oxide with HPW from
In
in a
in a fixed-bed
in a fixed-bed reactor
in a fixed-bed reactor with
in a fixed-bed reactor with on-line
in a fixed-bed reactor with on-line GC
in a mixture
in a mixture with
in a mixture with monoterpene
in a mixture with monoterpene by-product
in a mixture with monoterpene by-product such
in better
in better accessibility
in better accessibility of
in better accessibility of reactant
in better accessibility of reactant molecule
in better accessibility of reactant molecule ,
In conclusion
In conclusion ,
In conclusion , interaction
In conclusion , interaction between
In conclusion , interaction between HPW
In conclusion , interaction between HPW and
In contrast
In contrast to
In contrast to the
In contrast to the parent
In contrast to the parent H3PW12O40
In contrast to the parent H3PW12O40 ,
in isopropanol
in isopropanol dehydration
in isopropanol dehydration -LRB-
in isopropanol dehydration -LRB- 2
in isopropanol dehydration -LRB- 2 -RRB-
in isopropanol dehydration -LRB- 2 -RRB- and
in line
in line with
in line with the
in line with the catalyst
in line with the catalyst acid

in line with the catalyst acid strength
in low
in low camphene
in low camphene yield
In recent
In recent year
In recent year ,
In recent year , there
In recent year , there has
In recent year , there has been
in that
in that order
in that order -LRB-
in that order -LRB- 2
in that order -LRB- 2 -RRB-
in the
in the following
in the following order
in the following order of
in the following order of support
in the following order of support :
in the gas
in the gas phase
in the gas phase at
in the gas phase at 200oC
in the gas phase at 200oC and
in the gas phase in
in the gas phase in a
in the gas phase in a fixed-bed
in the gas-phase
in the gas-phase isopropanol
in the gas-phase isopropanol dehydration
in the gas-phase isopropanol dehydration was
in the gas-phase isopropanol dehydration was found
in the series
in the series of
in the series of support
in the series of support :
in the series of support : SiO2
in α-pinene
in α-pinene isomerisation
increase
increase in
increase in the
increase in the series
increase in the series of
increase in the series of support
increase in the series of support :
increasing
increasing interaction
increasing interaction between
increasing interaction between support
increasing interaction between support and
increasing interaction between support and HPA
increasing interaction between support and HPA in
indicate
indicate increasing
indicate increasing interaction
indicate increasing interaction between
indicate increasing interaction between support
indicate increasing interaction between support and
indicate increasing interaction between support and HPA
initial
initial activity ,
initial activity , but
initial activity , but suffered
initial activity , but suffered from
initial activity , but suffered from catalyst
initial enthalpy of
initial enthalpy of ammonia
initial enthalpy of ammonia adsorption for
initial enthalpy of ammonia adsorption for these
interaction
interaction between
interaction between HPW and
interaction between HPW and support
interaction between HPW and support increase
interaction between HPW and support increase in
interaction between support
interaction between support and
interaction between support and HPA
interaction between support and HPA in
interaction between support and HPA in the
interest
interest because
interest because of
interest because of its
interest because of its potential
interest because of its potential to
interest because of its potential to generate
interface
Introduction
Introduction Heterogeneous
Introduction Heterogeneous acid
Introduction Heterogeneous acid catalysis
Introduction Heterogeneous acid catalysis by
Introduction Heterogeneous acid catalysis by heteropoly
Introduction Heterogeneous acid catalysis by heteropoly acid
involving
involving large
involving large organic
involving large organic molecule
involving large organic molecule such
involving large organic molecule such as
involving large organic molecule such as terpene
is
is a
is a good
is a good linear
is a good linear relationship
is a good linear relationship between
is a good linear relationship between log
is acknowledged
is in
is in better
is in better accessibility
is in better accessibility of
is in better accessibility of reactant
is in better accessibility of reactant molecule
is similar
is similar to
is similar to that
is similar to that of

is similar to that of acidic
is similar to that of acidic zeolite
is similar to the
is similar to the commercial
is similar to the commercial liquid-phase
is similar to the commercial liquid-phase batch
is to
is to study
is to study the
is to study the acidity
is to study the acidity and
is to study the acidity and catalytic
is weaker
is weaker than
is weaker than that
is weaker than that of
is weaker than that of HPW
is weaker than that of HPW and
isomerisation -LRB-
isomerisation -LRB- 3
isomerisation -LRB- 3 -RRB-
isomerisation -LRB- 3 -RRB- in
isomerisation -LRB- 3 -RRB- in the
isomerisation -LRB- 3 -RRB- in the gas
isomerisation of
isomerisation of α-pinene was
isomerisation of α-pinene was studied
isomerisation of α-pinene was studied in
isomerisation of α-pinene was studied in the
isopropanol dehydration and
isopropanol dehydration and NH3
isopropanol dehydration and NH3 adsorption
isopropanol dehydration and NH3 adsorption calorimetry
isopropanol dehydration and NH3 adsorption calorimetry give
isopropanol dehydration -LRB-
isopropanol dehydration -LRB- 2
isopropanol dehydration -LRB- 2 -RRB-
isopropanol dehydration -LRB- 2 -RRB- and
isopropanol dehydration -LRB- 2 -RRB- and α-pinene
isopropanol dehydration was
isopropanol dehydration was found
isopropanol dehydration was found to
isopropanol dehydration was found to decrease
isopropanol dehydration was found to decrease in
its
its potential
its potential to
its potential to generate
its potential to generate economic
its potential to generate economic reward
its potential to generate economic reward and
Keggin
Keggin HPA ,
Keggin HPA , H3PW12O40
Keggin HPA , H3PW12O40 -LRB-
Keggin HPA , H3PW12O40 -LRB- HPW
Keggin HPA , H3PW12O40 -LRB- HPW -RRB-
large
large organic
large organic molecule such
large organic molecule such as
large organic molecule such as terpene
latter
latter mainly
latter mainly originating
latter mainly originating from
latter mainly originating from the
latter mainly originating from the support
Lewis acid
Lewis acid site ,
Lewis acid site , with
Lewis acid site , with the
Lewis acid site , with the latter
limonene ,
limonene , terpinolene
limonene , terpinolene ,
limonene , terpinolene , terpinene
limonene , terpinolene , terpinene ,
limonene , terpinolene , terpinene , β-pinene
limonene yield of
limonene yield of 58%
limonene yield of 58% ,
limonene yield of 58% , which
limonene yield of 58% , which is
line
line with
line with the
line with the catalyst
line with the catalyst acid
line with the catalyst acid strength as
linear
linear relationship between
linear relationship between log -LRB-
linear relationship between log -LRB- TOF
linear relationship between log -LRB- TOF -RRB-
liquid-phase batch
liquid-phase batch process ,
liquid-phase batch process , with
liquid-phase batch process , with the
liquid-phase batch process , with the advantage
log
log -LRB-
log -LRB- TOF
log -LRB- TOF -RRB-
log -LRB- TOF -RRB- and
log -LRB- TOF -RRB- and ΔHi
log -LRB- TOF -RRB- and ΔHi for
log -LRB- TOF -RRB- and the
log -LRB- TOF -RRB- and the initial
low
low camphene
-LRB-
-LRB- 1
-LRB- 1 -RRB-
-LRB- 15
-LRB- 15 wt%
-LRB- 15 wt% -RRB-
-LRB- 15 wt% -RRB- supported
-LRB- 15 wt% -RRB- supported onto
-LRB- 15 wt% -RRB- supported onto SiO2
-LRB- 2
-LRB- 2 -RRB-
-LRB- 2 -RRB- and

-LRB- 2 -RRB- and α-pinene
-LRB- 2 -RRB- and α-pinene isomerisation
-LRB- 2 -RRB- and α-pinene isomerisation -LRB-
-LRB- 3
-LRB- 3 -RRB-
-LRB- 3 -RRB- in
-LRB- 3 -RRB- in the
-LRB- 3 -RRB- in the gas
-LRB- 3 -RRB- in the gas phase
-LRB- Figure
-LRB- Figure 1
-LRB- Figure 1 -RRB-
-LRB- hpa
-LRB- hpa -RRB-
-LRB- hpa -RRB- has
-LRB- hpa -RRB- has attracted
-LRB- hpa -RRB- has attracted much
-LRB- hpa -RRB- has attracted much interest
-LRB- HPW
-LRB- HPW -RRB-
-LRB- HPW -RRB- supported
-LRB- HPW -RRB- supported at
-LRB- HPW -RRB- supported at sub-monolayer
-LRB- HPW -RRB- supported at sub-monolayer coverage
-LRB- research
-LRB- research grant
-LRB- research grant EP
-LRB- research grant EP /
-LRB- research grant EP / E039847
-LRB- research grant EP / E039847 and
-LRB- TOF
-LRB- TOF -RRB-
-LRB- TOF -RRB- and
-LRB- TOF -RRB- and ΔHi
-LRB- TOF -RRB- and ΔHi for
-LRB- TOF -RRB- and ΔHi for bulk
-LRB- TOF -RRB- and the
-LRB- TOF -RRB- and the initial
-LRB- TOF -RRB- and the initial enthalpy
-LRB- turnover
-LRB- turnover frequency
-LRB- turnover frequency ,
-LRB- turnover frequency , TOF
-LRB- turnover frequency , TOF -RRB-
-LRB- turnover frequency , TOF -RRB- in
main
main product
main product in
main product in a
main product in a mixture
main product in a mixture with
main product in a mixture with monoterpene
mainly
mainly originating
mainly originating from
mainly originating from the
mainly originating from the support
MAS
MAS NMR and
MAS NMR and FTIR
MAS NMR and FTIR indicate
MAS NMR and FTIR indicate increasing
MAS NMR and FTIR indicate increasing interaction
MAS NMR -LRB-
MAS NMR -LRB- 2
MAS NMR -LRB- 2 -RRB-
matrix
mixture
mixture with
mixture with monoterpene
mixture with monoterpene by-product such
mixture with monoterpene by-product such as
mixture with monoterpene by-product such as limonene
molecule
molecule ,
molecule , especially
molecule , especially for
molecule , especially for reaction
molecule , especially for reaction involving
molecule , especially for reaction involving large
molecule such
molecule such as
molecule such as terpene
monoterpene by-product such
monoterpene by-product such as
monoterpene by-product such as limonene
monoterpene by-product such as limonene ,
monoterpene by-product such as limonene , terpinolene
more
more stable
more stable performance
more stable performance in
more stable performance in α-pinene
more stable performance in α-pinene isomerisation
much
much interest
much interest because
much interest because of
much interest because of its
much interest because of its potential
much interest because of its potential to
Nb2O5 ,
Nb2O5 , ZrO2
Nb2O5 , ZrO2 and
Nb2O5 , ZrO2 and TiO2
Nb2O5 , ZrO2 and TiO2 ,
Nb2O5 , ZrO2 and TiO2 , although
Nb2O5 , ZrO2 and TiO2 as
Nb2O5 , ZrO2 and TiO2 as oxide
Nb2O5 , ZrO2 and TiO2 is
Nb2O5 , ZrO2 and TiO2 is similar
Nb2O5 , ZrO2 and TiO2 were
Nb2O5 , ZrO2 and TiO2 were prepared
Nb2O5 <
Nb2O5 < ZrO2
Nb2O5 < ZrO2 ,
Nb2O5 < ZrO2 , causing
Nb2O5 < ZrO2 , causing partial
Nb2O5 < ZrO2 , causing partial decomposition
Nb2O5 < ZrO2 , decreasing
Nb2O5 < ZrO2 , decreasing the
Nb2O5 < ZrO2 , decreasing the catalyst
Nb2O5 were

Nb2O5 were compared
Nb2O5 were compared to
Nb2O5 were compared to "
Nb2O5 were compared to " standard
Nb2O5 were compared to " standard ”
Nb2O5have both
Nb2O5have both BrØnsted
Nb2O5have both BrØnsted and
Nb2O5have both BrØnsted and Lewis
Nb2O5have both BrØnsted and Lewis acid
Nb2O5have both BrØnsted and Lewis acid site
NH3 adsorption
NH3 adsorption calorimetry give
NH3 adsorption calorimetry give consistent
NH3 adsorption calorimetry give consistent result
NH3 adsorption calorimetry give consistent result regarding
NH3 adsorption calorimetry -LRB-
NH3 adsorption calorimetry -LRB- 2
NH3 adsorption calorimetry -LRB- 2 -RRB-
NMR and
NMR and FTIR
NMR and FTIR indicate
NMR and FTIR indicate increasing
NMR and FTIR indicate increasing interaction
NMR and FTIR indicate increasing interaction between
NMR -LRB-
NMR -LRB- 2
NMR -LRB- 2 -RRB-
of
of 51%
of 51% and
of 51% and a
of 51% and a total
of 51% and a total camphene
of 51% and a total camphene and
of 58%
of 58% ,
of 58% , which
of 58% , which is
of 58% , which is similar
of 58% , which is similar to
of acidic
of acidic zeolite
of adsorbed
of adsorbed pyridine
of adsorbed pyridine and
of adsorbed pyridine and NH3
of adsorbed pyridine and NH3 adsorption
of adsorbed pyridine and NH3 adsorption calorimetry
of ammonia
of ammonia adsorption
of ammonia adsorption for
of ammonia adsorption for these
of ammonia adsorption for these catalyst
of ammonia adsorption for these catalyst ,
of catalyst
of catalyst was
of catalyst was assessed
of catalyst was assessed by
of catalyst was assessed by DRIFTS
of catalyst was assessed by DRIFTS of
of continuous
of continuous operation
of HPA
of HPA and
of HPA and decreasing
of HPA and decreasing the
of HPA and decreasing the catalyst
of HPA and decreasing the catalyst acid
of HPA catalyst
of HPA supported
of HPA supported on
of HPA supported on Nb2O5
of HPA supported on Nb2O5 ,
of HPA supported on Nb2O5 , ZrO2
of HPW
of HPW and
of HPW and Cs2.5H0.5PW12O40
of its
of its potential
of its potential to
of its potential to generate
of its potential to generate economic
of its potential to generate economic reward
of reactant
of reactant molecule
of reactant molecule ,
of reactant molecule , especially
of reactant molecule , especially for
of reactant molecule , especially for reaction
of such
of such composite
of such composite at
of such composite at a
of such composite at a gas-solid
of such composite at a gas-solid interface
of support
of support :
of support : SiO2
of support : SiO2 <
of support : SiO2 < TiO2
of support : SiO2 < TiO2 <
of thermally
of thermally stable
of thermally stable HPA
of thermally stable HPA composite
of thermally stable HPA composite possessing
of thermally stable HPA composite possessing stronger
of these
of these catalyst
of these catalyst is
of these catalyst is weaker
of these catalyst is weaker than
of these catalyst is weaker than that
of these HPA
of these HPA catalyst
of these HPA catalyst over
of these HPA catalyst over zeolite
of these HPA catalyst over zeolite is
of these oxide
of these oxide with
of these oxide with HPW
of these oxide with HPW from

of these oxide with HPW from aqueous
of this
of this work
of this work is
of this work is to
of this work is to study
of this work is to study the
of α-pinene
of α-pinene was
of α-pinene was studied
of α-pinene was studied in
of α-pinene was studied in the
of α-pinene was studied in the gas
on
on acidic
on acidic composite
on acidic composite comprising
on acidic composite comprising tungsten
on acidic composite comprising tungsten hpa
on acidic composite comprising tungsten hpa and
on Nb2O5
on Nb2O5 ,
on Nb2O5 , ZrO2
on Nb2O5 , ZrO2 and
on Nb2O5 , ZrO2 and TiO2
on Nb2O5 , ZrO2 and TiO2 ,
on Nb2O5 , ZrO2 and TiO2 is
on TiO2
on TiO2 ,
on TiO2 , ZrO2
on TiO2 , ZrO2 and
on TiO2 , ZrO2 and Nb2O5
on TiO2 , ZrO2 and Nb2O5 were
on TiO2 , ZrO2 and Nb2O5have
on TiO2 , ZrO2 and Nb2O5have both
on-line
on-line GC
onto
onto SiO2
onto SiO2 ,
onto SiO2 , Nb2O5
onto SiO2 , Nb2O5 ,
onto SiO2 , Nb2O5 , ZrO2
onto SiO2 , Nb2O5 , ZrO2 and
operation
order
order -LRB-
order -LRB- 2
order -LRB- 2 -RRB-
order of
order of support :
order of support : SiO2
order of support : SiO2 <
order of support : SiO2 < TiO2
organic
organic molecule such
organic molecule such as
organic molecule such as terpene
originating
originating from
originating from the
other
over
over zeolite
over zeolite is
over zeolite is in
over zeolite is in better
over zeolite is in better accessibility
over zeolite is in better accessibility of
oxide with
oxide with HPW from
oxide with HPW from aqueous
oxide with HPW from aqueous solution and
parent
parent H3PW12O40
parent H3PW12O40 ,
parent H3PW12O40 , possessing
parent H3PW12O40 , possessing strong
parent H3PW12O40 , possessing strong BrØnsted
parent H3PW12O40 , possessing strong BrØnsted acid
partial
partial decomposition of
partial decomposition of HPA and
partial decomposition of HPA and decreasing
partial decomposition of HPA and decreasing the
p-cymene and
p-cymene and other
performance
performance in
performance in α-pinene
performance in α-pinene isomerisation
phase
phase at
phase at 200oC
phase at 200oC and
phase at 200oC and ambient
phase at 200oC and ambient pressure
phase at 200oC and ambient pressure -LRB-
phase in
phase in a
phase in a fixed-bed
phase in a fixed-bed reactor with
phase in a fixed-bed reactor with on-line
possessing
possessing strong
possessing strong BrØnsted
possessing strong BrØnsted acid
possessing strong BrØnsted acid site
possessing strong BrØnsted acid site ,
possessing strong BrØnsted acid site , the
possessing stronger
possessing stronger acid
possessing stronger acid site
possessing stronger acid site remains
possessing stronger acid site remains a
possessing stronger acid site remains a challenge
potential
potential to
potential to generate
potential to generate economic
potential to generate economic reward
potential to generate economic reward and
potential to generate economic reward and green
prepared

prepared by
prepared by wet
prepared by wet impregnation
prepared by wet impregnation of
prepared by wet impregnation of these
prepared by wet impregnation of these oxide
pressure
pressure -LRB-
pressure -LRB- 3
pressure -LRB- 3 -RRB-
process
process ,
process , with
process , with the
process , with the advantage
process , with the advantage of
process , with the advantage of continuous
product
product in
product in a
product in a mixture
product in a mixture with
product in a mixture with monoterpene
product in a mixture with monoterpene by-product
pyridine and
pyridine and NH3
pyridine and NH3 adsorption
pyridine and NH3 adsorption calorimetry
pyridine and NH3 adsorption calorimetry -LRB-
pyridine and NH3 adsorption calorimetry -LRB- 2
reactant
reactant molecule ,
reactant molecule , especially
reactant molecule , especially for
reactant molecule , especially for reaction
reactant molecule , especially for reaction involving
reaction
reaction involving large
reaction involving large organic
reaction involving large organic molecule such
reaction involving large organic molecule such as
reaction yielded
reaction yielded camphene
reaction yielded camphene as
reaction yielded camphene as the
reaction yielded camphene as the main
reaction yielded camphene as the main product
reactor with
reactor with on-line
reactor with on-line GC
recent
recent year
recent year ,
recent year , there
recent year , there has
recent year , there has been
recent year , there has been considerable
regarding
regarding the
regarding the acidity
regarding the acidity of
regarding the acidity of HPA
regarding the acidity of HPA catalyst
relationship
relationship between
relationship between log -LRB-
relationship between log -LRB- TOF
relationship between log -LRB- TOF -RRB-
relationship between log -LRB- TOF -RRB- and
remains
remains a
remains a challenge
remains a challenge for
remains a challenge for further
remains a challenge for further research
research
research activity
research activity ,
research activity , focusing
research activity , focusing on
research activity , focusing on acidic
research activity , focusing on acidic composite
research grant
research grant EP
research grant EP /
research grant EP / E039847
research grant EP / E039847 and
research grant EP / E039847 and EP
result
result /
result / Discussion
result / Discussion Catalyst
result / Discussion Catalyst characterisation
result regarding
result regarding the
result regarding the acidity
result regarding the acidity of
result regarding the acidity of HPA
result regarding the acidity of HPA catalyst
resulting
resulting in
resulting in low
resulting in low camphene
resulting in low camphene yield
reward
reward and
reward and green
reward and green benefit
reward and green benefit -LRB-
reward and green benefit -LRB- 1
reward and green benefit -LRB- 1 -RRB-
-RRB-
-RRB- and
-RRB- and ΔHi
-RRB- and ΔHi for
-RRB- and ΔHi for bulk
-RRB- and ΔHi for bulk and
-RRB- and ΔHi for bulk and supported
-RRB- and the
-RRB- and the initial
-RRB- and the initial enthalpy
-RRB- and the initial enthalpy of
-RRB- and the initial enthalpy of ammonia
-RRB- and α-pinene

-RRB- and α-pinene isomerisation
-RRB- and α-pinene isomerisation -LRB-
-RRB- and α-pinene isomerisation -LRB- 3
-RRB- and α-pinene isomerisation -LRB- 3 -RRB-
-RRB- has
-RRB- has attracted
-RRB- has attracted much
-RRB- has attracted much interest
-RRB- has attracted much interest because
-RRB- has attracted much interest because of
-RRB- in
-RRB- in the
-RRB- in the gas
-RRB- in the gas phase
-RRB- in the gas phase in
-RRB- in the gas phase in a
-RRB- in the gas-phase
-RRB- in the gas-phase isopropanol
-RRB- in the gas-phase isopropanol dehydration
-RRB- in the gas-phase isopropanol dehydration was
-RRB- is
-RRB- is acknowledged
-RRB- supported
-RRB- supported at
-RRB- supported at sub-monolayer
-RRB- supported at sub-monolayer coverage
-RRB- supported at sub-monolayer coverage on
-RRB- supported at sub-monolayer coverage on Nb2O5
-RRB- supported onto
-RRB- supported onto SiO2
-RRB- supported onto SiO2 ,
-RRB- supported onto SiO2 , Nb2O5
-RRB- supported onto SiO2 , Nb2O5 ,
series
series of
series of support
series of support :
series of support : SiO2
series of support : SiO2 <
series of support : SiO2 < TiO2
showed
showed more
showed more stable
showed more stable performance
showed more stable performance in
showed more stable performance in α-pinene
showed more stable performance in α-pinene isomerisation
shows
shows that
shows that isopropanol
shows that isopropanol dehydration
shows that isopropanol dehydration and
shows that isopropanol dehydration and NH3
shows that isopropanol dehydration and NH3 adsorption
silica-supported
silica-supported H3PW12O40 and
silica-supported H3PW12O40 and Cs2.5H0.5PW12O40
similar
similar to
similar to that
similar to that of
similar to that of acidic
similar to that of acidic zeolite
similar to the
similar to the commercial
similar to the commercial liquid-phase
similar to the commercial liquid-phase batch
similar to the commercial liquid-phase batch process
SiO2 ,
SiO2 , Nb2O5
SiO2 , Nb2O5 ,
SiO2 , Nb2O5 , ZrO2
SiO2 , Nb2O5 , ZrO2 and
SiO2 , Nb2O5 , ZrO2 and TiO2
SiO2 <
SiO2 < TiO2
SiO2 < TiO2 <
SiO2 < TiO2 < Nb2O5
SiO2 < TiO2 < Nb2O5 <
SiO2 < TiO2 < Nb2O5 < ZrO2
site
site ,
site , the
site , the catalyst
site , the catalyst supported
site , the catalyst supported on
site , the catalyst supported on TiO2
site , with
site , with the
site , with the latter
site , with the latter mainly
site , with the latter mainly originating
site exhibited
site exhibited high
site exhibited high initial
site exhibited high initial activity
site exhibited high initial activity ,
site exhibited high initial activity , but
site remains
site remains a
site remains a challenge
site remains a challenge for
site remains a challenge for further
site remains a challenge for further research
solution
solution and
solution and characterised
solution and characterised by
solution and characterised by BET
solution and characterised by BET ,
solution and characterised by BET , TGA
stable
stable HPA
stable HPA composite possessing
stable HPA composite possessing stronger
stable HPA composite possessing stronger acid
stable HPA composite possessing stronger acid site
stable performance
stable performance in
stable performance in α-pinene
stable performance in α-pinene isomerisation
standard
standard ”

standard ” HPA
standard ” HPA catalyst
standard ” HPA catalyst such
standard ” HPA catalyst such as
standard ” HPA catalyst such as bulk
strength
strength as
strength as determined
strength as determined by
strength as determined by NH3
strength as determined by NH3 adsorption
strength in
strength in that
strength in that order
strength in that order -LRB-
strength in that order -LRB- 2
strength in that order -LRB- 2 -RRB-
strength of
strength of HPA
strength of HPA supported
strength of HPA supported on
strength of HPA supported on Nb2O5 ,
strength of these
strength of these catalyst
strength of these catalyst is
strength of these catalyst is weaker
strength of these catalyst is weaker than
strong
strong BrØnsted
strong BrØnsted acid
strong BrØnsted acid site ,
strong BrØnsted acid site , the
strong BrØnsted acid site , the catalyst
strong BrØnsted acid site exhibited
strong BrØnsted acid site exhibited high
strong BrØnsted acid site exhibited high initial
stronger
stronger acid
stronger acid site remains
stronger acid site remains a
stronger acid site remains a challenge
stronger acid site remains a challenge for
strongest
strongest Keggin
strongest Keggin HPA
strongest Keggin HPA ,
strongest Keggin HPA , H3PW12O40
strongest Keggin HPA , H3PW12O40 -LRB-
strongest Keggin HPA , H3PW12O40 -LRB- HPW
studied
studied in
studied in the
studied in the gas
studied in the gas phase
studied in the gas phase at
studied in the gas phase at 200oC
study
study comprise
study comprise the
study comprise the strongest
study comprise the strongest Keggin
study comprise the strongest Keggin HPA
study comprise the strongest Keggin HPA ,
study the
study the acidity
study the acidity and
study the acidity and catalytic
study the acidity and catalytic activity
study the acidity and catalytic activity of
sub-monolayer
sub-monolayer coverage on
sub-monolayer coverage on Nb2O5 ,
sub-monolayer coverage on Nb2O5 , ZrO2
sub-monolayer coverage on Nb2O5 , ZrO2 and
such
such as
such as bulk
such as bulk and
such as bulk and silica-supported
such as bulk and silica-supported H3PW12O40
such as bulk and silica-supported H3PW12O40 and
such as limonene
such as limonene ,
such as limonene , terpinolene
such as limonene , terpinolene ,
such as limonene , terpinolene , terpinene
such as terpene
such composite
such composite at
such composite at a
such composite at a gas-solid
such composite at a gas-solid interface
suffered
suffered from
suffered from catalyst
suffered from catalyst deactivation ,
suffered from catalyst deactivation , resulting
suffered from catalyst deactivation , resulting in
support :
support : SiO2
support : SiO2 <
support : SiO2 < TiO2
support : SiO2 < TiO2 <
support : SiO2 < TiO2 < Nb2O5
support and
support and HPA
support and HPA in
support and HPA in the
support and HPA in the following
support and HPA in the following order
Support from
Support from the
Support from the EPSRC -LRB-
Support from the EPSRC -LRB- research
Support from the EPSRC -LRB- research grant
support increase
support increase in
support increase in the
support increase in the series
support increase in the series of
support increase in the series of support
supported
supported at
supported at sub-monolayer

supported at sub-monolayer coverage on
supported at sub-monolayer coverage on Nb2O5 ,
supported HPW
supported on
supported on Nb2O5
supported on Nb2O5 ,
supported on Nb2O5 , ZrO2
supported on Nb2O5 , ZrO2 and
supported on Nb2O5 , ZrO2 and TiO2
supported on TiO2
supported on TiO2 ,
supported on TiO2 , ZrO2
supported on TiO2 , ZrO2 and
supported on TiO2 , ZrO2 and Nb2O5
supported on TiO2 , ZrO2 and Nb2O5have
supported onto
supported onto SiO2 ,
supported onto SiO2 , Nb2O5
supported onto SiO2 , Nb2O5 ,
supported onto SiO2 , Nb2O5 , ZrO2
terpinene ,
terpinene , β-pinene
terpinene , β-pinene ,
terpinene , β-pinene , p-cymene
terpinene , β-pinene , p-cymene and
terpinene , β-pinene , p-cymene and other
terpinolene ,
terpinolene , terpinene
terpinolene , terpinene ,
terpinolene , terpinene , β-pinene
terpinolene , terpinene , β-pinene ,
terpinolene , terpinene , β-pinene , p-cymene
tested
tested in
tested in isopropanol
tested in isopropanol dehydration
tested in isopropanol dehydration -LRB-
tested in isopropanol dehydration -LRB- 2
tested in isopropanol dehydration -LRB- 2 -RRB-
TGA ,
TGA , XRD
TGA , XRD ,
TGA , XRD , FTIR
TGA , XRD , FTIR and
TGA , XRD , FTIR and 31P{1H}
than
than that
than that of
than that of HPW
than that of HPW and
than that of HPW and Cs2.5H0.5PW12O40
that
that isopropanol
that isopropanol dehydration
that isopropanol dehydration and
that isopropanol dehydration and NH3
that isopropanol dehydration and NH3 adsorption
that isopropanol dehydration and NH3 adsorption calorimetry
that of
that of acidic
that of acidic zeolite
that of HPW
that of HPW and
that of HPW and Cs2.5H0.5PW12O40
that order
that order -LRB-
that order -LRB- 2
that order -LRB- 2 -RRB-
The
The 15%
The 15% HPW/TiO2
The 15% HPW/TiO2 catalyst
The 15% HPW/TiO2 catalyst gave
The 15% HPW/TiO2 catalyst gave a
The 15% HPW/TiO2 catalyst gave a camphene
The acid
The acid strength
The acid strength of
The acid strength of HPA
The acid strength of HPA supported
The acid strength of HPA supported on
The acid strength of these
The acid strength of these catalyst
The acid strength of these catalyst is
the acidity
the acidity and
the acidity and catalytic
the acidity and catalytic activity
the acidity and catalytic activity of
the acidity and catalytic activity of such
The acidity of
The acidity of catalyst
The acidity of catalyst was
The acidity of catalyst was assessed
The acidity of catalyst was assessed by
the acidity of HPA
the acidity of HPA catalyst
the advantage
the advantage of
the advantage of continuous
the advantage of continuous operation
The advantage of these
The advantage of these HPA
The advantage of these HPA catalyst
The advantage of these HPA catalyst over
The aim
The aim of
The aim of this
The aim of this work
The aim of this work is
The aim of this work is to
The catalyst
the catalyst acid
the catalyst acid strength
the catalyst acid strength as
the catalyst acid strength as determined
the catalyst acid strength as determined by
the catalyst acid strength in
the catalyst acid strength in that
the catalyst acid strength in that order
The catalyst comprising
The catalyst comprising HPW
The catalyst comprising HPW -LRB-

The catalyst comprising HPW -LRB- 15
The catalyst comprising HPW -LRB- 15 wt%
the catalyst supported
the catalyst supported on
the catalyst supported on TiO2
the catalyst supported on TiO2 ,
the catalyst supported on TiO2 , ZrO2
The catalyst under
The catalyst under study
The catalyst under study comprise
The catalyst under study comprise the
The catalyst under study comprise the strongest
The catalyst were
The catalyst were tested
The catalyst were tested in
The catalyst were tested in isopropanol
The catalyst were tested in isopropanol dehydration
The catalytic
The catalytic activity
The catalytic activity -LRB-
The catalytic activity -LRB- turnover
The catalytic activity -LRB- turnover frequency
The catalytic activity -LRB- turnover frequency ,
the commercial
the commercial liquid-phase
the commercial liquid-phase batch
the commercial liquid-phase batch process
the commercial liquid-phase batch process ,
the commercial liquid-phase batch process , with
the EPSRC
the EPSRC -LRB-
the EPSRC -LRB- research
the EPSRC -LRB- research grant
the EPSRC -LRB- research grant EP
the EPSRC -LRB- research grant EP /
the following
the following order
the following order of
the following order of support
the following order of support :
the following order of support : SiO2
the gas
the gas phase
the gas phase at
the gas phase at 200oC
the gas phase at 200oC and
the gas phase at 200oC and ambient
the gas phase in
the gas phase in a
the gas phase in a fixed-bed
the gas phase in a fixed-bed reactor
the gas-phase
the gas-phase isopropanol
the gas-phase isopropanol dehydration
the gas-phase isopropanol dehydration was
the gas-phase isopropanol dehydration was found
the gas-phase isopropanol dehydration was found to
The HPA
The HPA catalyst
The HPA catalyst supported
The HPA catalyst supported on
the HPA catalyst supported on Nb2O5
the HPA catalyst supported on Nb2O5 ,
The HPA catalyst supported on TiO2
The HPA catalyst supported on TiO2 ,
The HPA catalyst with
The HPA catalyst with strong
The HPA catalyst with strong BrØnsted
The HPA catalyst with strong BrØnsted acid
the initial
the initial enthalpy
the initial enthalpy of
the initial enthalpy of ammonia
the initial enthalpy of ammonia adsorption
the initial enthalpy of ammonia adsorption for
The isomerisation
The isomerisation of
The isomerisation of α-pinene
The isomerisation of α-pinene was
The isomerisation of α-pinene was studied
The isomerisation of α-pinene was studied in
the latter
the latter mainly
the latter mainly originating
the latter mainly originating from
the latter mainly originating from the
the latter mainly originating from the support
the main
the main product
the main product in
the main product in a
the main product in a mixture
the main product in a mixture with
the parent
the parent H3PW12O40
the parent H3PW12O40 ,
the parent H3PW12O40 , possessing
the parent H3PW12O40 , possessing strong
the parent H3PW12O40 , possessing strong BrØnsted
The reaction
The reaction yielded
The reaction yielded camphene
The reaction yielded camphene as
The reaction yielded camphene as the
The reaction yielded camphene as the main
the series
the series of
the series of support
the series of support :
the series of support : SiO2
the series of support : SiO2 <
the strongest
the strongest Keggin
the strongest Keggin HPA
the strongest Keggin HPA ,
the strongest Keggin HPA , H3PW12O40
the strongest Keggin HPA , H3PW12O40 -LRB-
the support
there
there has
there has been
there has been considerable
there has been considerable research
there has been considerable research activity

there has been considerable research activity ,
There is
There is a
There is a good
There is a good linear
There is a good linear relationship
There is a good linear relationship between
thermally
thermally stable
thermally stable HPA
thermally stable HPA composite possessing
thermally stable HPA composite possessing stronger
thermally stable HPA composite possessing stronger acid
these
these catalyst
these catalyst ,
these catalyst , ΔHi
these catalyst , ΔHi -LRB-
these catalyst , ΔHi -LRB- Figure
these catalyst , ΔHi -LRB- Figure 1
these catalyst is
these catalyst is weaker
these catalyst is weaker than
these catalyst is weaker than that
these catalyst is weaker than that of
these HPA
these HPA catalyst
these HPA catalyst over
these HPA catalyst over zeolite
these HPA catalyst over zeolite is
these HPA catalyst over zeolite is in
these oxide
these oxide with
these oxide with HPW
these oxide with HPW from
these oxide with HPW from aqueous
these oxide with HPW from aqueous solution
this
This shows
This shows that
This shows that isopropanol
This shows that isopropanol dehydration
This shows that isopropanol dehydration and
This shows that isopropanol dehydration and NH3
this work
this work is
this work is to
this work is to study
this work is to study the
this work is to study the acidity
TiO2 ,
TiO2 , although
TiO2 , although weaker
TiO2 , although weaker acid
TiO2 , although weaker acid ,
TiO2 , although weaker acid , showed
TiO2 , ZrO2
TiO2 , ZrO2 and
TiO2 , ZrO2 and Nb2O5
TiO2 , ZrO2 and Nb2O5 were
TiO2 , ZrO2 and Nb2O5 were compared
TiO2 , ZrO2 and Nb2O5have
TiO2 , ZrO2 and Nb2O5have both
TiO2 , ZrO2 and Nb2O5have both BrØnsted
TiO2 <
TiO2 < Nb2O5
TiO2 < Nb2O5 <
TiO2 < Nb2O5 < ZrO2
TiO2 < Nb2O5 < ZrO2 ,
TiO2 < Nb2O5 < ZrO2 , causing
TiO2 < Nb2O5 < ZrO2 , decreasing
TiO2 as
TiO2 as oxide
TiO2 is
TiO2 is similar
TiO2 is similar to
TiO2 is similar to that
TiO2 is similar to that of
TiO2 is similar to that of acidic
TiO2 were
TiO2 were prepared
TiO2 were prepared by
TiO2 were prepared by wet
TiO2 were prepared by wet impregnation
TiO2 were prepared by wet impregnation of
to
to "
to " standard
to " standard ”
to " standard ” HPA
to " standard ” HPA catalyst
to " standard ” HPA catalyst such
to decrease
to decrease in
to decrease in line
to decrease in line with
to decrease in line with the
to decrease in line with the catalyst
to generate
to generate economic
to generate economic reward
to generate economic reward and
to generate economic reward and green
to generate economic reward and green benefit
to study
to study the
to study the acidity
to study the acidity and
to study the acidity and catalytic
to study the acidity and catalytic activity
to that
to that of
to that of acidic
to that of acidic zeolite
to the
to the commercial
to the commercial liquid-phase
to the commercial liquid-phase batch
to the commercial liquid-phase batch process
to the commercial liquid-phase batch process ,
to the parent
to the parent H3PW12O40
to the parent H3PW12O40 ,
to the parent H3PW12O40 , possessing

to the parent H3PW12O40 , possessing strong
TOF -RRB-
TOF -RRB- and
TOF -RRB- and ΔHi
TOF -RRB- and ΔHi for
TOF -RRB- and ΔHi for bulk
TOF -RRB- and ΔHi for bulk and
TOF -RRB- and the
TOF -RRB- and the initial
TOF -RRB- and the initial enthalpy
TOF -RRB- and the initial enthalpy of
TOF -RRB- in
TOF -RRB- in the
TOF -RRB- in the gas-phase
TOF -RRB- in the gas-phase isopropanol
TOF -RRB- in the gas-phase isopropanol dehydration
total
total camphene and
total camphene and limonene
total camphene and limonene yield
total camphene and limonene yield of
total camphene and limonene yield of 58%
tungsten hpa and
tungsten hpa and Nb2O5
tungsten hpa and Nb2O5 ,
tungsten hpa and Nb2O5 , ZrO2
tungsten hpa and Nb2O5 , ZrO2 and
turnover
turnover frequency ,
turnover frequency , TOF
turnover frequency , TOF -RRB-
turnover frequency , TOF -RRB- in
turnover frequency , TOF -RRB- in the
under
under study
under study comprise
under study comprise the
under study comprise the strongest
under study comprise the strongest Keggin
under study comprise the strongest Keggin HPA
was
was assessed
was assessed by
was assessed by DRIFTS
was assessed by DRIFTS of
was assessed by DRIFTS of adsorbed
was assessed by DRIFTS of adsorbed pyridine
was found
was found to
was found to decrease
was found to decrease in
was found to decrease in line
was found to decrease in line with
was studied
was studied in
was studied in the
was studied in the gas
was studied in the gas phase
was studied in the gas phase at
weaker
weaker acid ,
weaker acid , showed
weaker acid , showed more
weaker acid , showed more stable
weaker acid , showed more stable performance
weaker than
weaker than that
weaker than that of
weaker than that of HPW
weaker than that of HPW and
weaker than that of HPW and Cs2.5H0.5PW12O40
were
were compared
were compared to
were compared to "
were compared to " standard
were compared to " standard ”
were compared to " standard ” HPA
were prepared
were prepared by
were prepared by wet
were prepared by wet impregnation
were prepared by wet impregnation of
were prepared by wet impregnation of these
were tested
were tested in
were tested in isopropanol
were tested in isopropanol dehydration
were tested in isopropanol dehydration -LRB-
were tested in isopropanol dehydration -LRB- 2
wet
wet impregnation of
wet impregnation of these
wet impregnation of these oxide
wet impregnation of these oxide with
wet impregnation of these oxide with HPW
which
which is
which is similar
which is similar to
which is similar to the
which is similar to the commercial
which is similar to the commercial liquid-phase
with
with HPW
with HPW from
with HPW from aqueous
with HPW from aqueous solution
with HPW from aqueous solution and
with HPW from aqueous solution and characterised
with monoterpene
with monoterpene by-product
with monoterpene by-product such
with monoterpene by-product such as
with monoterpene by-product such as limonene
with monoterpene by-product such as limonene ,
with on-line
with on-line GC
with on-line GC analysis
with strong
with strong BrØnsted
with strong BrØnsted acid
with strong BrØnsted acid site
with strong BrØnsted acid site exhibited

with strong BrØnsted acid site exhibited high
with the
with the advantage
with the advantage of
with the advantage of continuous
with the advantage of continuous operation
with the catalyst
with the catalyst acid
with the catalyst acid strength
with the catalyst acid strength as
with the catalyst acid strength as determined
with the latter
with the latter mainly
with the latter mainly originating
with the latter mainly originating from
with the latter mainly originating from the
work
work is
work is to
work is to study
work is to study the
work is to study the acidity
work is to study the acidity and
wt%
wt% -RRB-
wt% -RRB- supported
wt% -RRB- supported onto
wt% -RRB- supported onto SiO2
wt% -RRB- supported onto SiO2 ,
wt% -RRB- supported onto SiO2 , Nb2O5
XRD
XRD ,
XRD , FTIR
XRD , FTIR and
XRD , FTIR and 31P{1H}
XRD , FTIR and 31P{1H} MAS
XRD , FTIR and 31P{1H} MAS NMR
year
year ,
year , there
year , there has
year , there has been
year , there has been considerable
year , there has been considerable research
yield
yield of
yield of 51%
yield of 51% and
yield of 51% and a
yield of 51% and a total
yield of 51% and a total camphene
yield of 58%
yield of 58% ,
yield of 58% , which
yield of 58% , which is
yield of 58% , which is similar
yielded
yielded camphene
yielded camphene as
yielded camphene as the
yielded camphene as the main
yielded camphene as the main product
yielded camphene as the main product in
zeolite
zeolite is
zeolite is in
zeolite is in better
zeolite is in better accessibility
zeolite is in better accessibility of
zeolite is in better accessibility of reactant
ZrO2 ,
ZrO2 , causing
ZrO2 , causing partial
ZrO2 , causing partial decomposition
ZrO2 , causing partial decomposition of
ZrO2 , causing partial decomposition of HPA
ZrO2 , decreasing
ZrO2 , decreasing the
ZrO2 , decreasing the catalyst
ZrO2 , decreasing the catalyst acid
ZrO2 , decreasing the catalyst acid strength
ZrO2 and
ZrO2 and Nb2O5
ZrO2 and Nb2O5 were
ZrO2 and Nb2O5 were compared
ZrO2 and Nb2O5 were compared to
ZrO2 and Nb2O5 were compared to "
ZrO2 and Nb2O5have
ZrO2 and Nb2O5have both
ZrO2 and Nb2O5have both BrØnsted
ZrO2 and Nb2O5have both BrØnsted and
ZrO2 and Nb2O5have both BrØnsted and Lewis
ZrO2 and TiO2
ZrO2 and TiO2 ,
ZrO2 and TiO2 , although
ZrO2 and TiO2 , although weaker
ZrO2 and TiO2 , although weaker acid
ZrO2 and TiO2 as
ZrO2 and TiO2 as oxide
ZrO2 and TiO2 as oxide matrix
ZrO2 and TiO2 is
ZrO2 and TiO2 is similar
ZrO2 and TiO2 is similar to
ZrO2 and TiO2 is similar to that
ZrO2 and TiO2 were
ZrO2 and TiO2 were prepared
ZrO2 and TiO2 were prepared by
ZrO2 and TiO2 were prepared by wet
α-pinene isomerisation -LRB-
α-pinene isomerisation -LRB- 3
α-pinene isomerisation -LRB- 3 -RRB-
α-pinene isomerisation -LRB- 3 -RRB- in
α-pinene isomerisation -LRB- 3 -RRB- in the
α-pinene was
α-pinene was studied
α-pinene was studied in
α-pinene was studied in the
α-pinene was studied in the gas
α-pinene was studied in the gas phase
β-pinene ,
β-pinene , p-cymene
β-pinene , p-cymene and
β-pinene , p-cymene and other

## Comparison between automatically selected term-like n-grams and expert selected terms

| Term-like n-grams | Expert selected |
|---|---|
| 31P | |
| 15%HPW/TIO2 | 15%HPW/TIO2 |
| 15%HPW/TIO2 CATALYST | 15%HPW/TiO2 catalyst |
| 31P MAS NMR | 31P MAS NMR |
| 31P{1H} | |
| 31P{1H} MAS NMR | 31P{1H} MAS NMR |
| ACCESSIBILITY | |
| ACCESSIBILITY OF REACTANT MOLECULE | accessibility of reactant molecule |
| ACID | acid |
| ACID CATALYSIS | acid catalysis |
| | acid property |
| ACID SITE | acid site |
| ACID STRENGTH | acid strength |
| ACID STRENGTH AS DETERMINED BY NH3 ADSORPTION | |
| | acid strength of acidic zeolite |
| | acid strength of catalysts |
| | acid strength of Cs2.5H0.5PW12O40 |
| ACID STRENGTH OF HPA SUPPORTED ON NB2O5 | acid strength of HPA supported on Nb2O5 |
| | acid strength of HPA supported on TiO2 |
| | acid strength of HPA supported on ZrO2 |
| | acid strength of HPW |
| ACIDIC COMPOSITE | acidic composite |
| | acidic composites comprising tungsten HPA |
| ACIDIC ZEOLITE | acidic zeolite |
| ACIDITY | acidity |
| ACIDITY OF CATALYST | acidity of catalyst |
| | acidity of composite |
| ACIDITY OF HPA CATALYST | acidity of HPA catalyst |
| ACTIVITY | activity |
| ADSORBED PYRIDINE | adsorbed pyridine |
| ADSORPTION | adsorption |
| ADSORPTION CALORIMETRY | adsorption calorimetry |
| | ambient pressure |
| AMMONIA | ammonia |
| AMMONIA ADSORPTION CALORIMETRY | ammonia adsorption calorimetry |
| AQUEOUS SOLUTION | aqueous solution |
| BATCH PROCESS | |
| BET | BET |
| | better accessibility of reactant molecules |
| BRØNSTED ACID | brønsted acid |
| BRØNSTED ACID SITE | brønsted acid site |
| | bulk H3PW12O40 |
| | bulk Cs2.5H0.5PW12O40 |
| BY-PRODUCT | |
| CAMPHENE | camphene |
| | camphene as a main product |
| CAMPHENE YIELD | camphene yield |
| CATALYSIS | |

| | |
|---|---|
| CATALYSIS BY HETEROPOLY ACID | |
| | catalyst |
| CATALYST ACID | |
| CATALYST ACID STRENGTH | catalyst acid strength |
| CATALYST ACID STRENGTH AS DETERMINED BY NH3 | |
| CATALYST CHARACTERISATION | catalyst characterisation |
| CATALYST DEACTIVATION | catalyst deactivation |
| CATALYST OVER ZEOLITE | |
| | catalyst comprising HPW supported onto SiO2 |
| | catalyst comprising HPW supported onto Nb2O5 |
| | catalyst comprising HPW supported onto ZrO2 |
| | catalyst comprising HPW supported ontoTiO2 |
| | catalyst supported |
| CATALYST SUPPORTED ON NB2O5 | catalyst supported on Nb2O5 |
| CATALYST SUPPORTED ON TIO2 | catalyst supported on TiO2 |
| | catalyst supported on ZrO2 |
| CATALYST WITH STRONG BRØNSTED ACID SITE | catalyst with strong brønsted acid site |
| CATALYTIC ACTIVITY | catalytic activity |
| | catalytic activity of composites |
| | catalytic properties |
| | catalytic properties at a gas-solid interface |
| COMMERCIAL LIQUID-PHASE BATCH PROCESS | commercial liquid-phase batch process |
| COMPOSITE | |
| COMPOSITE AT A GAS-SOLID INTERFACE | |
| CONTINUOUS OPERATION | continuous operation |
| COVERAGE | |
| COVERAGE ON NB2O5 | |
| CS2.5H0.5PW12O40 | Cs2.5H0.5PW12O40 |
| DEACTIVATION | |
| DECOMPOSITION | |
| DECOMPOSITION OF HPA | decomposition of HPA |
| | decreasing the catalyst acid strength |
| | development of thermally stable HPA composites |
| | DRIFTS |
| DRIFTS OF ADSORBED PYRIDINE | DRIFTS of adsorbed pyridine |
| ECONOMIC REWARD | |
| ENTHALPY OF AMMONIA ADSORPTION | enthalpy of ammonia adsorption |
| EP | |
| EPSRC | |
| FIXED-BED REACTOR | fixed-bed reactor |
| FIXED-BED REACTOR WITH ON-LINE GC ANALYSIS | |
| FTIR | FTIR |
| GAS PHASE | gas phase |
| GAS PHASE IN A FIXED-BED REACTOR | |
| GAS-PHASE | |
| GAS-PHASE ISOPROPANOL DEHYDRATION | gas-phase isopropanol dehydration |
| GAS-SOLID INTERFACE | gas-solid interface |
| GC ANALYSIS | GC analysis |
| H3PW12O40 | H3PW12O40 |
| | H3PW12O40 supported at sub-monolayer coverage |
| | H3PW12O40 heteropoly acid |
| | H3PW12O40 supported at sub-monolayer coverage on |

| | |
|---|---|
| | Nb2O5 |
| | H3PW12O40 supported at sub-monolayer coverage on TiO2 |
| | H3PW12O40 supported at sub-monolayer coverage on ZrO2 |
| HETEROGENEOUS ACID | |
| HETEROGENEOUS ACID CATALYSIS | heterogeneous acid catalysis |
| | heterogeneous acid catalysis by heteropoly acids |
| HETEROPOLY ACID | heteropoly acid |
| HPA | HPA |
| HPA CATALYST | HPA catalyst |
| HPA CATALYST OVER ZEOLITE | HPA catalyst over zeolite |
| HPA CATALYST SUPPORTED ON NB2O5 | HPA catalyst supported on Nb2O5 |
| | HPA catalyst supported on ZrO2 |
| HPA CATALYST SUPPORTED ON TIO2 | HPA catalyst supported on TiO2 |
| HPA CATALYST WITH STRONG BRØNSTED ACID SITE | HPA catalyst with strong brønsted acid site |
| HPA COMPOSITE | HPA composite |
| HPA SUPPORTED ON NB2O5 | HPA supported on Nb2O5 |
| | HPA supported on TiO2 |
| | HPA supported on ZrO2 |
| HPW | HPW |
| | HPW (15 wt%) supported onto Nb2O5 |
| | HPW (15 wt%) supported onto SiO2 |
| | HPW (15 wt%) supported onto TiO2 |
| | HPW (15 wt%) supported onto ZrO2 |
| HPW CATALYST | HPW catalyst |
| HPW FROM AQUEOUS SOLUTION | |
| | HPW supported at sub-monolayer coverage on Nb2O5 |
| | HPW supported at sub-monolayer coverage on TiO2 |
| | HPW supported at sub-monolayer coverage on ZrO2 |
| IMPREGNATION | |
| INITIAL ACTIVITY | initial activity |
| INITIAL ENTHALPY | initial enthalpy |
| INITIAL ENTHALPY OF AMMONIA ADSORPTION | initial enthalpy of ammonia adsorption |
| INITIAL HIGH ACTIVITY | |
| INTERACTION BETWEEN HPW | |
| | interaction between HPW and support |
| INTERACTION BETWEEN THE SUPPORT | |
| ISOMERISATION | isomerisation |
| ISOMERISATION OF α-pinene | isomerisation of α-pinene |
| ISOPROPANOL | isopropanol |
| ISOPROPANOL DEHYDRATION | isopropanol dehydration |
| KEGGIN HPA | Keggin HPA |
| LARGE ORGANIC MOLECULE | large organic molecule |
| LEWIS | |
| LEWIS ACID | Lewis acid |
| LEWIS ACID SITE | Lewis acid site |
| LIMONENE | limonene |
| LIMONENE YIELD | limonene yield |
| LINE WITH THE CATALYST ACID STRENGTH | |
| LINEAR RELATIONSHIP | linear relationship |
| LINEAR RELATIONSHIP BETWEEN LOG | |
| LIQUID-PHASE | liquid-phase |

| | |
|---|---|
| LIQUID-PHASE BATCH | |
| LIQUID-PHASE BATCH PROCESS | liquid-phase batch process |
| LOW CAMPHENE YIELD | |
| MAS NMR | MAS NMR |
| MIXTURE WITH MONOTERPENE BY-PRODUCT | |
| MONOTERPENE | monoterpene |
| MONOTERPENE BY-PRODUCT | |
| | monoterpene by-products |
| NB2O5 | Nb2O5 |
| | Nb2O5 as oxide matrix |
| NB2O5 HAVE | |
| NH3 | NH3 |
| NH3 ADSORPTION CALORIMETRY | NH3 adsorption calorimetry |
| NMR | |
| ON-LINE GC ANALYSIS | on-line GC analysis |
| ORDER OF SUPPORT | |
| ORGANIC MOLECULE | organic molecule |
| ORIGINATING FROM THE SUPPORT | |
| OXIDE | |
| OXIDE MATRIX | oxide matrix |
| OXIDE WITH HPW | |
| OXIDE WITH HPW FROM AQUEOUS SOLUTION | |
| PARTIAL DECOMPOSITION | partial decomposition |
| PARTIAL DECOMPOSITION OF HPA | partial decomposition of HPA |
| P-CYMENE | p-cymene |
| PHASE IN A FIXED-BED REACTOR | |
| PYRIDINE | pyridine |
| REACTANT MOLECULE | reactant molecule |
| REACTION INVOLVING | |
| REACTION INVOLVING LARGE ORGANIC MOLECULE | reaction involving large organic molecule |
| REACTOR | |
| REACTOR WITH ON-LINE GC ANALYSIS | |
| RELATIONSHIP BETWEEN LOG | |
| | silica-supported Cs2.5H0.5PW12O40 |
| SILICA-SUPPORTED H3PW12O40 | silica-supported H3PW12O40 |
| SIO2 | SiO2 |
| | solid acid |
| | solid acid catalysts |
| | solid acid catalysts based on H3PW12O40 |
| | solid acid catalysts based on H3PW12O40 heteropoly acid |
| STABLE HPA COMPOSITE | stable HPA composite |
| | standart HPA catalyst |
| STRENGTH AS DETERMINED BY NH3 ADSORPTION CALORIMETRY | |
| STRENGTH OF HPA SUPPORTED ON NB2O5 | |
| STRONG BRØNSTED ACID | |
| STRONG BRØNSTED ACID SITE | strong brønsted acid site |
| STRONGER ACID SITE | stronger acid site |
| | strongest Keggin HPA |
| | sub-monolayer |
| SUB-MONOLAYER COVERAGE | sub-monolayer coverage |
| SUB-MONOLAYER COVERAGE ON NB2O5 | sub-monolayer coverage on Nb2O5 |
| SUFFERED FROM CATALYST DEACTIVATION | |

| | |
|---|---|
| SUPPORT | support |
| SUPPORT FROM THE EPSRC | |
| SUPPORTED AT SUB-MONOLAYER COVERAGE | |
| SUPPORTED AT SUB-MONOLAYER COVERAGE ON NB2O5 | |
| SUPPORTED HPW CATALYST | |
| SUPPORTED ONTO SIO2 | |
| TERPENE | terpene |
| TERPINENE | terpinene |
| TERPINOLENE | terpinolene |
| TGA | TGA |
| THERMALLY STABLE HPA COMPOSITE | thermally stable HPA composite |
| TIO2 | TiO2 |
| TIO2 AS OXIDE MATRIX | TIO2 as oxide matrix |
| TOF | TOF |
| TOTAL CAMPHENE | |
| TUNGSTEN | tungsten |
| TUNGSTEN HPA | tungsten HPA |
| TURNOVER FREQUENCY | turnover frequency |
| WEAKER ACID | |
| WET IMPREGNATION | wet impregnation |
| | XRD |
| | zeolite |
| ZRO2 | ZrO2 |
| | ZrO2 as oxide matrix |
| α-PINENE | α-pinene |
| α-PINENE ISOMERISATION | α-pinene isomerisation |
| β-PINENE | β-pinene |
