# Supplementary material for: Terminology spectrum analysis of natural-language chemical documents: term-like phrases retrieval routine
Source: J Cheminform. 2016 Apr 29;8:22. doi: 10.1186/s13321-016-0136-4 (PMC4850643; doi:10.1186/s13321-016-0136-4)
Supplement: Supplementary file 2 — 10.1186/s13321-016-0136-4 OSCAR4 tokenizer modification. [file 13321_2016_136_MOESM2_ESM.pdf]

## Additional file 2
## OSCAR4 tokenizer modification

```
int splittableHyphenIndex = HyphenTokeniser.indexOfSplittableHyphen(tokenSurface);

            if (splittableHyphenIndex != -1
                && !tokenSurface.matches(".*[a-z][a-z].*")
                && tokenSurface.matches(".*[A-Z].*")) {
              //FIXME dmj30 I don't see the point of the two String.matches calls above
              if (tokenClassifier.isTokenLevelRegexMatch(tokenSurface, "bondRegex")) {
                splittableHyphenIndex = -1;
              }
           }
           if (splittableHyphenIndex != -1) {
              if (tokenSurface.endsWith("NMR")) {
                return splitAt(token, token.getStart() + splittableHyphenIndex,
                        token.getStart() + splittableHyphenIndex + 1);
              } else if (prefixPattern.matcher(tokenSurface).matches()) {
                return splitAt(token, token.getStart() + splittableHyphenIndex + 1);

              } else {
                ///This is where tokenisation happens
                return splitAt(token, token.getStart() + splittableHyphenIndex,
                        token.getStart() + splittableHyphenIndex + 1);
              }
           } else {
              return null;
           }
```
