# Supplementary material for: Terminology spectrum analysis of natural-language chemical documents: term-like phrases retrieval routine
Source: J Cheminform. 2016 Apr 29;8:22. doi: 10.1186/s13321-016-0136-4 (PMC4850643; doi:10.1186/s13321-016-0136-4)
Supplement: Supplementary file 3 — 10.1186/s13321-016-0136-4 List of excluded words from general English Corncob- Lowercase list. [file 13321_2016_136_MOESM3_ESM.pdf]

Additional file 3

List of excluded words from general English Corncob- Lowercase list

aberration
aberrations
ablation
ablution
ablutions
about
above
abrasion
abrasions
abrasive
abrasively
abrasiveness
abrasives
abrupt
absorbed
absorbency
absorbent
absorber
absorbers
absorption
absorptions
absorptive
absorptivity
abundance
abundances
abundant
accelerated
acceleration
accelerations
accelerator
accelerators
acceptability
acceptor
acceptors
accessibility
accommodation
accommodations
accommodated
acetal
acetate
acetates
acetic
acetone
acetylene
acid
acidic
acidification
acidified
acidify
acidifying
acidity
acids
acrylic
acrylics
across
act
acted
acting
actings
actinides
action
activate
activated
activates
activating
activation
activations
activator
activators
active
activities
activity
acyclic
adding
addition
additions
additive
additives
adhesion
adhesions
adhesive
adhesiveness
adhesives
adiabatic
adsorbed
adsorption
aerate
aerated
aerates
aerating
aeration
aerator
aerodynamic
aerodynamically
aerodynamics
aerosol
aerosols
age
aged
ageing
ageings
ages
aging
agings
agglomerated
agglomerating
agglomeration
agglomerations
aggregate
aggregated
aggregates
aggregating
aggregation
aggregations
alanine
alcohol
alcohols
aldehyde
aldehydes
algorithm
algorithms
aliphatic
alkali
alkalic
alkaline
alkalinity
alkaloid
alkaloids
alkanes
alkyl
allied
allotrope
allotropic
allowed
alloy

alloyed
alloying
alloys
aluminium
aluminum
amethyst
amide
amine
amines
ammonia
ammonites
ammonium
anchoring
aniline
annealing
antacid
anthracite
antioxidants
aromatics
aromaticity
asbestos
ash
attrition
barium
basalt
bauxite
bead
beads
benzene
biomass
bismuth
bitumen
bifurcation
bifurcations
binder
binders
bleaching
bonding
cadmium
calcium
calorimetry
catalysis
catalyst
catalysts
carbide
carbine
carbines
carbohydrate
carbohydrates
carbon
carbons
carbonate
carbonates
carborundum
carrier
carriers
ceramic
ceramics
channel
channels
charcoal
cholesterol
chromium
chlorine
chlorination
chloroform
chlorofluorocarbon
chlorofluorocarbons
citrate
citrates
centrifugation
clay
clays
cluster
clusters
clustering
coagulation
coal
coals
coalblack
coalescence
coating
cobalt
cohesion
coke
colloids
conversion
composite
composites
combustion
complex
complexes
conduction
copper
corrosion
cosy
cotton
cottons
coverage
coverages
coupling
cracking
crushing
crystal
crystals
cutting
deactivation
density
desorption
diamond
diamonds
diffraction
diffusivity
deposition
diffusion
distillation
distillations
descriptor
descriptors
dispersion
dissolution
detergent
detergents
dioxin
dioxins
dolomite
doping
drifts
drying
elimination
eliminations
embedding
emission
emissions
enzyme
enzymes
erosion
ester
esters
ethane
ethanol
ethylene
extrusion
fat
fats
ferrite
fluorescence

fructose
fuel
fuels
gallium
germanium
glycerine
glycerol
glycine
glycol
glucose
gold
graphite
grafting
granule
granules
granulation
grinding
hardness
hardening
hexane
heating
heptane
herbicide
herbicides
holdup
honeycomb
hydration
hydrazine
hydrocarbon
hydrocarbons
hydrogen
hydrogenation
hydrolysis
hydroxide
hydroxides
hysteresis
immersion
inhibitor
inhibition
inhibitions
inhibitors
insulator
intermediate
intermediates
iodide
iodine
iridium
iron
isotope
isotopes
kaolin
kneading
lactose
leaching
lignite
liquefaction
lipase
lithium
lubricant
lubricants
lysine
magnesium
magnesia
magnetite
magnetron
malachite
maturation
membrane
membranes
mercury
methane
methanol
micelles
migration
migrations
mineral
minerals
mineralisation
molybdenum
monolayer
monolayers
nickel
nitrogen
nylon
nylons
octane
octanes
oscillation
oscillations
overheating
oxalate
oxidation
oxygen
ozone
palladium
pellet
pellets
petroleum
plugging
pharmaceutical
pharmaceuticals
phenol
phenols
phenylalanine
phonon
phosphor
photochemistry
photolysis
photosynthesis
pillar
platinum
poison
poisons
poisoning
pollutant
pollutants
polycarbonate
polyester
polyesters
polyethylene
polymer
polymerase
polymerases
polymerisation
polymers
polypeptide
polypeptides
polypropylene
polysaccharide
polysaccharides
polystyrene
polyurethane
porosity
positron
positrons
precursor
precursors
precipitation
promoter
promoters
propagator
propagators
propane
propylene
pyridine
pyrite
pyrites

pyrolysis
pyroxene
pyroxenes
quartz
quartzite
quencher
quenchers
quenching
radiation
radiations
radical
radicals
radioactivity
radiocarbon
reach
reactor
reactors
reactivity
rearrangement
rearrangements
receptor
receptors
reconstruction
reconstructions
recombination
rectification
recycling
redistribution
redistributions
reducibility
reduction
reflectance
reflection
reforming
refraction
regeneration
regenerations
regression
regressions
relaxation
relaxations
resin
resins
resolution
rhenium
rhodium
sampling
saturation
scattering
screening
sedimentation
seeding
segregation
selectivity
semiconductor
semiconductors
sensitisation
sensitisers
sensitivity
silica
silver
silicone
sieving
solubility
specificity
spectator
spectators
spectroscopy
spreading
sputtering
stability
starch
steaming
sticking
stress
strontium
styrene
substitution
substrate
substrates
sucrose
surface
surfaces
sugar
sugars
sulphate
sulphates
sulphide
sulphides
sulphonamides
sulphur
superconductivity
superconductor
superconductors
supersaturation
support
supports
surface
surfactant
surfactants
symbiosis
synergism
swelling
swellings
talc
tame
tantalum
tap
tar
tars
template
templates
texture
textures
tin
titanium
titration
toluene
ton
transmigration
transmittance
trapping
trappings
triglyceride
tungsten
vanadium
viscosity
vitamin
vitamins
void
voids
waste
wealth
wetting
xray
zeolite
zeolites
