# Supplementary material for: Terminology spectrum analysis of natural-language chemical documents: term-like phrases retrieval routine
Source: J Cheminform. 2016 Apr 29;8:22. doi: 10.1186/s13321-016-0136-4 (PMC4850643; doi:10.1186/s13321-016-0136-4)
Supplement: Supplementary file 4 — 10.1186/s13321-016-0136-4 List of stop words used. [file 13321_2016_136_MOESM4_ESM.pdf]

Additional file 4
List of stop words used

a,b
abbrev
abbreviated
abbreviates
abbreviating
abbreviation
abbreviations
abc
abet
about
above
abovementioned
above-mentioned
abundant
academe
acadimia
academic
academical
academically
academician
academicians
academics
academies
academy
accuracies
accuracy
accurate
accurately
accordance
according
accordingly
account
achieved
achievement
achievements
achieves
achieving
acknowledge
acknowledged
acknowledgement
acknowledgements
acknowledges
acknowledgment
acknowledgments
acquired
acquirer
acquirers
acquiring
acquisition
acquisitions
acquisitive
acquisitiveness
actual
actualisation
actualised
actuality
actually
adaptability
adaptation
adaptations
adapted
adapter
adapters
adaptor
adaptors
additional
additionally
additively
adding
address
addressability
addressable
addressed
addressee
addressees
addresses
addressing
adjusted
adjusting
adjustment
adjustments
administer
administered
administering
administers
administrated
administrating
administration
administrations
administrative
administratively
administrator
administrators
adopted
adopter
adopting
adoption
adoptions
adoptive
advance
advanced
advancement
advancements
advancer
advances

advancing
advantage
advantaged
advantageous
advantageously
advantages
affect
affected
affectedly
affecting
affection
affections
affective
affects
afford
affordability
affordable
afforded
affording
affords
aforementioned
aforesaid
africa
afterward
afterwards
age
agencies
agency
ages
again
against
agency
agree
agreeable
agreeableness
agreeably
agreed
agreeing
agreement
agreements
agrees
aid
aides
aiding
aim
aimed
aimer
aiming
aimless
aimlessly
aimlessness
aims
al.
all
allowing
allows
almost
already
also
altered
alternate
alternated
alternately
alternates
alternating
alternation
alternations
alternative
alternatively
alternatives
although
altogether
always
ambiance
ambient
ambiguities
ambiguity
ambiguous
ambiguously
america
amount
amounted
amounting
amounts
analogously
animated
annual
another
answer
answerable
answered
answerer
answering
answers
any
anybody
anyhow
anymore
anyone
anyplace
anything
anyway
anyways
anywhere
apparently
appear
appearance
appearances
appeared
appearing
appears
applied
appreciable
appreciably

appreciate
appreciated
appreciates
appreciating
appreciation
appreciations
appreciative
appreciatively
approachability
approachable
approached
approaching
appropriate
appropriated
appropriately
appropriateness
appropriates
appropriating
appropriation
appropriations
approximated
approximately
approximates
approximating
approximation
approximations
aq
aq.
argument
argumentation
argumentative
argumentatively
arguments
arrival
arrived
arriver
arrives
arriving
article
articles
ascribed
ascribes
ascribing
ascription
ascriptions
asked
asking
aspired
aspires
aspect
aspects
assessment
assessments
assign
assigned
assignment
assignments
assigns
assumed
assumes
assuming
asymptotically
attain
attainable
attained
attaining
attainment
attainments
attempt
attempted
attempting
attempts
attention
attentions
attract
attracted
attracting
attraction
attractions
attractive
attractively
attractiveness
attractor
attractors
attracts
attributable
attribute
attributed
attributes
attributing
attribution
attributions
attributive
atypical
atypically
august
author
authored
authoress
authorial
authoring
authorisation
authorisations
authorise
authorised
authorises
authorising
authors
author
availabilities
availability
available
avoid
avoidable

avoidance
avoided
avoiding
avoids
award
axis
bar
basis
because
become
becomes
becoming
been
being
before
beforehand
begin
beginner
beginners
beginning
beginnings
begins
behaved
believability
believable
believably
believe
believed
believer
believers
believes
believing
benefice
beneficence
beneficent
beneficial
beneficially
beneficiaries
beneficiary
benefit
benefited
benefiting
benefits
better
bettered
bettering
betterment
betters
beyond
book
books
brief
briefcase
briefcases
briefed
briefer
briefest
briefing
briefings
briefly
briefs
brucker
bruker
bulgaria
business
businesses
businesslike
businessman
businessmen
businesswoman
ca.
called
candidate
candidates
candidature
candidatures
cannot
capabilities
capability
capable
capably
capital
careful
carefully
carefulness
careless
carelessly
carelessness
case
cased
cases
cause
caused
causes
cc
certain
certainly
certainties
certainty
certifiable
certifiably
certificate
certificated
certificates
certification
certified
certifies
certify
certifying
challenge
challenged
challenger
challengers
challenges

challenging
challengingly
change
changeability
changeable
changed
changeless
changer
changers
changes
changing
cheap
cheapen
cheapened
cheapening
cheapens
cheaper
cheapest
check
checked
checker
checkered
checkering
checkers
checking
checklist
checklists
chemist
chemists
chief
chiefs
china
chinese
choice
choices
choicest
chose
chosen
claim
claimable
claimant
claimants
claimed
claiming
claims
clarification
clarifications
clarified
clarifies
clarify
clarifying
clearly
close
closely
cm
co-exist
coexist
coexisted
coexistence
coexistent
coexisting
coexists
coextensive
coincide
coincided
coincidence
coincidences
coincident
coincidental
coincidentally
coincides
coinciding
coming
comings
comment
commentaries
commentary
commentate
commentating
commentator
commentators
commented
commenter
commenting
comments
commercially
commission
commissions
common
commonly
communicant
communicants
communicate
communicated
communicates
communicating
communication
communications
communicative
communicativeness
communicator
communicators
community
communities
community's
companies
company
comparable
comparably
comparative
compare
compared
comparing
comparison

comparisons
compartment
compartmentalisation
compartmentalised
compartmentalising
compartments
compatibilities
compatibility
comprise
comprised
comprises
comprising
compromise
compromised
compromises
compromising
computer
concern
concerned
concerning
concerns
conclude
concluded
concludes
conclusion
conclusions
conclusive
conclusively
confer
conference
conferences
conferencing
conferment
conferred
conferring
confers
confidence
confidences
confident
confidential
confidentiality
confidentially
confidently
confirm
confirmation
confirmations
confirmatory
confirmed
confirming
confirms
congress
congresses
conscious
consciously
consciousness
consciousnesses
consider
considerable
considerably
considerate
considerately
considered
considering
considers
consensus
consequently
consumed
consuming
consumption
consumptions
context
contexts
contextual
contextualisation
contextually
contract
contracts
contribute
contributed
contributes
contributing
contribution
contributions
contributor
contributors
contributory
control
conv
convincingly
correctness
correspond
corresponded
correspondence
correspondences
correspondent
correspondents
corresponding
correspondingly
corresponds
corroborate
corroborated
corroborates
corroborating
corroboration
corroborative
corroboratory
cost
council
coworker
coworkers
cowriter
cowritten
currently
day

days
de
deal
dealing
dealings
deals
decade
decades
deduction
deductions
default
defaulted
defaulter
defaulters
defaulting
defaults
defined
definite
definitely
demand
demanded
demanding
demands
demonstrate
demonstrated
demonstrates
demonstrating
demonstration
demonstrations
demonstrative
demonstratively
demonstratives
demonstrator
demonstrators
denotation
denotational
denotations
denote
denoted
denotes
denoting
department
departmental
departmentally
departments
depended
depending
described
describing
description
desirable
desired
despite
detail
detailing
details
detected
detectability
detectable
develop
developed
developer
developers
developing
development
developmental
developmentally
developments
develops
didn't
different
differently
differing
difference
differences
difficult
difficulties
difficulty
directly
disagree
disagreeable
disagreeably
disagreed
disagreeing
disagreement
disagreements
disagrees
disappear
disappearance
disappearances
disappeared
disappearing
disappears
discovered
discuss
discussable
discussed
discusses
discussing
discussion
discussions
display
displayable
displayed
displaying
displays
distinct
doesn't
document
documentaries
documentary
documentation
documented
documenting

documents
done
don't
drastic
drastically
dt
each
ead
earlier
earliest
early
easier
easiest
easily
east
easy
economical
economically
education
educational
educations
effectively
efficient
efficiently
effort
efforts
e.g.
else
elsewhere
employ
employable
employed
employer
employers
employing
employment
employments
employs
e-mail
enable
enabled
enables
enabling
enclose
enclosed
encloses
enclosing
inevitable
engineering
enough
enormous
enormously
entitle
entitled
entitlement
entitlements
entitles
entitling
eq
eq.
eq.1
equipped
error
errors
especial
especially
essential
essentially
establish
established
establishes
establishing
establishment
establishments
et
etc.
europa
european
evaluated
even
eventual
eventualities
eventuality
eventually
ever
every
everybody
everyday
everyone
everything
everywhere
evidence
evidenced
evidences
evident
evidential
evidently
exam
examinable
examination
examinations
examine
examined
examinees
examiner
examiners
examines
examining
example
examples
exams
excelled
excellence
excellencies

excellency
excellent
excellently
excelling
except
excepted
excepting
exception
exceptionable
exceptional
exceptionally
exceptions
excepts
exclude
excluded
excludes
excluding
exclusion
exclusionary
exclusions
exclusive
exclusively
exclusiveness
exclusivist
exclusivity
exhibit
exhibited
exhibiting
exhibition
exhibitions
existence
existences
existing
exists
expected
expecting
expense
expenses
expensive
expensively
experimental
experimentally
experimentation
explain
explainable
explained
explaining
explains
explanation
explanations
exploited
exploiting
explored
exploring
extending
extension
extensions
extensive
extensively
extrema
extremal
extreme
extremely
e.e
fact
factory
facts
fairly
false
faster
fastest
favor
favorable
favored
favoring
favoured
favourable
favouring
favours
feature
featured
featureless
features
featuring
federal
fellowship
few
fewer
fewest
fewness
fig
fig.
fig.1
fig.2
fig.3
fig.4
fig.5
fig.6
fig.7
fig.8
fig.9
fig.1(a)
fig.1(b)
fig.1(c)
fig.1(d)
fig.1,b
fig.1.a
fig.1-2
fig.1.a
fig.1.b
fig.1a
fig.1b
fig.1c
fig.1d

fig.2(a)
fig.2(b)
fig.2(c)
fig.2-b
fig.2a
fig.2b
fig.2c
fig.1show
fig.1shows
fig.2show
fig.2shows
fig.3shows
fig.4shows
fig.a
fig.b
fig1
fig2
fig3
figure
figure1
figure.1
figure.2
figure1b
figure2
finally
finance
financed
finances
financial
financially
financing
finish
finished
firstly
focused
focusing
focussing
followed
following
font
formed
forward
found
founds
foundation
foundations
frequently
fundamental
fundamentally
fundamentals
further
furthermore
future
g
gain
gained
gainer
gainers
gainful
gainfully
gaining
gainly
gains
gave
general
generalitat
generally
germany
getting
given
giving
good
goods
govern
governance
governed
governess
governesses
governing
government
governmental
governments
gram
grams
grant
granted
grantee
granting
grants
great
greater
greatest
greatly
greatness
guarantee
guaranteed
guaranteeing
guarantees
h3
had
hand
handbook
handbooks
handling
happen
happened
happening
happenings
happens
hardly
having
heading
headings
help

helped
helping
helps
hence
here
herein
hereinafter
hh
hierarchically
highest
highlighted
highlighting
highlights
hour
hours
however
huge
human
humans
i.d.
idea
identified
identity
i.e
i.e.
illustrated
illustrating
illustration
illustrations
illustrative
implied
implying
immediate
immediately
importance
important
improved
improving
included
including
increment
increase
increased
increases
increasing
increasingly
indeed
indicated
indicates
indicating
indication
indications
indirectly
initially
initiating
inferred
information
ing
inherent
inherently
innovated
innovating
innovation
innovations
innovative
innovatively
insight
insignificance
insignificant
insignificantly
inspected
inspecting
inspection
inspections
instance
instanced
instances
instant
instantly
instants
instead
istituto
instrument
instruments
insufficient
insured
interest
interested
interestedly
interesting
interestingly
interests
international
interpreted
interpretation
interpretations
introduced
introducing
introduction
introductions
institute
institutes
investigated
investigating
it's
january
just
justified
justifies
justify
justifying
key
keys
keyword

keywords
kind
kinds
kindly
knowing
knowledge
known
l
l1
l2
l3
last
latter
less
like
likely
literature
literatures
longer
lower
lowercase
lowered
lowering
ltd
m
magnitude
magnitudes
main
mainly
maintain
maintained
maintaining
maintains
major
majority
majors
manifest
manifested
manifesting
manifests
many
markedly
market
matched
matching
max
maximal
maybe
micromole
min
minimum
ministerio
ministry
minute
meaning
meanings
means
meanwhile
mentioned
mentioning
mmol
minor
minorities
minority
mm
modern
mole
moles
more
moreover
most
mostly
motivated
motivation
motivations
mpa
much
nº
name
named
namely
names
narrow
narrowed
narrower
narrowest
narrowing
narrowly
narrows
national
nature
nearly
necessarily
necessary
necessities
necessity
need
needed
needs
neglected
neglecting
neglects
negligible
never
nevertheless
new
newer
newest
newly
nicely
night
nitr
nm
noise

noiseless
noiselessly
noises
noisier
noisiest
normally
north
northwest
not
notable
notables
notably
note
noted
notes
noteworthy
nothing
notice
noticeable
noticeably
noticed
notices
noticing
novel
now
nowadays
nowhere
numerous
objective
observable
observed
observing
observation
observations
occur
occurred
occurrence
occurrences
occurring
occurs
often
once
only
ooo
opinion
opinions
originally
organization
other
others
otherwise
overview
our
outlined
outlet
outlets
owing
own
p1
p2
page
pages
paper
papers
paragraph
paragraphs
parent
part
particularly
patent
patents
peculiar
peculiarities
peculiarity
peculiarly
per
percent
percentage
percentages
performed
performance
performing
performs
personal
personally
perspective
perspectives
ph.d
pic
picture
pictured
pictures
pixel
pixels
pka
planet
planet's
play
played
playing
plays
pleased
plenty
plotted
plotting
pointed
pointing
poor
poorer
poorest
poorly
popular
popularisation
popularisations

popularise
popularised
popularising
popularity
popularly
possess
possessed
possesses
possessing
possible
possibles
possibly
postulate
postulated
postulates
postulating
postulation
practically
practice
practices
predominance
predominant
predominantly
predominate
predominated
predominates
predominating
prefer
preferable
preferably
preference
preferences
preferential
preferentially
preferment
preferred
preferring
prefers
president's
present
presented
presenting
presentation
presentational
presentations
presenting
presently
presents
previous
previously
precise
precisely
preferably
preferred
preference
preferentially
preliminary
price
primarily
principal
principally
probable
probably
proceed
proceeded
proceeding
proceedings
proceeds
process's
product
products
project
projects
programme
prominent
prominently
promise
promised
promises
promising
promisingly
pronoun
pronounce
pronounceable
pronounced
pronouncedly
pronouncement
pronouncements
pronounces
pronouncing
pronouns
proposal
proposals
propose
proposed
proposer
proposers
proposes
proposing
prospective
proved
provided
providing
proving
polish
publication
publications
publish
publishable
published
publisher
publishers
publishes
publishing

purpose
purposed
purposeful
purposefully
purposefulness
purposeless
purposelessly
purposely
purposes
purposing
purposive
questing
question
questionable
questionably
questioned
questioner
questioners
questioning
questioningly
questionings
questionnaire
questionnaires
questions
quests
quite
r0
r1
r2
r3
randomly
range
ranged
ranger
rangers
ranges
ranging
rather
reaching
readily
real
realisation
realisations
realise
realised
realises
realising
realism
realist
realistic
realistically
realists
realized
really
reason
reasonable
reasonableness
reasonably
reasoned
reasoner
reasoners
reasoning
reasonless
reasons
received
receiving
recent
recently
recognise
recognised
recogniser
recognisers
recognises
recognising
recognized
recorded
recommended
recommending
recommends
reconsider
reconsideration
reconsidered
reconsidering
reconsiders
ref
ref.
reference
referenced
referencer
references
referencing
refereeing
referees
referred
regard
regarded
regarding
regardless
regards
relatively
release
released
releases
releasing
relevant
relevantly
reliabilities
reliability
remark
remarkable
remarkably
remarked
remarking
remarks

repeated
repeatedly
repeater
repeaters
repeating
repeats
report
reported
reporting
reports
represent
representable
representation
representational
representations
representative
representativeness
representatives
represented
representing
represents
republic
require
required
requiring
requirement
requirements
requires
research
researcher
researchers
researches
researching
respect
respectability
respectable
respectably
respected
respectful
respectfully
respecting
respective
respectively
respects
result
resultant
results
result/discussion
resulted
resulting
return
returnable
returned
returnees
returning
returns
reveal
revealable
revealed
revealing
revealingly
reveals
review
reviewed
reviewing
reviews
right
role
roles
room
root
roots
rough
roughly
run
running
russia
russian
same
sample
sampled
samples
satisfactory
satisfy
satisfying
saying
sbet
scarcely
scarcities
scarcity
schematic
schematically
schematics
scheme
schemes
scheme-1
scheme-2
scheme1
scheme2
scheming
science
sciences
scientific
scientifically
scientist
scientists
school
search
searched
searcher
searchers
searches
searching
seconds

seem
seemed
seems
seen
sense
series
served
serving
servings
session
sessions
set
sets
several
severe
severely
sharply
shortly
show
showed
shown
showing
siberian
significance
significant
significantly
similar
similarities
similarity
similarly
simple
simplest
simplicities
simplicity
since
slightly
society
society's
sol.
some
somebody
someday
somehow
someone
sometimes
someway
someways
somewhat
somewhere
sort
sorts
spanish
special
specially
specifically
spite
started
starting
still
study
studied
studies
studying
style
subject
subjects
submitted
subscript
subscription
subscriptions
subscripts
subsequent
subsequently
substantial
substantially
subtracted
subtracting
subtraction
subtractions
succeeded
succeeding
successful
successfully
successive
successively
such
suddenly
suggest
suggested
suggester
suggesters
suggestibility
suggestible
suggesting
suggestion
suggestions
suggestive
suggestively
suggestiveness
suggests
suitabilities
suitability
suitable
suitableness
suitably
sufficient
sufficiently
sulph
summarized
summary
supplied
supplying
survey
surveyed

surveying
surveys
sustainable
symbol
symbols
t
t1
t2
table
tables
table1
table.1
table2
tabl.1
taken
taking
talk
talking
talkings
talks
task
tasked
tasking
taskmaster
tasks
temperatures
tend
tended
tendencies
tendency
tends
term
terms
tested
testes
testier
testiest
testified
testifies
testify
testifying
tightly
than
thank
thanked
thankful
thankfully
thankfulness
thanking
thankless
thanklessly
thanks
thanksgiving
that
then
there
thereabouts
thereafter
thereby
therefor
therefore
therefrom
therein
thereof
thereon
thereto
thereunder
thereupon
therewith
these
thesis
thing
things
this
thorough
thoroughly
thoroughness
those
thousand
thousands
tightly
timescale
timescales
title
titled
titles
thus
today
today's
together
ton
tone
tones
tons
topic
topical
topicality
topically
topics
too
took
traditional
traditionally
trend
trends
trouble
troubled
troubles
twice
typical
typically
u.a
university
unclear

uncleared
unchangeable
unchanged
unchanging
understanding
understood
undertaken
undoubtedly
unexpected
unexpectedly
unexplored
unfavorable
unfavourably
unfavoured
unfortunately
union
unique
universal
unless
unlike
unlikely
unsuitability
unsuitable
unsuitableness
unsuitably
until
unusual
unusually
unwanted
usage
usages
use
used
useful
usefully
usefulness
useless
uselessly
uselessness
user
users
uses
using
usual
usually
utilise
utilised
utilises
utilising
utilizing
validated
value
values
varian
variation
varied
variety
various
verifiability
verifiable
verification
verifications
verified
verifier
verifiers
verifies
verify
verifying
verities
verity
varying
very
view
viewable
viewed
viewpoint
viewpoints
views
vigorously
vol.
vpore
was
wavenumber
week
weeks
well
well-suited
were
west
western
what
while
whereas
whereby
wherefore
wherefores
wherein
whereof
whereon
whether
whole
withdrawn
widely
work
work's
world
world's
worldwide
written
wrong
wrt
wrt.
wt.
xx

year

years

yielded

yielding

γ

λ

δ

θ

σ

ø

φ

ε

æ

δh

и

()

γ-al
