# Supplementary material for: Terminology spectrum analysis of natural-language chemical documents: term-like phrases retrieval routine
Source: J Cheminform. 2016 Apr 29;8:22. doi: 10.1186/s13321-016-0136-4 (PMC4850643; doi:10.1186/s13321-016-0136-4)
Supplement: Supplementary file 5 — 10.1186/s13321-016-0136-4 List of stable isotopes. [file 13321_2016_136_MOESM5_ESM.pdf]

Additional file 5
List of stable isotopes

1H
3He
4He
19F
20Ne
21Ne
22Ne
27Al
36Ar
38Ar
40Ar
79Br
81Br
113In
127I
139La
93Nb
16O
17O
18O
31P
14N
15N
127I
130Ba
132Ba
134Ba
135Ba
136Ba
137Ba
138Ba
9Be
10B
11B
51V
182W
183W
184W
186W
154Gd
155Gd
156Gd
157Gd
158Gd
160Gd
69Ga
71Ga
176Hf
177Hf
178Hf
179Hf
180Hf
70Ge
72Ge
73Ge
74Ge
156Dy
158Dy
160Dy
161Dy
162Dy
163Dy
164Dy
153Eu
54Fe
56Fe
57Fe
58Fe
197Au
191Ir
193Ir
89Y
39K
41K
40Ca
42Ca
43Ca
44Ca
46Ca
57Co
59Co

28Si
29Si
30Si
78Kr
80Kr
82Kr
83Kr
84Kr
86Kr
124Xe
126Xe
128Xe
129Xe
130Xe
131Xe
132Xe
134Xe
136Xe
6Li
7Li
175Lu
24Mg
25Mg
26Mg
55Mn
63Cu
65Cu
92Mo
94Mo
95Mo
96Mo
97Mo
98Mo
75As
23Na
142Nd
143Nd
145Nd
146Nd
148Nd
58Ni
60Ni
61Ni
62Ni
64Ni
112Sn
114Sn
115Sn
116Sn
117Sn
118Sn
119Sn
120Sn
122Sn
124Sn
184Os
187Os
188Os
189Os
190Os
192Os
102Pd
104Pd
105Pd
106Pd
108Pd
110Pd
192Pt
194Pt
195Pt
196Pt
198Pt
141Pr
185Re
103Rh
196Hg
198hg
199Hg
200Hg
201Hg
202Hg
85Rb
96Ru
98Ru
99Ru
100Ru
101Ru

102Ru
104Ru
144Sm
149Sm
150Sm
152Sm
154Sm
204Pb
206Pb
207Pb
208Pb
74Se
76Se
77Se
78Se
80Se
107Ag
109Ag
32S
33S
34S
36S
45Sc
84Sr
86Sr
87Sr
88Sr
121Sb
123Sb
203Tl
205Tl
181Ta
120Te
122Te
123Te
124Te
125Te
126Te
159Tb
46Ti
47Ti
48Ti
49Ti
50Ti
169Tm
12C
13C
14C
35Cl
37Cl
50Cr
52Cr
53Cr
54Cr
133Cs
136Ce
138Ce
140Ce
64Zn
66Zn
67Zn
68Zn
70Zn
90Zr
91Zr
92Zr
94Zr
162Er
164Er
166Er
167Er
168Er
170Er
106Cd
108Cd
110Cd
111Cd
112Cd
114Cd
90Sr
