# Supplementary material for: Terminology spectrum analysis of natural-language chemical documents: term-like phrases retrieval routine
Source: J Cheminform. 2016 Apr 29;8:22. doi: 10.1186/s13321-016-0136-4 (PMC4850643; doi:10.1186/s13321-016-0136-4)
Supplement: Supplementary file 6 — 10.1186/s13321-016-0136-4. List of chemical element symbols. [file 13321_2016_136_MOESM6_ESM.pdf]

Additional file 6
List of chemical element symbols

H
He
Li
Be
B
C
N
O
F
Ne
Na
Mg
Al
Si
P
S
Cl
Ar
K
Ca
Sc
Ti
V
Cr
Mn
Fe
Co
Ni
Cu
Zn
Ga
Ge
As
Se
Br
Kr
Rb
Sr
Y
Zr
Nb
Mo
Tc
Ru
Rh
Pd
Ag
Cd
In
Sn
Sb
Te
I
Xe
Cs
Ba
La
Ce
Pr
Nd
Pm
Sm
Eu
Gd
