# Supplementary material for: Terminology spectrum analysis of natural-language chemical documents: term-like phrases retrieval routine
Source: J Cheminform. 2016 Apr 29;8:22. doi: 10.1186/s13321-016-0136-4 (PMC4850643; doi:10.1186/s13321-016-0136-4)
Supplement: Supplementary file 7 — 10.1186/s13321-016-0136-4 List of measurement units. [file 13321_2016_136_MOESM7_ESM.pdf]

Additional file 7

List of measurement units

Ǻ
Å
Å-1
kNm3
rpm
ppm
kg
h.kg
h.kgcat
kg(cat)-1
kg-1
kg-1s-1
kg.h.mol-1
kg.s
g.s
kgcat
kgcat-1
kgcat-1h-1
kgch2.m-3.h-1
molh2o2kgcat-1h-1
kV
keV
kWe
g-1.h-1
g-1cat
g-1h-1
g-1s-1
/g
g-1
grcat
g-cat
g-cat-1
g-cat-1h-1
g-cat-1hour-1
g-catalyst
gcat-1
gcat-1h-1
gcat.h
gcat.min
gcatalyst-1
gcath
h.g
h.gcat
h.kg
h.kgcat
kgcat
kgcat-1
kgcat-1h-1
kgch2.m-3.h-1
l.h-1.gcat-1
mgcat
min-1gcat-1
min.gcat
ml(gcat.min)-1
ml.min-1
ml.min-1.cm-2
ml.min-1.gcat-1
molh2o2kgcat-1h-1
ml.g-1.h-1
ml.g-1.min-1
ml.h-1.g-1
mlmin-1
µl
ml
w.ml-1
ml-1
deg.min-1
mm
mm2
mm3
mm3g-1
nm2
nm-2
nm3
.cm
cm]-1
cm2
cm2min
cm2s-1
cm3min
cm3
cm-1
cm-2
cm-3
cm3.g-1
cm3.min-1
cm3g
cm3g-1
cm3g-1h-1
cm3min-1
dm3
dm3h-1
m-1

m-2
m-3
/mm
/nm
/cm
hr
/h
(h)
h-1
h-1g
h-1g-1
h-1)
hour
/min
min-1
/s
s-1
kJ
kj.mol-1
kjmol-1
m3mol-1
dmol3
atm
atm-1
mol
mol-1
mol.l-1
mol.l-1.min-1.g-1
mol.h-1.g-1
mol.s
mols
mols-1
moll-1
mol-1k-1
mol.dm
mol.dm-3
mol.h
mol.h-1
mol.h-1.g-1
mmol
mmols
µmols
mmol-1
µmol-1
µmol
mmol.g
µmol.g
mmol.g-1
µmol.g-1
mmolg-1
µmolg-1
mmol.gcat-1
µmol.gcat-1
mmol.g-1.h-1
µmol.g-1.h-1
mmol.h-1
µmol.h-1
mmol.h-1.g-1
µmol.h-1.g-1
mmol.l-1
µmol.l-1
mmol.l-1.min-1
µmol.l-1.min-1
mmol.min
µmol.min
mmol.m-2
µmol.m-2
mmol.s-1
µmol.s-1
mmol.cm-1.s-1
µmol.cm-1.s-1
mmole
µmole
mmoles
µmoles
mmoleg-1
nmol
nmol.g-1.s-1
kmol
ghmol-1
gmol
kcal.mol-1
l.mol-1.s-1
l.h-1.g-1cat
l.h-1.gcat-1
l.min-1
kg.h.mol-1
m2.g-1
m2g-1
mA.mg-1
mg-1
m3mol-1
km
meq
torr
time(min)
time,min
time[min]
bar
mbar
/pts
(a.u.)
a.u
(ev)
ev

e.v
ºC
ºC
˚C
°C
ºC.min-1
.g
.g-1
g.h
g.min
gcatalyst
gcat
gcat h
(gcat h)-1
g.cat
g.cat.h
g.gzeolite-1.h-1
g.l-1
g.s.cm-3
MCAT
cat-1
cat)-1
kPa
gHz
kHz
MHz
µm
µm2
µm3
patm
p(atm)
ampere
Ångstrom
ångström
atmosphere
barn
becquerel
bohr
calorie
candela
clausius
coulomb
curie
dalton
day
debye
decibel
degree Celsius
degree Fahrenheit
degree
dyne
einstein
electronvolt
entropy unit
farad
foot
franklin
gauss
gram
gray
henry
hertz
inch
joule
katal
kelvin
kilogram
litre
lumen
metre
millimetre of mercury
minute
mole
nanometer
newton
ohm
pascal
poise
rad
radian
röntgen
second
siemens
sievert
steradian
stokes
tesla
tonne
tons
volt
watt
weber
yard
year
